# Pd(II)–NHC (NHC = N‐Heterocyclic Carbene)‐Catalyzed Alkylation of Primary Amides by Site‐Selective N─C(O) Activation

**DOI:** 10.1002/advs.202511827

**Published:** 2025-10-06

**Authors:** Peng Lei, Yuge Hu, Deshuang Zhang, Jiuhui Ye, Yanqing Gao, Juntao Feng, Zhiqing Ma, Xili Liu, Michal Szostak

**Affiliations:** ^1^ College of Plant Protection Northwest A&F University No.3 Taicheng Road Yangling Shaanxi 712100 China; ^2^ Shaanxi Research Center of Biopesticide Engineering & Technology Northwest A&F University No.3 Taicheng Road Yangling Shaanxi 712100 China; ^3^ State Key Laboratory for Crop Stress Resistance and High‐Efficiency Production Northwest A&F University No.3 Taicheng Road Yangling Shaanxi 712100 China; ^4^ Department of Chemistry Rutgers University 73 Warren Street Newark NJ 07102 United States

**Keywords:** alkylation, N–C(O) activation, N‐heterocyclic carbenes, Pd–catalysis, primary amides

## Abstract

Although over the past decade, there have been tremendous advances in amide bond cross‐coupling, one of the major remaining challenges is selective alkylation of ubiquitous primary amides. Herein, a highly efficient method for Pd(II)–NHC (NHC = N‐heterocyclic carbene)‐catalyzed alkylation of primary amides using alkyl organozinc reagents is reported. The method is distinguished by the use of practical, bench‐stable Pd(II)–NHC precatalysts, where the sterically‐hindered and readily accessible IPr^*^ is found to be privileged for facilitating this highly challenging alkylation. All substrates used are common primary amides that are ubiquitous through all facets of chemical science. Comparative studies, application to the synthesis of pharmaceutical intermediates, scope of diverse acyl precursors and one‐pot activation/cross‐coupling are described. Considering the importance of primary amides in all fields of chemistry and the practical access to Pd(II)–NHC complexes, this C(O)─N alkylation approach offers a broadly applicable avenue for amide bond interconversion pathways.

## Graphical abstract

A highly efficient method for Pd(II)–NHC (NHC = N‐heterocyclic carbene)‐catalyzed alkylation of primary amides using alkyl organozinc reagents is reported. The sterically‐hindered and readily accessible IPr^*^ is found to be privileged for facilitating this highly challenging alkylation. Comparative studies, application to the synthesis of pharmaceutical intermediates, scope of diverse acyl precursors and one‐pot activation/cross‐coupling are described.

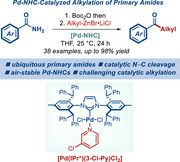

## 1 Introduction

**Figure 1:**
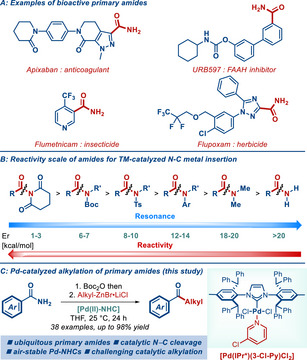
A) Examples of bioactive primary amides. B) Reactivity scale of amides for TM‐catalyzed N‐C metal insertion. C) [Pd(II)‐NHC]‐catalyzed alkylation of primary amides (this study).

In the last decade, amide bond activation has emerged as a powerful manifold for the previously elusive amide bond interconversion.^[^ ^1^, ^2^, ^3^, ^4^, ^5^, ^6^, ^7^, ^8^, ^9^, ^10^, ^11^, ^12^ ^]^ This paradigm exploits breaking of amidic resonance (n_N_→*π* ^*^_C = O_ conjugation, typically 15–20 kcal mol^−1^ in planar amides) by amide bond twisting, electronic delocalization and activating group conjugation.^[^ ^13^, ^14^, ^15^, ^16^, ^17^, ^18^ ^]^ In particular, the transition‐metal‐catalyzed formation of acyl–metal intermediates directly from amides by N─C(O) bond oxidative addition has enabled an unprecedented access to a broad range of previously inaccessible reaction pathways, including C─C, C─N, C─B, C─Si, and C─S bond formation.^[^ ^6^, ^7^, ^8^, ^9^, ^10^, ^11^, ^12^ ^]^ Considering the utmost importance of amides as fundamental functional groups in synthesis, biology, biochemistry and in particular drug discovery, where amide bond manipulation reactions are present in >50% of research projects and amide bonds are core structures of >30% of new drug candidates, this N─C(O) activation mechanism provides a highly innovative pathway to amide bond diversification.^[^ ^19^, ^20^, ^21^ ^]^ In this context, primary amides are the most fundamental of amide derivatives in pharmaceuticals, synthetic intermediates, agrochemicals and functional materials (**Figure** **1**A).^[^ ^22^, ^23^, ^24^, ^25^ ^]^ However, owing to the planar nature of the primary amide bond, the direct activation of primary amides by a controlled metal insertion into the acyl bond has been a major challenge (Figure 1B).^26^, ^27^, ^28^, ^29^ Similarly, although many approaches to amide N─C(O)/C(sp^2^) cross‐coupling have been established, the analogous and potentially more important N─C(O)/C(sp^3^) cross‐coupling has been a daunting task.^[^ ^1^, ^2^, ^3^, ^4^, ^5^, ^6^, ^7^, ^8^, ^9^, ^10^, ^11^, ^12^, ^26^, ^27^, ^28^, ^29^ ^]^ To date, only one report successfully managed to address Ni‐catalyzed alkylation using organozinc reagents; however, this process uses secondary amide derivatives and is not suitable for primary amides.^[^ ^30^ ^]^ As a part of our program in amide bond activation, herein, we report a highly efficient method for Pd(II)–NHC‐catalyzed alkylation of primary amides using alkyl organozinc reagents (Figure 1C). There are several noteworthy findings of our study: 1) The method is distinguished by the use of practical, bench‐stable Pd(II)–NHC precatalysts, where the sterically‐hindered and readily accessible IPr^*^ has been found to be privileged for facilitating this highly challenging alkylation. 2) All substrates used are common primary amides that are ubiquitous through all facets of chemical science. 3) Comparative studies, application to the synthesis of pharmaceutical intermediates, scope of diverse acyl precursors and one‐pot activation/cross‐coupling are described. Overall, considering the importance of primary amides in all fields of chemistry and the practical access to Pd(II)–NHC complexes, this C(O)─N alkylation approach offers a broadly applicable avenue for amide bond interconversion pathways.^[^ ^31^, ^32^, ^33^, ^34^, ^35^ ^]^

In general, the main challenge in alkylation of amide derivatives stems from the significantly more difficult transmetallation with alkyl nucleophiles, where the alkyl–metals undergo facile *β*‐hydride elimination, protodemetallation and homocoupling compared to their aryl counterparts.^[^ ^36^, ^37^ ^]^ Our group has established that imdazol‐2‐ylidenes, such as IPr, are vastly preferred for N─C(O)/C(sp^2^) cross‐couplings.^[^ ^38^, ^39^, ^40^, ^41^, ^42^, ^43^, ^44^, ^45^ ^]^ Furthermore, our group determined that select Pd(II)–NHC precatalysts are more favored than others owing to the facility of the activation to monoligated Pd(0)–NHC and the reversible stabilization of palladium by the throw‐away ancillary ligand.^[^ ^46^, ^47^, ^48^, ^49^, ^50^ ^]^ Following these studies, we hypothesized that a tuning of the N‐heterocyclic carbene ligand system in both the NHC ligand and the ancillary ligand might enable the elusive general Pd‐catalyzed alkylation of amide derivatives. Previous studies have been significantly limited in scope owing to inefficient stabilization of the reactive metal centers, slow transmetallation and overwhelming side reactions, prohibiting application of this attractive research platform.^[^ ^38^, ^39^, ^40^, ^41^, ^42^, ^43^, ^44^, ^45^, ^46^, ^47^, ^48^, ^49^, ^50^ ^]^ Furthermore, in terms of amide bond resonance breaking, studies established that the Ni(0)–SIPr system is particularly effective for N–Ts amides, where amidic resonance is disrupted by N to S(O)_2_R conjugation, while the use of N‐*tert*‐butoxycarbonyl activation required additional steric hindrance in line with the higher electrophilicity of N–Ts amides.^[^ ^30^ ^]^ Based on previous studies, we aimed to exploit the more challenging N–Boc_2_ amides, which are readily accessible from the ubiquitous primary amides by N‐site‐selective double *tert*‐butoxycarbonylation, thus enabling the elusive alkylative cross‐coupling of primary amides.^[^ ^29^, ^51^, ^52^ ^]^ An additional feature of our approach was the use of bench‐, air‐ and moisture‐stable Pd(II)–NHC precatalysts. Studies by Nolan and co‐workers clearly established that these catalysts are vastly preferred over the in situ formed Pd(0)–NHCs, while providing an important practical advantage for all interested users over the more unstable systems.^[^ ^53^, ^54^, ^55^, ^56^, ^57^, ^58^, ^59^, ^60^, ^61^ ^]^

## 2 Results and Discussion

Our studies commenced with the evaluation of cross‐coupling of benzamide (1a) with benzylzinc bromide (2a) in the presence of various Pd(II)–NHC precatalysts (**Table** **1**). It should be noted that this optimization revealed a facile two‐step process, where N,N‐Boc_2_‐activated amides are readily synthesized from unactivated and electronically‐unbiased benzamides.^[^ ^29^, ^51^, ^52^ ^]^ Among various Pd(II)–NHC screened, such as heterocycle‐ and allyl‐based imidazol‐2‐ylidenes and imidazolin‐2‐ylidenes, [Pd(IPr)(3‐Cl‐Py)Cl_2_] (entry 1), [Pd(SIPr)(3‐Cl‐Py)Cl_2_] (entry 2), [Pd(IPr^*^)(3‐Cl‐Py)Cl_2_] (entry 3), [Pd(IPr)(cin)Cl] (entry 4), [Pd(SIPr)(cin)Cl] (entry 5), the initial positive result was obtained using a sterically‐hindered IPr^*^ ligand reported first by Marko and co‐workers with benzylidene wingtips at the *ortho*‐positions of the aromatic ring.^[^ ^62^ ^]^ This catalyst was selected for further optimization, which focused on the effect of additives (entries 6–16), stoichiometry (entry 17) and the organozinc reagent (entries 18–20). After extensive optimization, we found that the optimal conditions involve a small excess of the LiCl complexed organozinc bromide (1.5 equiv) in the presence of [Pd(IPr^*^)(3‐Cl‐Py)Cl_2_] precatalyst in THF at room temperature. Under these conditions, we did not observe the competing cleavage of the N–Boc group deactivating the amide bond and the homocoupling of the organozinc reagent.

**Table 1:** Optimization of the reaction conditions.

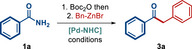				
Entry^a)^	Catalyst	[Pd‐NHC] [x mol%]	Additive	Yield [%]^b)^
1	[Pd(IPr)(3‐Cl‐Py)Cl_2_]	5	/	<3
2	[Pd(SIPr)(3‐Cl‐Py)Cl_2_]	5	/	<3
3	[Pd(IPr^*^)(3‐Cl‐Py)Cl_2_]	5	/	4
4	[Pd(IPr)(cin)Cl]	5	/	<3
5	[Pd(SIPr)(cin)Cl]	5	/	<3
6	[Pd(IPr^*^)(3‐Cl‐Py)Cl_2_]	5	NaCl	8
7	[Pd(IPr^*^)(3‐Cl‐Py)Cl_2_]	5	KCl	6
8	[Pd(IPr^*^)(3‐Cl‐Py)Cl_2_]	5	KF	7
9	[Pd(IPr^*^)(3‐Cl‐Py)Cl_2_]	5	K_2_CO_3_	4
10	[Pd(IPr^*^)(3‐Cl‐Py)Cl_2_]	5	K_3_PO_4_	9
11	[Pd(IPr^*^)(3‐Cl‐Py)Cl_2_]	5	KOH	11
12	[Pd(IPr^*^)(3‐Cl‐Py)Cl_2_]	5	LiOH	7
13	[Pd(IPr^*^)(3‐Cl‐Py)Cl_2_]	5	LiCl	16
14	[Pd(IPr^*^)(3‐Cl‐Py)Cl_2_]	5	Cs_2_CO_3_	10
15	[Pd(IPr^*^)(3‐Cl‐Py)Cl_2_]	5	LiHMDS	8
16	[Pd(IPr^*^)(3‐Cl‐Py)Cl_2_]	5	KHMDS	<3
17	[Pd(IPr^*^)(3‐Cl‐Py)Cl_2_]	10	LiCl	24
18^c)^	[Pd(IPr^*^)(3‐Cl‐Py)Cl_2_]	10	/	87
19^c)^	[Pd(IPr^*^)(3‐Cl‐Py)Cl_2_]	5	/	85
20^c)^	[Pd(IPr^*^)(3‐Cl‐Py)Cl_2_]	1	/	82

^a)^ Conditions: amide (1.0 equiv), Bn‐ZnBr (1.5 equiv), additive (1.5 equiv), [Pd‐NHC] (5 mol%), THF, 25 °C, 24 h;

^b)^ GC/^1^H NMR yields;

^c)^ Replace Bn‐ZnBr with Bn‐ZnBr·LiCl.

**Figure 2:**
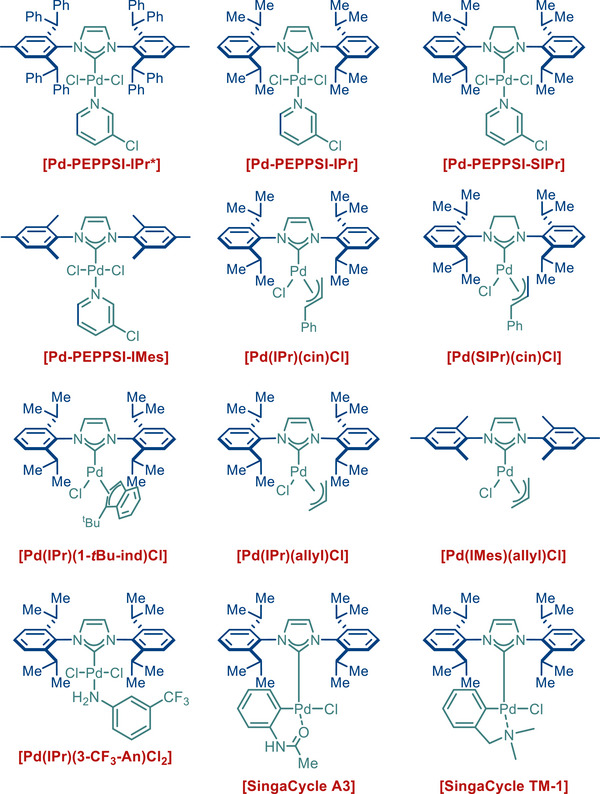
Structures of Pd(II)‐NHC precatalysts.

Next, we evaluated the effect of various Pd(II)–NHC precatalysts (**Figure** **2** and **Table** **2**). As shown, we established that although [Pd(IPr^*^)(3‐Cl‐Py)Cl_2_] (entry 1) is the preferred catalyst, several other Pd(II)–NHCs gave promising results in this challenging cross‐coupling. In general, heterocycle‐based catalysts are preferred (entries 1–4), which is likely due to the non‐innocent role of the pyridine ligand in stabilizing Pd(0)–NHC by re‐coordination. It is worth noting that steric‐hindrance of the NHC ligand is absolutely required for this coupling (entries 1–3 vs entry 4). Cinnamyl‐ and indenyl‐based catalysts, such as [Pd(IPr)(cin)Cl], [Pd(SIPr)(cin)Cl], [Pd(IPr)(*t*‐Bu‐ind)Cl] (entries 5–7) performed well in this cross‐coupling, however, their allyl‐based congeners, [Pd(IPr)(allyl)Cl], [Pd(IMes)(allyl)Cl] (entries 8–9) were ineffective, suggesting the role of allyl ligand in activation to Pd(0)–NHC. Furthermore, aniline‐based [Pd(IPr)(3‐CF_3_‐An)Cl_2_] (An = aniline) as well as palladacycle‐based SingaCycle A3, SingaCycle TM‐A1 (entries 10–12) afforded the cross‐coupling product in moderate to good yields, establishing these Pd(II)–NHC precursors as effective in this cross‐coupling. Finally, a representative Pd–phosphine complex, [Pd(PPh_3_)_2_Cl_2_], was tested and found to be completely unreactive under the reaction conditions (entry 13). Overall, these studies highlight the role of strongly σ‐donating and sterically‐hindered NHC ligands to promote challenging transmetallation in this alkylative process. It should be noted that studies with reduced catalyst loading demonstrate that the standard coupling is feasible at 5 mol% loading (85% yield, Table 2, entry 1) and even 1 mol% loading (82% yield, Table 2, entry 1). Importantly, studies with reduced catalyst loading further demonstrate the advantage of sterically‐demanding *ortho*‐diphenylmethyl catalyst [Pd(IPr^*^)(3‐Cl‐Py)Cl_2_],^[^ ^62^ ^]^ which permits lower catalyst loading, while all other catalysts result in a significant decrease of reaction efficiency. This is consistent with the beneficial steric impact on the stabilization of the mono‐ligated Pd(0)–NHC enabled by bulky aromatic wingtips in the catalyst structure.

**Table 2:** Screening of Pd(II)–NHC precatalysts.

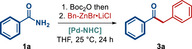		
Entry^a)^	Catalyst	Yield [%]^b)^
1	[Pd(IPr^*^)(3‐Cl‐Py)Cl_2_]	90 / 85^c)^ / 82^d)^
2	[Pd(IPr)(3‐Cl‐Py)Cl_2_]	69 / 33^c)^ / 9^d)^
3	[Pd(SIPr)(3‐Cl‐Py)Cl_2_]	74 / 60^c)^ / 30^d)^
4	[Pd(IMes)(3‐Cl‐Py)Cl_2_]	23
5	[Pd(IPr)(cin)Cl]	55 / 40^c)^ / 11^d)^
6	[Pd(SIPr)(cin)Cl]	45 / 28^c)^ / 9^d)^
7	[Pd(IPr)(*t*‐Bu‐ind)Cl]	76 / 42^c)^ / 12^d)^
8	[Pd(IPr)(allyl)Cl]	3
9	[Pd(IMes)(allyl)Cl]	<3
10	[Pd(IPr)(3‐CF_3_‐An)Cl_2_]	50 / 41^c)^ / 11^d)^
11	SingaCycle A3	70 / 35^c)^ / 14^d)^
12	SingaCycle TM‐A1	29
13	[Pd(PPh_3_)_2_Cl_2_]	0

^a)^ Conditions: Conditions: amide (1.0 equiv), Bn‐ZnBr·LiCl (1.5 equiv), [Pd‐NHC] (10 mol%), THF, 25 °C, 24 h;

^b)^ GC/^1^H NMR yields;

^c)^ [Pd‐NHC] (5 mol%);

^d)^ [Pd‐NHC] (1 mol%).

**Scheme 1:**
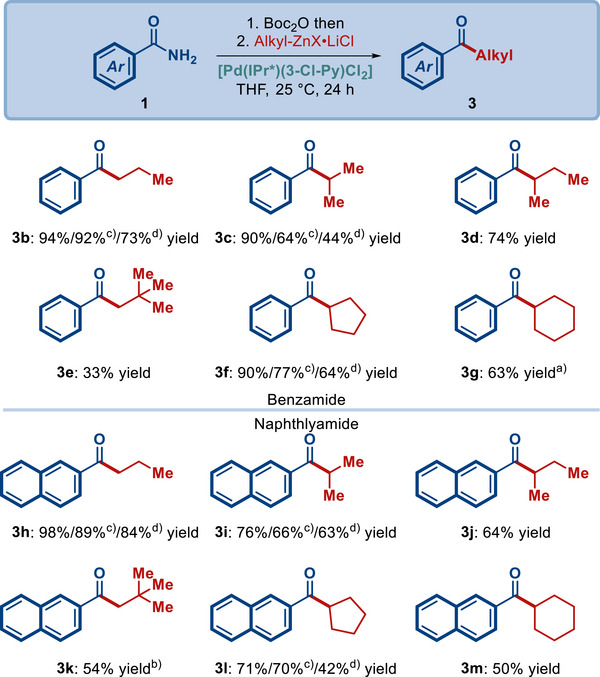
Scope of the organozinc coupling partner. Conditions: amide (1.0 equiv), Alkyl‐ZnX·LiCl (1.5 equiv), [Pd‐NHC] (10 mol%), THF, 25 °C, 24 h. Isolated yields. ^a)^[Pd(IPr)(*t*‐Bu‐ind)Cl] (20 mol%). ^b)^[Pd(IPr)(3‐Cl‐Py)Cl_2_] (20 mol%). ^c)^[Pd‐NHC] (5 mol%). ^d)^[Pd‐NHC] (1 mol%).

With the optimized conditions in hand, we next evaluated the scope of organozinc reagents (**Scheme** **1**). It should be noted that the present process deploys primary amides using significantly more challenging N–Boc_2_ derivatives. As shown, this process can be used to cross‐couple not only benzyl, but also linear alkyl (3b and 3h), α‐branched (3c, 3d, 3i, and 3j) and extremely sterically‐hindered alkyl (3e and 3k) as well as alicyclic alkyl (3f, 3g, 3l and 3m) nucleophiles. In terms of benzamide vs 2‐naphthylamide, in select cases the more activated 2‐naphthylamide gives slightly higher yields (e.g., the very hindered neopentyl nucleophile), however, in general both classes of electrophiles are well‐suitable for this challenging cross‐coupling. Furthermore, it should be noted that this Pd(II)–NHC system performs very well for the challenging primary amide activated N–Boc_2_ amides, which is very rare in amide bond activations, where N–Ts amides are generally significantly more reactive owing to the withdrawing nature of the N–SO_2_R activating group.

**Scheme 2:**
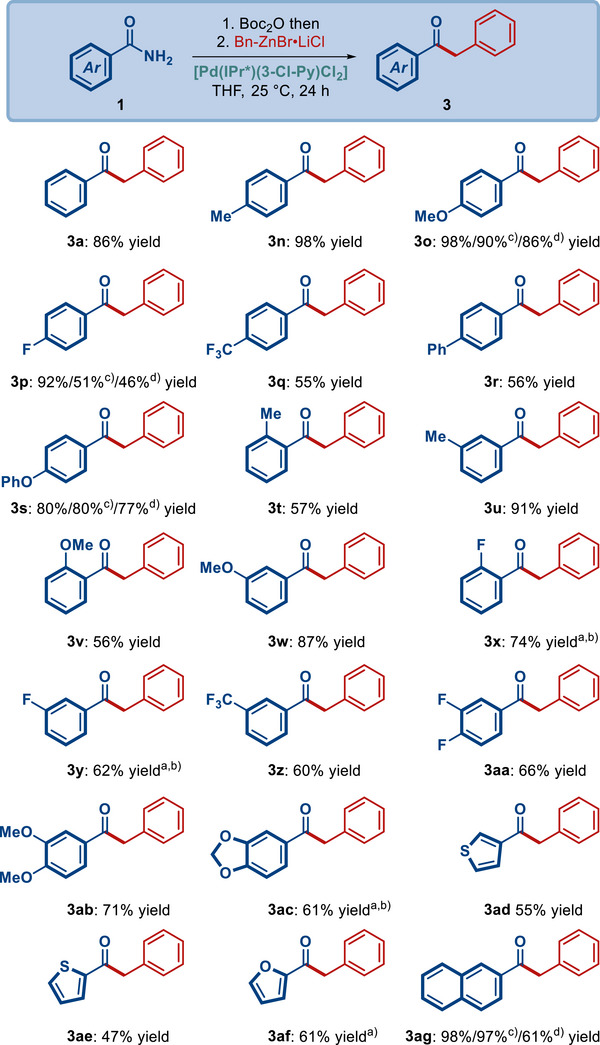
Scope of amide substrates. Conditions: amide (1.0 equiv), Bn‐ZnBr·LiCl (1.5 equiv), [Pd‐NHC] (10 mol%), THF, 25 °C, 24 h. Isolated yields. ^a)^Bn‐ZnBr·LiCl (3.0 equiv). ^b)^[Pd‐NHC] (20 mol%). ^c)^[Pd‐NHC] (5 mol%). ^d)^[Pd‐NHC] (1 mol%).

Next, studies were undertaken to establish the scope of the amide component in this alkylative cross‐coupling (**Scheme** **2**). As shown, this process shows a very broad scope in terms of amide substitution, including electron‐neutral (3a), electron‐donating (3n, 3o, 3s−3w, 3ab), electron‐withdrawing (3p−3r, 3x−3aa) and heterocyclic (3ad−3af) groups. Of note is that fluorinated arenes (3p, 3q, 3x−3aa) that are prevalent in medicinal chemistry settings are well‐compatible as well are the sensitive to cleavage aryl ethers (3s) and heterocyclic (3ad−3af) amides that are common in drug discovery research. Furthermore, representative studies were conducted using low catalyst loading (Scheme 1, 2), including linear alkyl (3b, 3h), *α*‐branched alkyl (3c, 3i) and alicyclic alkyl (3f, 3l) organozinc reagents as well as benzamides substituted with electron‐donating (3o), electron‐withdrawing (3p), cleavage‐sensitive aryl ether (3s) and naphthylamide (3ag). These representative examples covering a range of substrates illustrate that reducing catalyst loading using [Pd(IPr^*^)(3‐Cl‐Py)Cl_2_] catalyst is indeed feasible.

Mechanistically, the oxidative addition of the amide N─C(O) bond to Pd(0)–NHC is facile when promoted by the strongly *σ*‐donating N‐heterocyclic carbenes.^[^ ^40^, ^41^, ^42^, ^43^, ^44^, ^45^, ^55^, ^63^, ^64^ ^]^ The rate limiting step is transmetallation, where the organozinc reagent transfers the alkyl group to palladium.^[^ ^65^, ^66^, ^67^ ^]^ The role of sterically‐demanding yet flexible [Pd(IPr^*^)(3‐Cl‐Py)Cl_2_] is critical to open the metal coordination sphere for access of alkyl nucleophiles, while preventing common side reactions inherent to alkylation, such as β‐hydride elimination, amide reduction and degradation. The reductive elimination is facilitated by the presence of the steric *ortho*‐diphenylmethyl in the catalyst structure.^[^ ^36^, ^37^, ^62^ ^]^ Overall, this general N‐heterocyclic carbene ligand design combines the features of steric and electronic effects that promote the challenging amide alkylation with high generality in the presence of sensitive amide activating groups, which is likely to find broad application in amide bond interconversion.

**Scheme 3:**
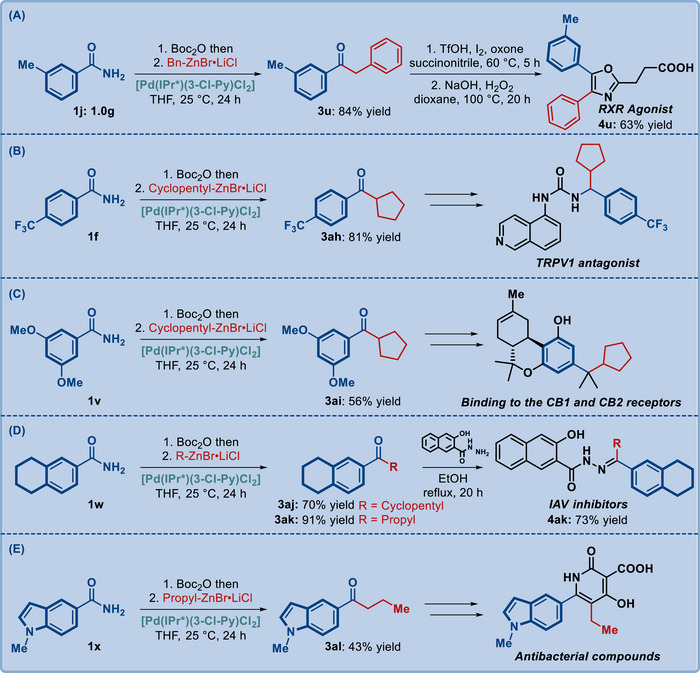
Gram‐scale coupling and the application in the synthesis of pharmaceutical derivatives and their intermediates.

Since primary amides are common and orthogonal synthetic intermediates, we highlighted the application of the present process in the synthesis of pharmaceutical derivatives directly from primary amides (**Scheme** **3**). The first application showcased the alkylative cross‐coupling on a gram scale, demonstrating scalability of this method (Scheme 3A). As shown, this process has been highlighted in the synthesis of the key ketone intermediates to the retinoid X receptors (RXR) agonist, Transient receptor potential vanilloid 1 (TRPV1) antagonist, CB1/CB2 receptor binders, influenza A viruses (IAV) inhibitors and antibacterial compounds, all featuring different amide substitution and various alkyl organozinc reagents (Scheme 3A–E).^[^ ^68^, ^69^, ^70^, ^71^, ^72^ ^]^ This cross‐coupling route provides an attractive alternative to the classical nucleophilic displacement, which typically proceeds in lower yields. To further illustrate the importance of the intermediates prepared by this method, we have completed the synthesis of drug molecules.^[^ ^68^, ^71^ ^]^ Specifically, the synthesis of RXR agonist (Scheme 3A) and IAV inhibitors (Scheme 3D) proceeded uneventfully after the alkylation of the corresponding amide derivatives, establishing a novel approach to the synthesis of these important pharmaceuticals.

**Scheme 4:**
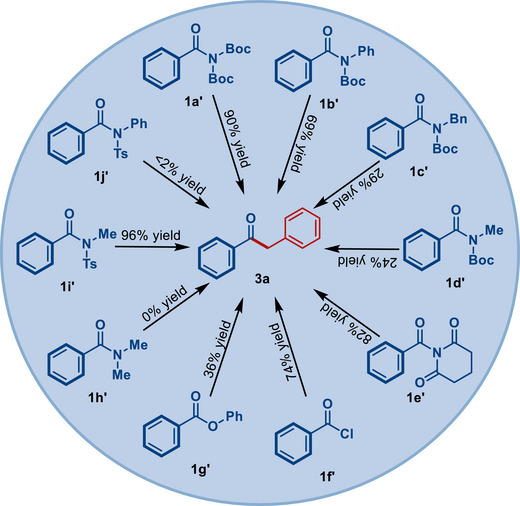
Comparative cross‐coupling of different acyl precursors.

**Scheme 5:**
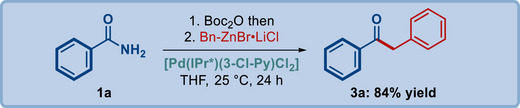
One‐pot N‐Boc‐activation / cross‐coupling.

Furthermore, although our focus was on the cross‐coupling of primary amides, we were keen to gain insight into the comparative cross‐coupling of different acyl precursors (**Scheme** **4**). As shown, this method can be readily applied to various amide and other carboxylic acid derivatives, further establishing the generality of this Pd(II)–NHC‐catalyzed approach. It should be noted that in cases where no reactivity was observed (NMe_2_, NPhTs), the amides could be quantitively recovered, providing a method for chemoselective amide bond activation. Further, it is interesting to note that the challenging N–Boc_2_ amides outperform acyl halides and phenolic esters, further demonstrating the capacity of primary amides as powerful electrophiles in transition‐metal‐catalysis. We also demonstrated that the one‐pot N–Boc double N‐t*ert*‐butoxycarbonylation/alkylative cross‐coupling is possible (**Scheme** **5**). This robustness of the method and the availability of Pd(II)–NHC precatalysts should streamline applications of the present process.

## 3 Conclusion

In conclusion, we have reported the first catalytic alkylative cross‐coupling of primary amide derivatives. This process engages sterically‐hindered and readily accessible Pd(II)–NHC precatalysts and organozinc reagents. The steric‐hindrance and strong σ‐donation of NHC ligands provide steric and electronic environment around the Pd center to facilitate the challenging transmetallation in the previously elusive alkylative cross‐coupling by N─C(O) activation.^[^ ^53^, ^54^, ^73^, ^74^, ^75^, ^76^, ^77^, ^78^, ^79^, ^80^, ^81^ ^]^ The transformation is characterized by a broad substrate scope and a favorable reactivity profile compared with Ni(0)–NHC catalysis. While both systems should be considered complementary for future applications in alkylative cross‐couplings of amides, the unique availability of diverse Pd(II)–NHC precatalysts with various NHC and ancillary throw‐away ligands make this class of catalysts particularly well‐suited for further optimization and tailoring to specific substrate combinations. The system has been further applied to the synthesis of pharmaceutical intermediates as well as diverse acyl precursors. Considering the ubiquity of primary amides, this C(O)─N alkylation offers a promising platform for broadly applicable amide bond interconversions of general synthetic and medicinal interest.

## 4 Experimental Section

### General Procedure for the Preparation of Organozinc Halides

The procedure was modified with reference to that reported by Garg.^[^ ^30^ ^]^ Zn powder (653.8 mg, 10.0 mmol, 2.0 equiv, Sinopharm 95%) and anhydrous LiCl (423.9 mg, 10.0 mmol, 2.0 equiv) were added to a flame‐dried 25 mL round‐bottom flask equipped with a magnetic stir bar and a rubber septum. Subsequently, the flask was heated with a heat gun under high vacuum until the solids became motionless. After cooling to room temperature, argon gas was back‐filled. Freshly distilled THF (4.5 mL) and 1,2‐dibromoethane (22 µL, 0.25 mmol, 0.05 equiv) were added via syringe, and the reaction mixture was heated at 60 °C for 20 min. A solution of TMSCl (6 µL, 0.05 mmol, 0.01 equiv) and I_2_ (6.4 mg, 0.025 mmol, 0.005 equiv) in THF (0.5 mL) were added via syringe, and the reaction mixture was reacted at 60 °C for 20 min. Subsequently, the alkyl halide (5.0 mmol, 1.0 equiv) was slowly added dropwise via syringe under an ice‐bath. Then, when the alkyl halide was benzyl bromide, the reaction was carried out at room temperature for 4 h; for the rest of the reactions, they were carried out at 50 °C for 18 h. The supernatant was transferred to a flame‐dried Schlenk flask via syringe. The concentration of the organozinc halide was determined by iodometric titration following the method of Knochel.^[^ ^82^ ^]^ It should be noted that the use of organozinc reagents with different titers will lead to different yields in subsequent coupling reactions.

### General Procedure for the Alkylation of Amides

An oven‐dried vial equipped with a stir bar was charged with an amide substrate (neat, 1.0 equiv), [Pd(IPr^*^)(3‐Cl‐Py)Cl_2_] (typically, 10 mol%), and Alkyl‐ZnBr·LiCl (1.5 equiv, solution in THF), placed under a positive pressure of argon, and subjected to three evacuation/backfilling cycles under high vacuum. The reaction mixture was stirred at 25 °C for 24 h. After the indicated time, the reaction mixture was diluted with CH_2_Cl_2_ (10 mL), filtered, and concentrated. The sample was analyzed by ^1^H NMR (CDCl_3_, 400 or 500 MHz) and GC‐MS to obtain conversion, selectivity and yield using internal standard and comparison with authentic samples. Purification by chromatography on silica gel (EtOAc/petroleum ether = 1:10) afforded the title product (Supporting Information).

## Conflict of Interest

The authors declare no conflict of interest.

## Supporting information



Supporting Information

## Data Availability

The data that support the findings of this study are available in the supplementary material of this article.
