# Supplementary material for: Pd(II)–NHC (NHC = N‐Heterocyclic Carbene)‐Catalyzed Alkylation of Primary Amides by Site‐Selective N─C(O) Activation
Source: Adv Sci (Weinh). 2025 Oct 6;12(47):e11827. doi: 10.1002/advs.202511827 (PMC12713102; doi:10.1002/advs.202511827)

Supporting Information

Pd(II)–NHC (NHC = N-Heterocyclic Carbene)-Catalyzed Alkylation of Primary Amides by Site-Selective N–C(O) Activation

Peng Lei, Yuge Hu, Deshuang Zhang, Jiuhui Ye, Yanqing Gao, Juntao Feng, Zhiqing Ma,* Xili Liu,* and Michal Szostak**

Table of Contents S1

List of Known Compounds/General Methods S2
Experimental Procedures and Characterization Data S4
General Procedure for the Preparation of Organozinc Halides S4
General Procedure for the Alkylation of Primary Amides S5
Characterization Data of Alkylation Products of Primary Amides S7
Scale-up Reaction S23
Application of the Synthesis of Pharmaceutical Derivatives and Their Intermediates S24
Comparative Cross-Coupling of Different Acyl Precursors S29
One-Pot Reaction S30
References S31
^{1}H and ^{13}C NMR Spectra S34

List of Known Compounds/General Methods

All organozinc reagents reported in the manuscript have been prepared by the method reported previously.[1-2] Zn powder (95%) was purchased from Sinopharm Chemical Reagent. All other chemicals were purchased at the highest commercial grade from Adamas-beta, Bide Pharmatech, Aladdin, Macklin, Heowns, Energy Chemical or Laajoo, and used as received. All solvents were purchased at the highest commercial grade and used as received. All solvents were deoxygenated prior to use. All experiments were performed using standard Schlenk techniques under nitrogen or argon unless stated otherwise. Reaction glassware was oven-dried at 140 °C for at least 24 h or flame-dried prior to use, allowed to cool under vacuum and purged with argon (three cycles). All products were identified using ^{1}H NMR analysis and comparison with authentic samples. GC and/or GC/MS analysis was used for volatile products. All yields refer to yields determined by ^{1}H NMR and/or GC or GC/MS using an internal standard (optimization) and isolated yields (preparative runs) unless stated otherwise. ^{1}H NMR and ^{13}C NMR spectra were recorded in $CDCl_3$ or DMSO-d_6 on Bruker spectrometers at 500 (^{1}H NMR) and 125 MHz (^{13}C NMR) or 400 (^{1}H NMR) and 100 MHz (^{13}C NMR). ^{19}F NMR was referenced to spectrometer indirect referencing. All shifts are reported in parts per million (ppm) relative to residual $CDCl_3$ or DMSO-d_6 peak or trimethylsilane (TMS) as internal standard. All coupling constants (J) are reported in hertz (Hz). Abbreviations are: s, singlet; d, doublet; t, triplet; q, quartet; brs, broad singlet; m, multiplet. GC-MS chromatography was performed using a Thermo Trace GC Ultra System coupled with a Polaris Q MS detector using helium as the carrier gas at a flow rate of 1 mL/min and an initial oven temperature of 50 °C. The injector temperature was 250 °C. The detector temperature was 250 °C. For runs with the initial oven temperature of 50 °C, temperature was increased with a 10 °C/min ramp after 50 °C hold for 3 min to a final temperature of 220 °C, then hold at 220 °C for 15 min (splitless mode of injection, total run time of 22.0 min). High-resolution mass spectra (HRMS) were measured on an AB SCIEX TripleTOF5600+ instrument. All flash chromatography was performed using silica gel, 60 Å, 300 mesh. TLC analysis was carried out on glass plates coated with silica gel 60 F254, 0.2 mm thickness. The plates were visualized using a 254 nm ultraviolet lamp or aqueous potassium permanganate solutions. ^{1}H NMR and ^{13}C NMR data are given for all compounds in the Supplementary Experimental for characterization purposes. ^{1}H NMR, ^{13}C NMR and HRMS data

are given for all new compounds. All products have been previously reported, unless stated otherwise.

Experimental Procedures and Characterization Data

General Procedure for the Preparation of Organozinc Halides. The procedure was modified with reference to that reported by Garg.[1] Zn powder (653.8 mg, 10.0 mmol, 2.0 equiv, Sinopharm 95%) and anhydrous LiCl (423.9 mg, 10.0 mmol, 2.0 equiv) were added to a flame-dried 25 mL round-bottom flask equipped with a magnetic stir bar and a rubber septum. Subsequently, the flask was heated with a heat gun under high vacuum until the solids became motionless. After cooling to room temperature, argon gas was back-filled. Freshly distilled THF (4.5 mL) and 1,2-dibromoethane (22 μL, 0.25 mmol, 0.05 equiv) were added via syringe, and the reaction mixture was heated at 60 °C for 20 min. A solution of TMSCl (6 μL, 0.05 mmol, 0.01 equiv) and I_2 (6.4 mg, 0.025 mmol, 0.005 equiv) in THF (0.5 mL) were added via syringe, and the reaction mixture was reacted at 60 °C for 20 minutes. Subsequently, the alkyl halide (5.0 mmol, 1.0 equiv) was slowly added dropwise via syringe under an ice-bath. Then, when the alkyl halide was benzyl bromide, the reaction was carried out at room temperature for 4 hours; for the rest of the reactions, they were carried out at 50 °C for 18 hours. The supernatant was transferred to a flame-dried Schlenk flask via syringe. The concentration of the organozinc halide was determined by iodometric titration following the method of Knochel.[3] It should be noted that the use of organozinc reagents with different titers will lead to different yields in subsequent coupling reactions.

Note: In entries 1-17 of the reaction condition optimization table, $PhCH_2$-ZnBr was used. The preparation procedure for $PhCH_2$-ZnBr simply involves omitting the addition of anhydrous LiCl from the aforementioned procedure.

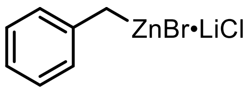

(**2a**, 0.50–0.71 M)

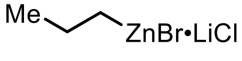

(**2b**, 0.45–0.50 M)

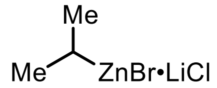

(**2c**, 0.34–0.41 M)

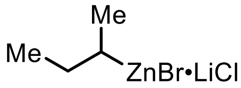

(**2d**, 0.28 M)

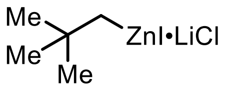

(**2e**, 0.25–0.50 M)

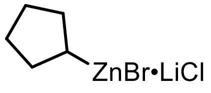

(**2f**, 0.35–0.41 M)

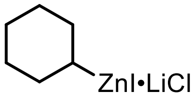

(**2g**, 0.20–0.66 M)

General Procedure for the Alkylation of Primary Amides. An oven-dried vial equipped with a stir bar was charged with an amide substrate (neat, 1.0 equiv), [Pd(IPr*)(3-Cl-Py)Cl_2] (typically, 10 mol%), and Alkyl-ZnBr·LiCl (1.5 equiv, solution in THF), placed under a positive pressure of argon, and subjected to three evacuation/backfilling cycles under high vacuum. The reaction mixture was stirred at 25 °C for 24 h. After the indicated time, the reaction mixture was diluted with CH_2Cl_2 (10 mL), filtered, and concentrated. The sample was analyzed by ^{1}H NMR ($CDCl_3$, 400 MHz or 500 MHz) and GC-MS to obtain conversion, selectivity and yield using internal standard and comparison with authentic samples. Purification by chromatography on silica gel (EtOAc/petroleum ether = 1:10) afforded the title product. The spectra of the title product are >95% pure as determined by ^{1}H NMR.

Note: Monitoring reaction yield by ^{1}H NMR: Use 1,1,2,2-tetrachloroethane as the internal standard. The ^{1}H NMR spectrum was recorded. The yield was determined by integrating characteristic peaks of the product. Monitoring reaction yield by GC: Use 4-phenyltoluene as the internal standard. The GC analysis was conducted. The yield was determined from the peak-area ratio of the product (response factor = 1.0). If response factors differed significantly, the correction factor was determined using a known concentration product and internal standard. Note that in general ^{1}H NMR is the preferred method of analysis with the GC determination as a secondary method.

Representative Procedure for the Alkylation of Primary Amides. An oven-dried vial equipped with a stir bar was charged with *N*,*N*-Boc_2-benzamide (0.2 mmol, 64.3 mg, 1.0 equiv), [Pd(IPr*)(3-Cl-Py)Cl_2] (24.0 mg, 10 mol%), and $PhCH_2$-ZnBr·LiCl (0.48 mL, 0.625 M in THF, 1.5 equiv), placed under a positive pressure of argon, and subjected to three evacuation/backfilling cycles under high vacuum. The reaction mixture was stirred at 25 °C for 24 h. After the indicated time, the reaction mixture was diluted with CH_2Cl_2 (10 mL), filtered, and concentrated. The sample was analyzed by ^{1}H NMR ($CDCl_3$, 400 MHz) and GC-MS to obtain conversion, selectivity and yield using internal standard and comparison with authentic samples. Purification by chromatography on silica gel (EtOAc/petroleum ether = 1:10) afforded the title product. Yield 86% (33.9 mg). White solid. ^{1}H NMR (400 MHz, $CDCl_3$) δ 8.00 (d, *J* = 7.4 Hz, 2H), 7.55 – 7.50 (m, 1H), 7.43 (t, *J* = 7.6 Hz, 2H), 7.34 – 7.28 (m, 2H), 7.27 – 7.24 (m,

3H), 4.26 (s, 2H). ^{13}C NMR (101 MHz, $CDCl_3$) δ 197.70, 136.63, 134.61, 133.25, 129.55, 128.74, 128.72, 128.68, 126.96, 45.55.

Characterization Data of Alkylation Products of Primary Amides

1,2-Diphenylethan-1-one (3a) (Scheme 1)

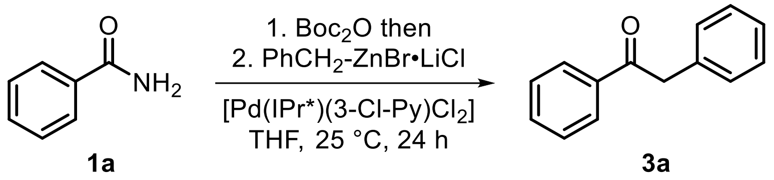

According to the general procedure, the reaction of *N*,*N*-Boc_2-benzamide (1.0 equiv), [Pd(IPr*)(3-Cl-Py)Cl_2] (10 mol%), and $PhCH_2$-ZnBr·LiCl (1.5 equiv, 0.625 M in THF) was stirred at 25 °C for 24 h. Purification by chromatography on silica gel (EtOAc/petroleum ether = 1:10) afforded the title product in 86% yield (33.9 mg). White solid. ^{1}H NMR (400 MHz, $CDCl_3$) δ 8.00 (d, *J* = 7.4 Hz, 2H), 7.55 – 7.50 (m, 1H), 7.43 (t, *J* = 7.6 Hz, 2H), 7.34 – 7.28 (m, 2H), 7.27 – 7.24 (m, 3H), 4.26 (s, 2H). ^{13}C NMR (101 MHz, $CDCl_3$) δ 197.70, 136.63, 134.61, 133.25, 129.55, 128.74, 128.72, 128.68, 126.96, 45.55. The characterization data matched those previously reported.[1]

1-Phenylbutan-1-one (3b) (Scheme 1)

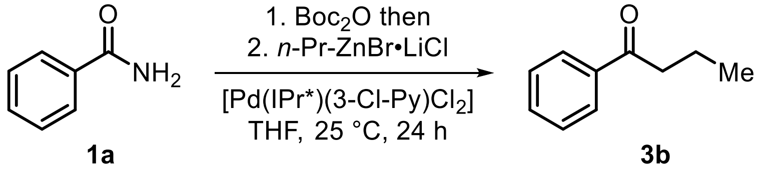

According to the general procedure, the reaction of *N*,*N*-Boc_2-benzamide (1.0 equiv), [Pd(IPr*)(3-Cl-Py)Cl_2] (10 mol%), and *n*-Pr-ZnBr·LiCl (1.5 equiv, 0.50 M in THF) was stirred at 25 °C for 24 h. Purification by chromatography on silica gel (EtOAc/petroleum ether = 1:10) afforded the title product in 94% yield (27.8 mg). White solid. ^{1}H NMR (400 MHz, $CDCl_3$) δ 7.89 (d, *J* = 6.9 Hz, 2H), 7.48 (t, *J* = 7.4 Hz, 1H), 7.39 (t, *J* = 7.6 Hz, 2H), 2.88 (t, *J* = 7.3 Hz, 2H), 1.70 (h, *J* = 7.4 Hz, 2H), 0.94 (t, *J* = 7.4 Hz, 3H). ^{13}C NMR (126 MHz, $CDCl_3$) δ 199.48, 136.11, 131.85, 127.53, 127.03, 39.51, 16.77, 12.87. The characterization data matched those previously reported.[1]

2-Methyl-1-phenylpropan-1-one (3c) (Scheme 1)

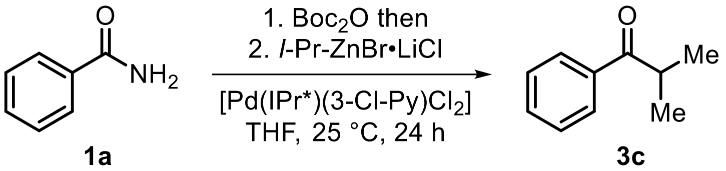

According to the general procedure, the reaction of *N*,*N*-Boc_2-benzamide (1.0 equiv), [Pd(IPr*)(3-Cl-Py)Cl_2] (10 mol%), and *i*-Pr-ZnBr·LiCl (1.5 equiv, 0.39 M in THF) was stirred at 25 °C for 24 h. Purification by chromatography on silica gel (EtOAc/petroleum ether = 1:10) afforded the title product in 90% yield (26.6 mg). Yellow oil. ^{1}H NMR (400 MHz, $CDCl_3$) δ 7.99 – 7.90 (m, 2H), 7.60 – 7.47 (m, 1H), 7.47 – 7.37 (m, 2H), 3.53 (p, *J* = 6.8 Hz, 1H), 1.20 (d, *J* = 7.0 Hz, 6H). ^{13}C NMR (101 MHz, $CDCl_3$) δ 204.26, 136.12, 132.76, 128.58, 128.26, 35.24, 19.11. The characterization data matched those previously reported.[4]

2-Methyl-1-phenylbutan-1-one (3d) (Scheme 1)

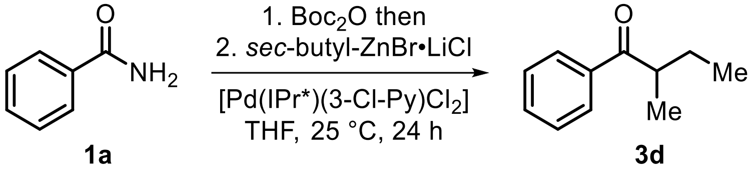

According to the general procedure, the reaction of *N*,*N*-Boc_2-benzamide (1.0 equiv), [Pd(IPr*)(3-Cl-Py)Cl_2] (10 mol%), and *sec*-butyl-ZnBr·LiCl (1.5 equiv, 0.28 M in THF) was stirred at 25 °C for 24 h. Purification by chromatography on silica gel (EtOAc/petroleum ether = 1:10) afforded the title product in 74% yield (24.1 mg). Yellow oil. ^{1}H NMR (400 MHz, $CDCl_3$) δ 7.99 – 7.93 (m, 2H), 7.59 – 7.51 (m, 1H), 7.46 (m, 2H), 3.41 (h, *J* = 6.7 Hz, 1H), 1.89 – 1.79 (m, 1H), 1.56 – 1.43 (m, 1H), 1.19 (d, *J* = 6.9 Hz, 3H), 0.92 (t, *J* = 7.4 Hz, 3H). ^{13}C NMR (101 MHz, $CDCl_3$) δ 204.50, 136.82, 132.80, 128.61, 128.25, 42.12, 26.68, 16.78, 11.80. The characterization data matched those previously reported.[5]

3,3-Dimethyl-1-phenylbutan-1-one (3e) (Scheme 1)

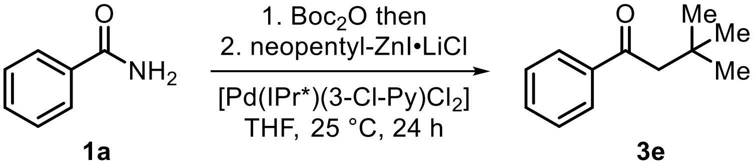

According to the general procedure, the reaction of *N*,*N*-Boc_2-benzamide (1.0 equiv), [Pd(IPr*)(3-Cl-Py)Cl_2] (10 mol%), and neopentyl-ZnI·LiCl (1.5 equiv, 0.25 M in THF) was stirred at 25 °C for 24 h. Purification by chromatography on silica gel (EtOAc/petroleum ether = 1:10) afforded the title product in 33% yield (11.8 mg). Yellow oil. ^{1}H NMR (400 MHz, $CDCl_3$) δ 7.96 – 7.91 (m, 2H), 7.56 – 7.51 (m, 1H), 7.47 – 7.41 (m, 2H), 2.86 (s, 2H), 1.07 (s, 9H). ^{13}C NMR (101 MHz, $CDCl_3$) δ 200.49, 138.57, 132.72, 128.49, 128.23, 50.06, 31.43, 30.10. The characterization data matched those previously reported.[6]

Cyclopentyl(phenyl)methanone (3f) (Scheme 1)

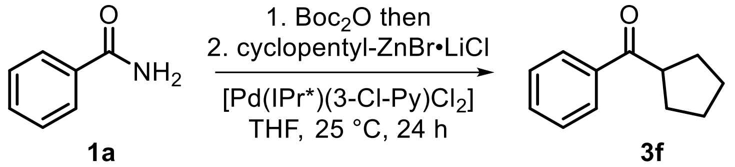

According to the general procedure, the reaction of *N*,*N*-Boc_2-benzamide (1.0 equiv), [Pd(IPr*)(3-Cl-Py)Cl_2] (10 mol%), and cyclopentyl-ZnBr·LiCl (1.5 equiv, 0.41 M in THF) was stirred at 25 °C for 24 h. Purification by chromatography on silica gel (EtOAc/petroleum ether = 1:10) afforded the title product in 90% yield (31.3 mg). Yellow oil. ^{1}H NMR (400 MHz, $CDCl_3$) δ 7.97 (d, J = 7.0 Hz, 2H), 7.53 (t, J = 7.3 Hz, 1H), 7.44 (t, J = 7.5 Hz, 2H), 3.71 (p, J = 7.9 Hz, 1H), 1.96 – 1.85 (m, 4H), 1.81 – 1.56 (m, 4H). ^{13}C NMR (126 MHz, $CDCl_3$) δ 202.82, 136.97, 132.70, 128.51, 128.47, 46.38, 29.99, 26.32. The characterization data matched those previously reported.[1]

Cyclohexyl(phenyl)methanone (3g) (Scheme 1)

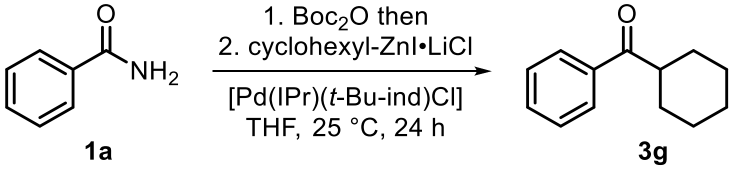

According to the general procedure, the reaction of *N*,*N*-Boc_2-benzamide (1.0 equiv), [Pd(IPr)(*t*-Bu-ind)Cl] (20 mol%), and cyclohexyl-ZnI·LiCl (1.5 equiv, 0.20 M in THF) was stirred at 25 °C for 24 h. Purification by chromatography on silica gel (EtOAc/petroleum ether = 1:10) afforded the title product in 63% yield (23.8 mg). Yellow oil. ^{1}H NMR (400 MHz, $CDCl_3$) δ 7.94 (d, J =

7.3 Hz, 2H), 7.58 – 7.49 (m, 1H), 7.44 (t, *J* = 7.5 Hz, 2H), 3.26 (tt, *J* = 11.5, 3.5 Hz, 1H), 1.92 – 1.80 (m, 4H), 1.73 (d, *J* = 12.4 Hz, 1H), 1.55 – 1.44 (m, 2H), 1.44 – 1.33 (m, 2H), 1.32 – 1.23 (m, 1H). ^{13}C NMR (101 MHz, $CDCl_3$) δ 203.85, 136.32, 132.74, 128.59, 128.26, 45.60, 29.44, 25.99, 25.87. The characterization data matched those previously reported.[1]

1-(Naphthalen-2-yl)butan-1-one (3h) (Scheme 1)

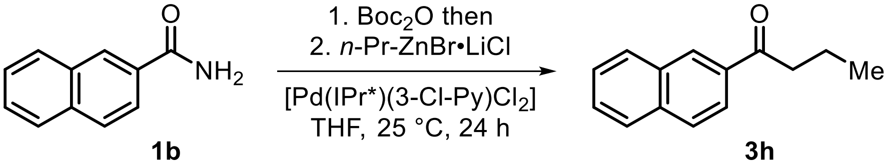

According to the general procedure, the reaction of *N*,*N*-Boc$_2$-2-naphthamide (1.0 equiv), [Pd(IPr*)(3-Cl-Py)Cl_2] (10 mol%), and *n*-Pr-ZnBr·LiCl (1.5 equiv, 0.50 M in THF) was stirred at 25 °C for 24 h. Purification by chromatography on silica gel (EtOAc/petroleum ether = 1:10) afforded the title product in 98% yield (38.7 mg). White solid. ^{1}H NMR (400 MHz, $CDCl_3$) δ 8.41 (s, 1H), 8.00 (dd, *J* = 8.6, 1.8 Hz, 1H), 7.90 (d, *J* = 7.9 Hz, 1H), 7.86 – 7.78 (m, 2H), 7.58 – 7.50 (m, 1H), 7.49 (m, 1H), 3.02 (t, *J* = 7.3 Hz, 2H), 1.80 (h, *J* = 7.3 Hz, 2H), 1.02 (t, *J* = 7.4 Hz, 3H). ^{13}C NMR (101 MHz, $CDCl_3$) δ 200.31, 135.51, 134.42, 132.57, 129.62, 129.56, 128.39, 128.34, 127.78, 126.73, 123.94, 40.57, 17.94, 14.00. The characterization data matched those previously reported.[7]

2-Methyl-1-(naphthalen-2-yl)propan-1-one (3i) (Scheme 1)

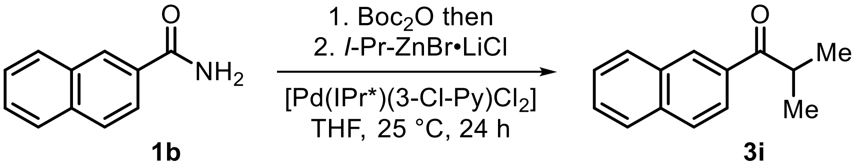

According to the general procedure, the reaction of *N*,*N*-Boc$_2$-2-naphthamide (1.0 equiv), [Pd(IPr*)(3-Cl-Py)Cl_2] (10 mol%), and *i*-Pr-ZnBr·LiCl (1.5 equiv, 0.34 M in THF) was stirred at 25 °C for 24 h. Purification by chromatography on silica gel (EtOAc/petroleum ether = 1:10) afforded the title product in 76% yield (30.0 mg). Yellow oil. ^{1}H NMR (400 MHz, $CDCl_3$) δ 8.47 (s, 1H), 8.03 (dd, *J* = 8.6, 1.8 Hz, 1H), 7.96 (d, *J* = 7.9 Hz, 1H), 7.90 (s, 1H), 7.89 – 7.83 (m, 1H), 7.61 – 7.56 (m, 1H), 7.56 – 7.52 (m, 1H), 3.73 (p, *J* = 6.8 Hz, 1H), 1.28 (d, *J* = 6.9 Hz, 6H). ^{13}C

NMR (101 MHz, $CDCl_3$) δ 204.55, 135.50, 133.52, 132.63, 129.74, 129.57, 128.48, 128.35, 127.77, 126.72, 124.37, 35.44, 19.35. The characterization data matched those previously reported.[1]

2-Methyl-1-(naphthalen-2-yl)butan-1-one (3j) (Scheme 1)

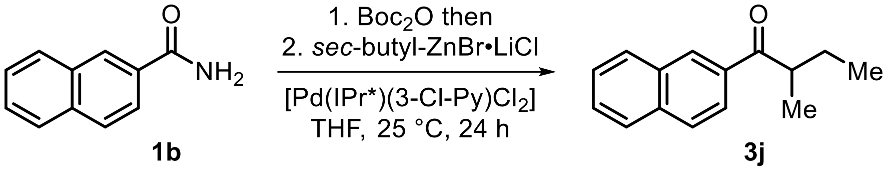

According to the general procedure, the reaction of *N*,*N*-Boc_2-2-naphthamide (1.0 equiv), [Pd(IPr*)(3-Cl-Py)Cl_2] (10 mol%), and *sec*-butyl-ZnBr·LiCl (1.5 equiv, 0.28 M in THF) was stirred at 25 °C for 24 h. Purification by chromatography on silica gel (EtOAc/petroleum ether = 1:10) afforded the title product in 64% yield (27.2 mg). Yellow oil. ^{1}H NMR (400 MHz, $CDCl_3$) δ 8.46 (s, 1H), 8.03 (dd, *J* = 8.6, 1.8 Hz, 1H), 7.95 (d, *J* = 7.9 Hz, 1H), 7.89 (s, 1H), 7.87 – 7.82 (m, 1H), 7.60 – 7.55 (m, 1H), 7.55 – 7.50 (m, 1H), 3.56 (h, *J* = 6.7 Hz, 1H), 1.93 – 1.86 (m, 1H), 1.59 – 1.52 (m, 1H), 1.25 (d, *J* = 6.8 Hz, 3H), 0.95 (t, *J* = 7.5 Hz, 3H). ^{13}C NMR (101 MHz, $CDCl_3$) δ 204.49, 135.51, 134.16, 132.64, 129.67, 129.59, 128.49, 128.36, 127.77, 126.73, 124.30, 42.18, 26.87, 17.01, 11.88. The characterization data matched those previously reported.[1]

3,3-Dimethyl-1-(naphthalen-2-yl)butan-1-one (3k) (Scheme 1)

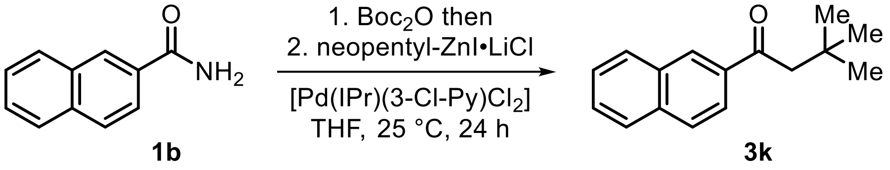

According to the general procedure, the reaction of *N*,*N*-Boc_2-2-naphthamide (1.0 equiv), [Pd(IPr)(3-Cl-Py)Cl_2] (20 mol%), and neopentyl-ZnI·LiCl (1.5 equiv, 0.50 M in THF) was stirred at 25 °C for 24 h. Purification by chromatography on silica gel (EtOAc/petroleum ether = 1:10) afforded the title product in 54% yield (24.3 mg). White solid. ^{1}H NMR (400 MHz, $CDCl_3$) δ 8.44 (s, 1H), 8.02 (d, *J* = 8.6 Hz, 1H), 7.96 (d, *J* = 7.9 Hz, 1H), 7.89 – 7.84 (m, 2H), 7.63 – 7.51 (m, 2H), 2.99 (s, 2H), 1.10 (s, 9H). ^{13}C NMR (101 MHz, $CDCl_3$) δ 200.46, 135.93, 135.42,

132.54, 129.88, 129.60, 128.37, 128.33, 127.75, 126.71, 124.15, 50.15, 31.60, 30.19. The characterization data matched those previously reported.[1]

Cyclopentyl(naphthalen-2-yl)methanone (3l) (Scheme 1)

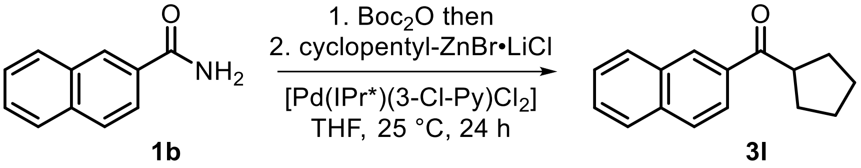

According to the general procedure, the reaction of *N,N*-Boc$_2$-2-naphthamide (1.0 equiv), [Pd(IPr*)(3-Cl-Py)Cl$_2$] (10 mol%), and cyclopentyl-ZnBr·LiCl (1.5 equiv, 0.41 M in THF) was stirred at 25 °C for 24 h. Purification by chromatography on silica gel (EtOAc/petroleum ether = 1:10) afforded the title product in 71% yield (31.7 mg). White solid. ^{1}H NMR (400 MHz, CDCl$_3$) δ 8.48 (s, 1H), 8.05 (dd, *J* = 8.6, 1.8 Hz, 1H), 7.96 (d, *J* = 7.9 Hz, 1H), 7.88 (t, *J* = 7.9 Hz, 2H), 7.61 – 7.57 (m, 1H), 7.56 – 7.51 (m, 1H), 3.88 (p, *J* = 7.9 Hz, 1H), 2.02 – 1.93 (m, 4H), 1.82 – 1.65 (m, 4H). ^{13}C NMR (101 MHz, CDCl$_3$) δ 202.86, 135.46, 134.28, 132.60, 129.98, 129.57, 128.34, 128.30, 127.76, 126.68, 124.46, 46.43, 30.17, 26.40. The characterization data matched those previously reported.[8]

Cyclohexyl(naphthalen-2-yl)methanone (3m) (Scheme 1)

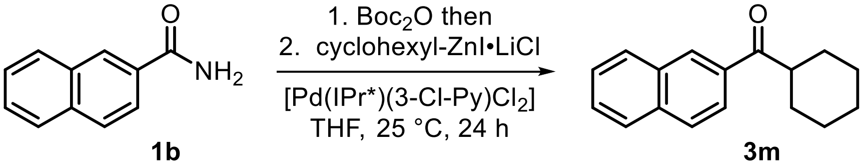

According to the general procedure, the reaction of *N,N*-Boc$_2$-2-naphthamide (1.0 equiv), [Pd(IPr*)(3-Cl-Py)Cl$_2$] (10 mol%), and cyclohexyl-ZnI·LiCl (1.5 equiv, 0.66 M in THF) was stirred at 25 °C for 24 h. Purification by chromatography on silica gel (EtOAc/petroleum ether = 1:10) afforded the title product in 50% yield (23.7 mg). White solid. ^{1}H NMR (400 MHz, CDCl$_3$) δ 8.44 (s, 1H), 8.00 (dd, *J* = 8.6, 1.8 Hz, 1H), 7.94 (d, *J* = 7.2 Hz, 1H), 7.89 – 7.80 (m, 2H), 7.60 – 7.47 (m, 2H), 3.47 – 3.35 (m, 1H), 1.94 (d, *J* = 13.1 Hz, 2H), 1.89 – 1.83 (m, 2H), 1.78 – 1.72 (m, 1H), 1.61 – 1.50 (m, 2H), 1.49 – 1.37 (m, 2H), 1.34 – 1.25 (m, 1H). ^{13}C NMR (126 MHz,

$CDCl_3$) δ 203.89, 135.48, 133.71, 132.64, 129.57, 129.55, 128.44, 128.27, 127.75, 126.67, 124.36, 45.73, 29.58, 26.01, 25.93. The characterization data matched those previously reported.[9]

2-Phenyl-1-(*p*-tolyl)ethan-1-one (3n) (Scheme 2)

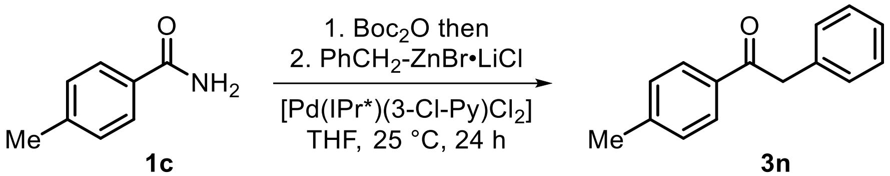

1c **3n**

According to the general procedure, the reaction of *N*,*N*-Boc$_2$-4-methylbenzamide (1.0 equiv), [Pd(IPr*)(3-Cl-Py)Cl_2] (10 mol%), and $PhCH_2$-ZnBr·LiCl (1.5 equiv, 0.625 M in THF) was stirred at 25 °C for 24 h. Purification by chromatography on silica gel (EtOAc/petroleum ether = 1:10) afforded the title product in 98% yield (41.1 mg). White solid. ^1H NMR (400 MHz, $CDCl_3$) δ 7.91 (d, *J* = 8.3 Hz, 2H), 7.34 – 7.29 (m, 2H), 7.28 – 7.22 (m, 5H), 4.26 (s, 2H), 2.39 (s, 3H). ^{13}C NMR (101 MHz, $CDCl_3$) δ 197.37, 144.05, 134.79, 134.10, 129.48, 129.37, 128.80, 128.68, 126.85, 45.45, 21.70. The characterization data matched those previously reported.[10]

1-(4-Methoxyphenyl)-2-phenylethan-1-one (3o) (Scheme 2)

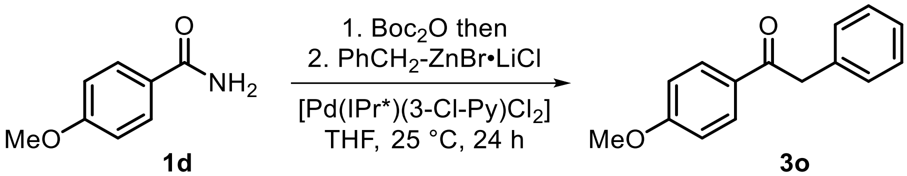

1d **3o**

According to the general procedure, the reaction of *N*,*N*-Boc$_2$-4-methoxybenzamide (1.0 equiv), [Pd(IPr*)(3-Cl-Py)Cl_2] (10 mol%), and $PhCH_2$-ZnBr·LiCl (1.5 equiv, 0.56 M in THF) was stirred at 25 °C for 24 h. Purification by chromatography on silica gel (EtOAc/petroleum ether = 1:10) afforded the title product in 98% yield (44.2 mg). Yellow solid. ^1H NMR (500 MHz, $CDCl_3$) δ 8.03 – 7.96 (m, 2H), 7.35 – 7.20 (m, 5H), 6.97 – 6.87 (m, 2H), 4.23 (s, 2H), 3.85 (s, 3H). ^{13}C NMR (126 MHz, $CDCl_3$) δ 196.24, 163.55, 135.01, 130.97, 129.68, 129.40, 128.65, 126.79, 113.81, 55.47, 45.29. The characterization data matched those previously reported.[1]

1-(4-Fluorophenyl)-2-phenylethan-1-one (3p) (Scheme 2)

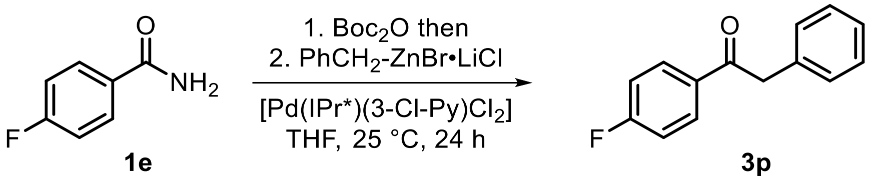

According to the general procedure, the reaction of *N*,*N*-Boc$_2$-4-fluorobenzamide (1.0 equiv), [Pd(IPr*)(3-Cl-Py)Cl$_2$] (10 mol%), and PhCH$_2$-ZnBr·LiCl (1.5 equiv, 0.625 M in THF) was stirred at 25 °C for 24 h. Purification by chromatography on silica gel (EtOAc/petroleum ether = 1:10) afforded the title product in 92% yield (39.4 mg). White solid. ^{1}H NMR (400 MHz, CDCl$_3$) δ 8.08 – 7.98 (m, 2H), 7.36 – 7.30 (m, 2H), 7.28 – 7.24 (m, 3H), 7.12 (t, *J* = 8.6 Hz, 2H), 4.26 (s, 2H). ^{13}C NMR (101 MHz, CDCl$_3$) δ 196.10, 165.77 (d, *J* = 255.0 Hz), 134.34, 132.95 (d, *J* = 3.0 Hz), 131.31 (d, *J* = 9.4 Hz), 129.41, 128.78, 127.03, 115.80 (d, *J* = 21.8 Hz), 45.53. ^{19}F NMR (376 MHz, CDCl$_3$) δ -104.95. The characterization data matched those previously reported.[1]

2-Phenyl-1-(4-(trifluoromethyl)phenyl)ethan-1-one (3q) (Scheme 2)

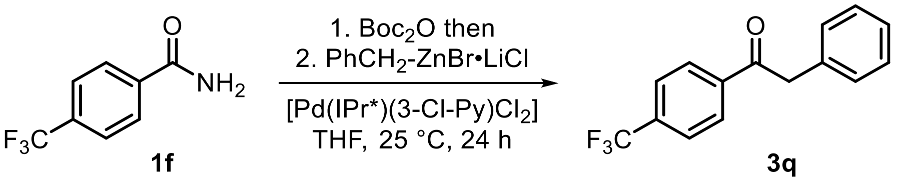

According to the general procedure, the reaction of *N*,*N*-Boc$_2$-4-(trifluoromethyl)benzamide (1.0 equiv), [Pd(IPr*)(3-Cl-Py)Cl$_2$] (10 mol%), and PhCH$_2$-ZnBr·LiCl (1.5 equiv, 0.625 M in THF) was stirred at 25 °C for 24 h. Purification by chromatography on silica gel (EtOAc/petroleum ether = 1:10) afforded the title product in 55% yield (29.2 mg). White solid. ^{1}H NMR (400 MHz, CDCl$_3$) δ 8.11 (d, *J* = 8.1 Hz, 2H), 7.72 (d, *J* = 8.0 Hz, 2H), 7.37 – 7.32 (m, 2H), 7.30 – 7.25 (m, 3H), 4.31 (s, 2H). ^{13}C NMR (101 MHz, CDCl$_3$) δ 196.65, 139.18, 134.45 (q, *J* = 31.8 Hz), 133.78, 129.40, 128.96, 128.87, 127.21, 125.75 (q, *J* = 3.7 Hz), 123.55 (q, *J* = 270.7 Hz), 45.85. ^{19}F NMR (376 MHz, CDCl$_3$) δ -63.14. The characterization data matched those previously reported.[1]

1-([1,1'-Biphenyl]-4-yl)-2-phenylethan-1-one (3r) (Scheme 2)

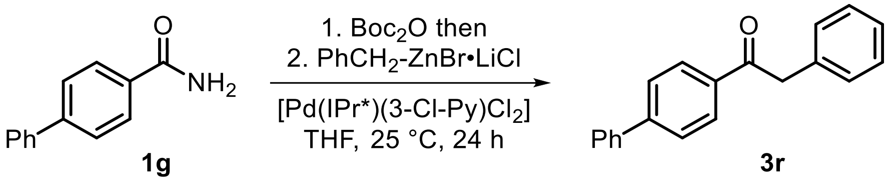

According to the general procedure, the reaction of *N*,*N*-Boc_2-[1,1'-biphenyl]-4-carboxamide (1.0 equiv), [Pd(IPr*)(3-Cl-Py)Cl_2] (10 mol%), and $PhCH_2$-ZnBr·LiCl (1.5 equiv, 0.71 M in THF) was stirred at 25 °C for 24 h. Purification by chromatography on silica gel (EtOAc/petroleum ether = 1:10) afforded the title product in 56% yield (30.7 mg). White solid. ^{1}H NMR (400 MHz, $CDCl_3$) δ 8.12 – 8.06 (m, 2H), 7.71 – 7.64 (m, 2H), 7.65 – 7.58 (m, 2H), 7.47 (t, *J* = 7.4 Hz, 2H), 7.44 – 7.24 (m, 6H), 4.32 (s, 2H). ^{13}C NMR (101 MHz, $CDCl_3$) δ 197.25, 145.86, 139.84, 135.27, 134.62, 129.48, 129.27, 128.99, 128.74, 128.29, 127.32, 127.29, 126.94, 45.61. The characterization data matched those previously reported.[11]

1-(4-Phenoxyphenyl)-2-phenylethan-1-one (3s) (Scheme 2)

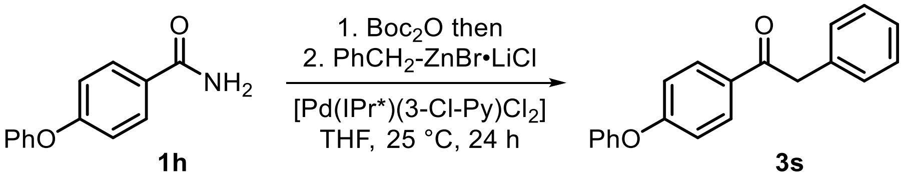

According to the general procedure, the reaction of *N*,*N*-Boc_2-4-phenoxybenzamide (1.0 equiv), [Pd(IPr*)(3-Cl-Py)Cl_2] (10 mol%), and $PhCH_2$-ZnBr·LiCl (1.5 equiv, 0.625 M in THF) was stirred at 25 °C for 24 h. Purification by chromatography on silica gel (EtOAc/petroleum ether = 1:10) afforded the title product in 80% yield (46.0 mg). White solid. ^{1}H NMR (400 MHz, $CDCl_3$) δ 7.99 (d, *J* = 8.9 Hz, 2H), 7.41 – 7.37 (m, 2H), 7.35 – 7.30 (m, 2H), 7.26 (d, *J* = 7.9 Hz, 3H), 7.20 (t, *J* = 7.5 Hz, 1H), 7.06 (d, *J* = 7.7 Hz, 2H), 6.98 (d, *J* = 8.8 Hz, 2H), 4.24 (s, 2H). ^{13}C NMR (101 MHz, $CDCl_3$) δ 196.25, 162.10, 155.38, 134.76, 131.17, 130.98, 130.09, 129.41, 128.72, 126.89, 124.72, 120.29, 117.28, 45.43. The characterization data matched those previously reported.[12]

2-Phenyl-1-(*o*-tolyl)ethan-1-one (3t) (Scheme 2)

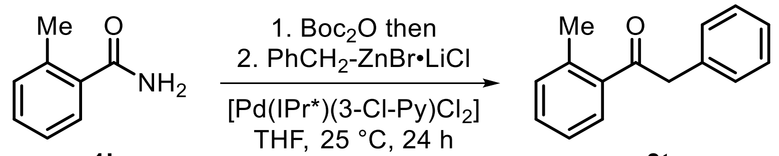

1i **3t**

According to the general procedure, the reaction of *N*,*N*-Boc_2-2-methylbenzamide (1.0 equiv), [Pd(IPr*)(3-Cl-Py)Cl_2] (10 mol%), and $PhCH_2$-ZnBr·LiCl (1.5 equiv, 0.625 M in THF) was stirred at 25 °C for 24 h. Purification by chromatography on silica gel (EtOAc/petroleum ether = 1:10) afforded the title product in 57% yield (24.1 mg). White solid. ^1H NMR (400 MHz, $CDCl_3$) δ 7.72 (d, *J* = 7.7 Hz, 1H), 7.39 – 7.30 (m, 3H), 7.27 – 7.21 (m, 5H), 4.21 (s, 2H), 2.44 (s, 3H). ^{13}C NMR (101 MHz, $CDCl_3$) δ 201.50, 138.61, 137.57, 134.47, 132.02, 131.39, 129.57, 128.68, 128.66, 126.91, 125.65, 48.44, 21.35. The characterization data matched those previously reported.[11]

2-Phenyl-1-(*m*-tolyl)ethan-1-one (3u) (Scheme 2)

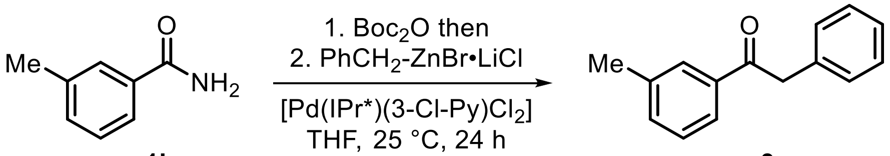

1j **3u**

According to the general procedure, the reaction of *N*,*N*-Boc_2-3-methylbenzamide (1.0 equiv), [Pd(IPr*)(3-Cl-Py)Cl_2] (10 mol%), and $PhCH_2$-ZnBr·LiCl (1.5 equiv, 0.625 M in THF) was stirred at 25 °C for 24 h. Purification by chromatography on silica gel (EtOAc/petroleum ether = 1:10) afforded the title product in 91% yield (38.2 mg). Yellow oil. ^1H NMR (400 MHz, $CDCl_3$) δ 7.84 – 7.78 (m, 2H), 7.37 – 7.30 (m, 4H), 7.28 – 7.24 (m, 3H), 4.27 (s, 2H), 2.40 (s, 3H). ^{13}C NMR (101 MHz, $CDCl_3$) δ 197.91, 138.50, 136.65, 134.67, 134.00, 129.52, 129.12, 128.69, 128.54, 126.89, 125.91, 45.54, 21.43. The characterization data matched those previously reported.[13]

1-(2-Methoxyphenyl)-2-phenylethan-1-one (3v) (Scheme 2)

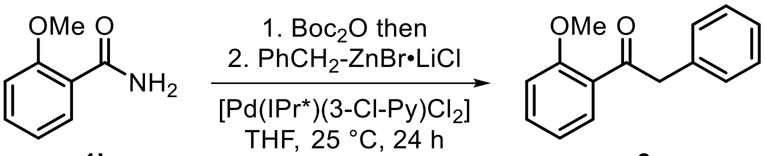

1k **3v**

According to the general procedure, the reaction of *N*,*N*-Boc$_2$-2-methoxybenzamide (1.0 equiv), [Pd(IPr*)(3-Cl-Py)Cl$_2$] (10 mol%), and PhCH$_2$-ZnBr·LiCl (1.5 equiv, 0.625 M in THF) was stirred at 25 °C for 24 h. Purification by chromatography on silica gel (EtOAc/petroleum ether = 1:10) afforded the title product in 56% yield (25.5 mg). White solid. ^{1}H NMR (500 MHz, CDCl$_3$) δ 7.72 – 7.66 (m, 1H), 7.52 – 7.44 (m, 1H), 7.34 – 7.30 (m, 2H), 7.27 – 7.23 (m, 3H), 7.04 – 6.94 (m, 2H), 4.33 (s, 2H), 3.94 (s, 3H). ^{13}C NMR (126 MHz, CDCl$_3$) δ 200.17, 158.40, 135.25, 133.50, 130.66, 129.69, 128.34, 126.58, 120.74, 111.51, 55.46, 50.17. The characterization data matched those previously reported.[14]

1-(3-Methoxyphenyl)-2-phenylethan-1-one (3w) (Scheme 2)

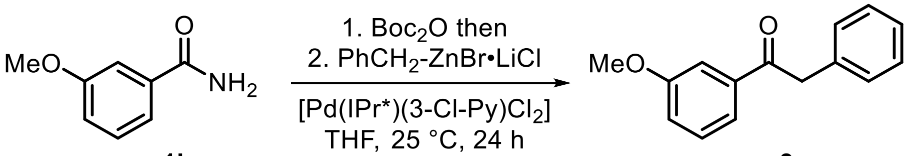

1l **3w**

According to the general procedure, the reaction of *N*,*N*-Boc$_2$-3-methoxybenzamide (1.0 equiv), [Pd(IPr*)(3-Cl-Py)Cl$_2$] (10 mol%), and PhCH$_2$-ZnBr·LiCl (1.5 equiv, 0.56 M in THF) was stirred at 25 °C for 24 h. Purification by chromatography on silica gel (EtOAc/petroleum ether = 1:10) afforded the title product in 87% yield (39.3 mg). Yellow solid. ^{1}H NMR (500 MHz, CDCl$_3$) δ 7.60 (d, J = 7.7 Hz, 1H), 7.52 (s, 1H), 7.38 – 7.30 (m, 3H), 7.28 – 7.21 (m, 3H), 7.12 – 7.06 (m, 1H), 4.26 (s, 2H), 3.83 (s, 3H). ^{13}C NMR (126 MHz, CDCl$_3$) δ 197.47, 159.88, 138.00, 134.59, 129.62, 129.45, 128.69, 126.91, 121.30, 119.67, 112.88, 55.43, 45.64. The characterization data matched those previously reported.[10]

1-(2-Fluorophenyl)-2-phenylethan-1-one (3x) (Scheme 2)

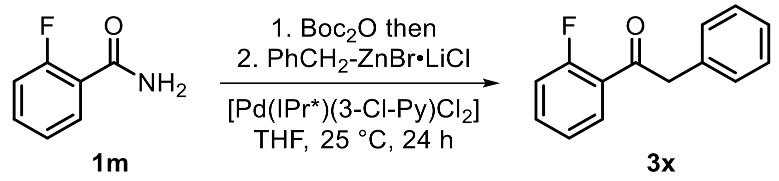

According to the general procedure, the reaction of *N,N*-Boc$_2$-2-fluorobenzamide (1.0 equiv), [Pd(IPr*)(3-Cl-Py)Cl$_2$] (20 mol%), and PhCH$_2$-ZnBr·LiCl (3.0 equiv, 0.71 M in THF) was stirred at 25 °C for 24 h. Purification by chromatography on silica gel (EtOAc/petroleum ether = 1:10) afforded the title product in 74% yield (31.8 mg). White solid. ^{1}H NMR (400 MHz, CDCl$_3$) δ 7.83 (td, *J* = 7.6, 1.9 Hz, 1H), 7.46 (tdd, *J* = 7.4, 4.9, 1.8 Hz, 1H), 7.30 (dd, *J* = 8.6, 6.3 Hz, 2H), 7.23 (d, *J* = 6.8 Hz, 3H), 7.17 (t, *J* = 7.6 Hz, 1H), 7.09 (dd, *J* = 11.3, 8.3 Hz, 1H), 4.27 (d, *J* = 2.6 Hz, 2H). ^{13}C NMR (101 MHz, CDCl$_3$) δ 196.18 (d, *J* = 4.4 Hz), 161.79 (d, *J* = 254.3 Hz), 134.71 (d, *J* = 9.0 Hz), 134.09 (d, *J* = 1.3 Hz), 131.07 (d, *J* = 2.7 Hz), 129.77, 128.58, 127.01, 125.59 (d, *J* = 13.1 Hz), 124.59 (d, *J* = 3.4 Hz), 116.72 (d, *J* = 23.9 Hz), 49.94 (d, *J* = 7.5 Hz). ^{19}F NMR (376 MHz, CDCl$_3$) δ -108.92. The characterization data matched those previously reported.[14]

1-(3-Fluorophenyl)-2-phenylethan-1-one (3y) (Scheme 2)

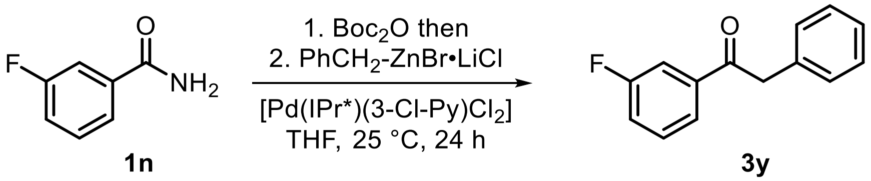

According to the general procedure, the reaction of *N,N*-Boc$_2$-3-fluorobenzamide (1.0 equiv), [Pd(IPr*)(3-Cl-Py)Cl$_2$] (20 mol%), and PhCH$_2$-ZnBr·LiCl (3.0 equiv, 0.71 M in THF) was stirred at 25 °C for 24 h. Purification by chromatography on silica gel (EtOAc/petroleum ether = 1:10) afforded the title product in 62% yield (26.5 mg). White solid. ^{1}H NMR (400 MHz, CDCl$_3$) δ 7.77 (d, *J* = 8.0 Hz, 1H), 7.67 (d, *J* = 9.5 Hz, 1H), 7.43 – 7.37 (m, 1H), 7.34 – 7.29 (m, 2H), 7.27 – 7.19 (m, 4H), 4.24 (s, 2H). ^{13}C NMR (101 MHz, CDCl$_3$) δ 196.39 (d, *J* = 2.1 Hz), 162.88 (d, *J* = 247.9 Hz), 138.68 (d, *J* = 6.1 Hz), 134.11, 130.39 (d, *J* = 7.6 Hz), 129.51, 128.82, 127.12, 124.44 (d, *J* = 3.0 Hz), 120.25 (d, *J* = 21.4 Hz), 115.33 (d, *J* = 22.2 Hz), 45.66. ^{19}F NMR (376 MHz, CDCl$_3$) δ -111.58. The characterization data matched those previously reported.[10]

2-Phenyl-1-(3-(trifluoromethyl)phenyl)ethan-1-one (3z) (Scheme 2)

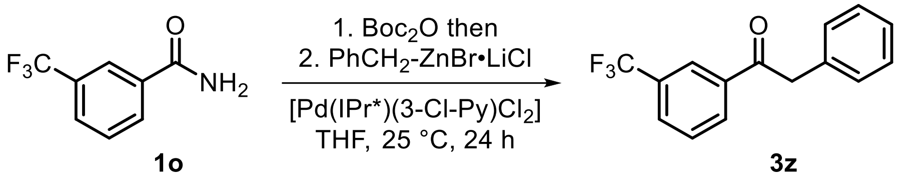

According to the general procedure, the reaction of *N*,*N*-Boc_2-3-(trifluoromethyl)benzamide (1.0 equiv), [Pd(IPr*)(3-Cl-Py)Cl_2] (10 mol%), and $PhCH_2$-ZnBr·LiCl (1.5 equiv, 0.625 M in THF) was stirred at 25 °C for 24 h. Purification by chromatography on silica gel (EtOAc/petroleum ether = 1:10) afforded the title product in 60% yield (31.6 mg). White solid. ^{1}H NMR (500 MHz, $CDCl_3$) δ 8.29 (s, 1H), 8.20 (d, *J* = 8.1 Hz, 1H), 7.83 (d, *J* = 7.8 Hz, 1H), 7.62 (t, *J* = 7.8 Hz, 1H), 7.40 – 7.34 (m, 2H), 7.29 (d, *J* = 7.4 Hz, 3H), 4.34 (s, 2H). ^{13}C NMR (101 MHz, $CDCl_3$) δ 196.26, 137.01, 133.77, 131.78, 131.31 (q, *J* = 32.9 Hz), 129.60 (q, *J* = 3.7 Hz), 129.45, 129.40 (q, *J* = 253.0 Hz), 129.38, 128.86, 127.21, 125.45 (q, *J* = 3.9 Hz), 45.66. ^{19}F NMR (376 MHz, $CDCl_3$) δ -62.81. The characterization data matched those previously reported.[10]

1-(3,4-Difluorophenyl)-2-phenylethan-1-one (3aa) (Scheme 2)

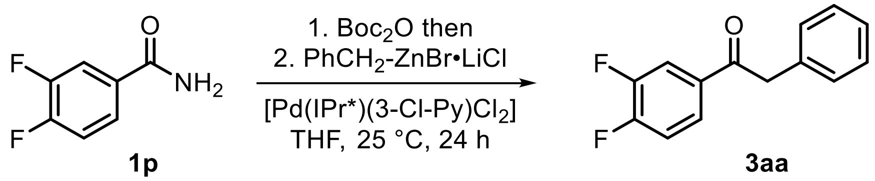

According to the general procedure, the reaction of *N*,*N*-Boc_2-3,4-difluorobenzamide (1.0 equiv), [Pd(IPr*)(3-Cl-Py)Cl_2] (10 mol%), and $PhCH_2$-ZnBr·LiCl (1.5 equiv, 0.625 M in THF) was stirred at 25 °C for 24 h. Purification by chromatography on silica gel (EtOAc/petroleum ether = 1:10) afforded the title product in 66% yield (30.5 mg). White solid. ^{1}H NMR (400 MHz, $CDCl_3$) δ 7.88 – 7.74 (m, 2H), 7.38 – 7.31 (m, 2H), 7.30 – 7.22 (m, 4H), 4.24 (s, 2H). ^{13}C NMR (101 MHz, $CDCl_3$) δ 195.08, 153.65 (dd, *J* = 257.4, 13.0 Hz), 150.44 (dd, *J* = 251.0, 13.1 Hz), 133.87, 133.61 (t, *J* = 3.7 Hz), 129.35, 128.86, 127.19, 125.67 (dd, *J* = 7.5, 3.7 Hz), 117.92 (dd, *J* = 18.1, 1.8 Hz), 117.56 (d, *J* = 17.9 Hz), 45.50. ^{19}F NMR (376 MHz, $CDCl_3$) δ -129.48 (d, *J* = 20.8 Hz), -135.81 (d, *J* = 20.9 Hz). The characterization data matched those previously reported.[15]

1-(3,4-Dimethoxyphenyl)-2-phenylethan-1-one (3ab) (Scheme 2)

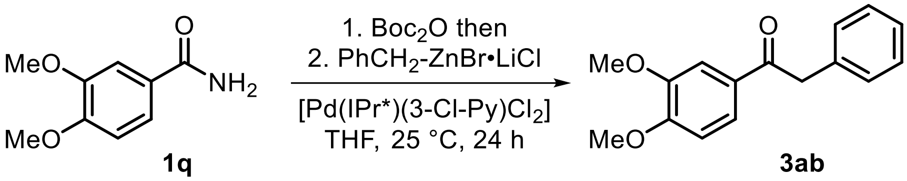

According to the general procedure, the reaction of *N*,*N*-Boc2-3,4-dimethoxybenzamide (1.0 equiv), [Pd(IPr*)(3-Cl-Py)Cl2] (10 mol%), and PhCH2-ZnBr·LiCl (1.5 equiv, 0.625 M in THF) was stirred at 25 °C for 24 h. Purification by chromatography on silica gel (EtOAc/petroleum ether = 1:10) afforded the title product in 71% yield (36.3 mg). White solid. ^{1}H NMR (400 MHz, CDCl3) δ 7.35 – 7.30 (m, 2H), 7.28 – 7.24 (m, 3H), 7.14 (d, *J* = 2.3 Hz, 2H), 6.64 (t, *J* = 2.3 Hz, 1H), 4.24 (s, 2H), 3.81 (s, 6H). ^{13}C NMR (101 MHz, CDCl3) δ 197.34, 160.87, 138.52, 134.57, 129.43, 128.71, 126.93, 106.49, 105.40, 55.59, 45.67. The characterization data matched those previously reported.[16]

1-(Benzo[*d*][1,3]dioxol-5-yl)-2-phenylethan-1-one (3ac) (Scheme 2)

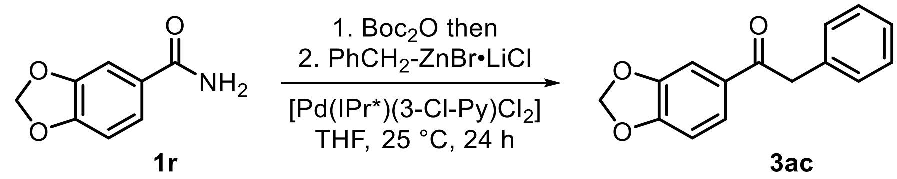

According to the general procedure, the reaction of *N*,*N*-Boc2-benzo[*d*][1,3]dioxole-5-carboxamide (1.0 equiv), [Pd(IPr*)(3-Cl-Py)Cl2] (20 mol%), and PhCH2-ZnBr·LiCl (3.0 equiv, 0.625 M in THF) was stirred at 25 °C for 24 h. Purification by chromatography on silica gel (EtOAc/petroleum ether = 1:10) afforded the title product in 61% yield (29.5 mg). Yellow solid. ^{1}H NMR (400 MHz, CDCl3) δ 7.62 (d, *J* = 8.4 Hz, 1H), 7.47 (s, 1H), 7.34 – 7.29 (m, 2H), 7.28 – 7.23 (m, 3H), 6.83 (d, *J* = 8.2 Hz, 1H), 6.02 (s, 2H), 4.20 (s, 2H). ^{13}C NMR (101 MHz, CDCl3) δ 195.78, 151.85, 148.25, 134.83, 131.41, 129.38, 128.71, 126.88, 125.06, 108.39, 107.93, 101.90, 45.37. The characterization data matched those previously reported.[11]

2-Phenyl-1-(thiophen-3-yl)ethan-1-one (3ad) (Scheme 2)

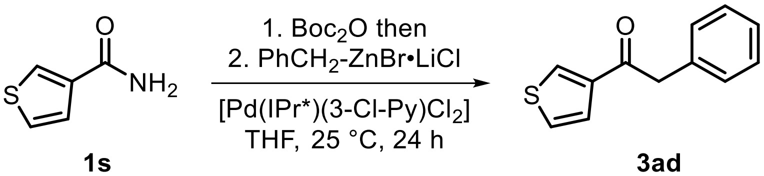

1s **3ad**

According to the general procedure, the reaction of *N*,*N*-Boc_2-thiophene-3-carboxamide (1.0 equiv), [Pd(IPr*)(3-Cl-Py)Cl_2] (10 mol%), and $PhCH_2$-ZnBr·LiCl (1.5 equiv, 0.71 M in THF) was stirred at 25 °C for 24 h. Purification by chromatography on silica gel (EtOAc/petroleum ether = 1:10) afforded the title product in 55% yield (22.4 mg). Yellow oil. ^{1}H NMR (400 MHz, $CDCl_3$) δ 8.09 (dd, *J* = 2.9, 1.3 Hz, 1H), 7.56 (dd, *J* = 5.1, 1.2 Hz, 1H), 7.37 – 7.21 (m, 6H), 4.17 (s, 2H). ^{13}C NMR (101 MHz, $CDCl_3$) δ 191.92, 141.86, 134.47, 132.74, 129.44, 128.74, 127.36, 127.00, 126.44, 46.94. The characterization data matched those previously reported.[10]

2-Phenyl-1-(thiophen-2-yl)ethan-1-one (3ae) (Scheme 2)

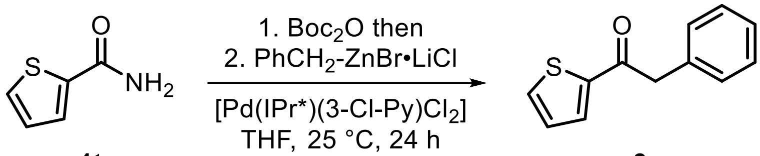

1t **3ae**

According to the general procedure, the reaction of *N*,*N*-Boc_2-thiophene-2-carboxamide (1.0 equiv), [Pd(IPr*)(3-Cl-Py)Cl_2] (10 mol%), and $PhCH_2$-ZnBr·LiCl (1.5 equiv, 0.71 M in THF) was stirred at 25 °C for 24 h. Purification by chromatography on silica gel (EtOAc/petroleum ether = 1:10) afforded the title product in 47% yield (19.2 mg). Yellow solid. ^{1}H NMR (400 MHz, $CDCl_3$) δ 7.77 (dd, *J* = 3.9, 1.1 Hz, 1H), 7.64 (dd, *J* = 4.9, 1.1 Hz, 1H), 7.37 – 7.29 (m, 4H), 7.28 – 7.23 (m, 1H), 7.17 – 7.09 (m, 1H), 4.20 (s, 2H). ^{13}C NMR (101 MHz, $CDCl_3$) δ 190.50, 143.90, 134.34, 134.09, 132.69, 129.42, 128.73, 128.21, 127.08, 46.41. The characterization data matched those previously reported.[17]

1-(Furan-2-yl)-2-phenylethan-1-one (3af) (Scheme 2)

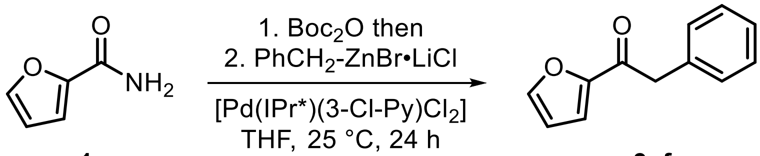

1u **3af**

According to the general procedure, the reaction of *N*,*N*-Boc$_2$-furan-2-carboxamide (1.0 equiv), [Pd(IPr*)(3-Cl-Py)Cl$_2$] (10 mol%), and PhCH$_2$-ZnBr·LiCl (3.0 equiv, 0.625 M in THF) was stirred at 25 °C for 24 h. Purification by chromatography on silica gel (EtOAc/petroleum ether = 1:10) afforded the title product in 61% yield (22.9 mg). Yellow oil. ^{1}H NMR (400 MHz, CDCl$_3$) δ 7.60 (d, *J* = 1.6 Hz, 1H), 7.35 – 7.28 (m, 4H), 7.27 (d, *J* = 2.4 Hz, 1H), 7.22 (d, *J* = 3.6 Hz, 1H), 6.53 (dd, *J* = 3.6, 1.7 Hz, 1H), 4.12 (s, 2H). ^{13}C NMR (126 MHz, CDCl$_3$) δ 186.62, 146.57, 134.09, 129.52, 128.66, 127.02, 117.87, 112.41, 45.44. The characterization data matched those previously reported.[14]

1-(Naphthalen-2-yl)-2-phenylethan-1-one (3ag) (Scheme 2)

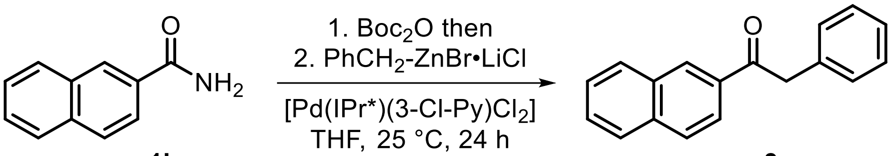

1b 3ag

According to the general procedure, the reaction of *N*,*N*-Boc$_2$-2-naphthamide (1.0 equiv), [Pd(IPr*)(3-Cl-Py)Cl$_2$] (10 mol%), and PhCH$_2$-ZnBr·LiCl (1.5 equiv, 0.56 M in THF) was stirred at 25 °C for 24 h. Purification by chromatography on silica gel (EtOAc/petroleum ether = 1:10) afforded the title product in 98% yield (48.1 mg). White solid. ^{1}H NMR (500 MHz, CDCl$_3$) δ 8.54 (s, 1H), 8.06 (d, *J* = 8.6 Hz, 1H), 7.95 (d, *J* = 8.1 Hz, 1H), 7.86 (t, *J* = 9.0 Hz, 2H), 7.64 – 7.50 (m, 2H), 7.36 – 7.27 (m, 4H), 7.27 – 7.22 (m, 1H), 4.40 (s, 2H). ^{13}C NMR (126 MHz, CDCl$_3$) δ 197.62, 135.63, 134.72, 134.01, 132.54, 130.42, 129.64, 129.52, 128.73, 128.57, 128.54, 127.80, 126.93, 126.82, 124.30, 45.58. The characterization data matched those previously reported.[1]

Scale-up Reaction

General Procedure. An oven-dried vial equipped with a stir bar was charged with 3-methylbenzamide (1.0 g, 7.4 mmol, 1.0 equiv), di-*tert*-butyl-dicarbonate (2.5 equiv), DMAP (15 mol%), and Et_3N (2.5 equiv). CH_2Cl_2 (0.25 M) was added with vigorous stirring and the resulting reaction mixture was stirred at room temperature for 15 h. After the indicated time, the reaction mixture was concentrated and purified directly by chromatography on silica gel to give analytically pure product. An oven-dried vial equipped with a stir bar was charged with the prepared *N*,*N*-Boc_2-3-methylbenzamide, [Pd(IPr*)(3-Cl-Py)Cl_2] (10 mol%), and $PhCH_2$-ZnBr·LiCl (0.50 M in THF, 1.5 equiv), placed under a positive pressure of argon, and subjected to three evacuation/backfilling cycles under high vacuum. The reaction mixture was stirred at 25 °C for 24 h. After the indicated time, the reaction mixture was diluted with CH_2Cl_2 (30 mL), filtered, and concentrated. The sample was analyzed by ^{1}H NMR ($CDCl_3$, 500 MHz) and GC-MS to obtain conversion, selectivity and yield using internal standard and comparison with authentic samples. Purification by chromatography on silica gel (EtOAc/petroleum ether = 1:10) afforded the title product. Yield 84% (1.3 g). Yellow oil. ^{1}H NMR (400 MHz, Chloroform-*d*) δ 7.84 – 7.79 (m, 2H), 7.38 – 7.30 (m, 4H), 7.28 – 7.22 (m, 3H), 4.27 (s, 2H), 2.40 (s, 3H). ^{13}C NMR (101 MHz, $CDCl_3$) δ 197.89, 138.48, 136.67, 134.67, 133.97, 129.50, 129.11, 128.67, 128.52, 126.87, 125.90, 45.53, 21.41. The characterization data matched those previously reported.[13]

Scheme S1. Scale-up Reaction^a

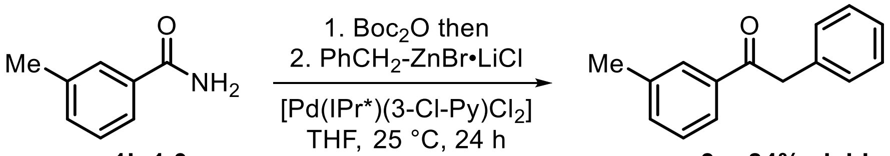

1j: 1.0 g **3u: 84% yield**

^{a}Conditions: 3-methylbenzamide (1.0 g, 7.4 mmol, 1.0 equiv), Boc_2O (2.5 equiv), DMAP (15 mol%), and Et_3N (2.5 equiv). DCM (0.25 M), rt, 15 h; [Pd] (10 mol%), $PhCH_2$-ZnBr·LiCl (0.50 M in THF, 1.5 equiv), 25 °C, 24 h.

Application of the Synthesis of Pharmaceutical Derivatives and Their Intermediates

General Procedure. An oven-dried vial equipped with a stir bar was charged with an amide substrate (neat, 1.0 equiv), [Pd(IPr*)(3-Cl-Py)Cl_2] (typically, 10 mol%), and Alkyl-ZnBr·LiCl (1.5 equiv, solution in THF), placed under a positive pressure of argon, and subjected to three evacuation/backfilling cycles under high vacuum. The reaction mixture was stirred at 25 °C for 24 h. After the indicated time, the reaction mixture was diluted with CH_2Cl_2 (10 mL), filtered, and concentrated. The sample was analyzed by ^{1}H NMR ($CDCl_3$, 400 MHz or 500 MHz) and GC-MS to obtain conversion, selectivity and yield using internal standard and comparison with authentic samples. Purification by chromatography on silica gel (EtOAc/petroleum ether = 1:10) afforded the title product.

Cyclopentyl(4-(trifluoromethyl)phenyl)methanone (3ah) (Scheme 3)

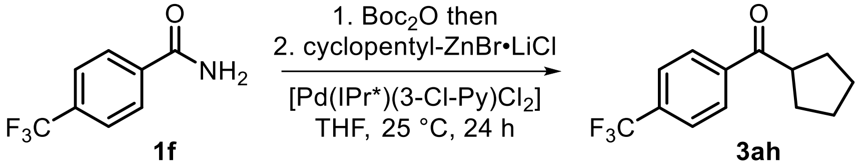

According to the general procedure, the reaction of *N*,*N*-Boc_2-4-(trifluoromethyl)benzamide (1.0 equiv), [Pd(IPr*)(3-Cl-Py)Cl_2] (20 mol%), and cyclopentyl-ZnBr·LiCl (3.0 equiv, 0.38 M in THF) was stirred at 25 °C for 24 h. Purification by chromatography on silica gel (EtOAc/petroleum ether = 1:10) afforded the title product in 81% yield (39.4 mg). Yellow oil. ^{1}H NMR (400 MHz, $CDCl_3$) δ 7.95 (d, *J* = 8.1 Hz, 2H), 7.59 (d, *J* = 8.2 Hz, 2H), 3.60 (p, *J* = 7.6 Hz, 1H), 1.80 (m, 4H), 1.68 – 1.48 (m, 4H). ^{13}C NMR (101 MHz, $CDCl_3$) δ 201.48, 139.59, 133.90 (q, *J* = 32.5 Hz), 128.70, 125.47 (q, *J* = 3.7 Hz), 123.65 (q, *J* = 272.6 Hz), 46.57, 29.70, 26.19. ^{19}F NMR (376 MHz, $CDCl_3$) δ -63.24. The characterization data matched those previously reported.[18]

Cyclopentyl(3,5-dimethoxyphenyl)methanone (3ai) (Scheme 3)

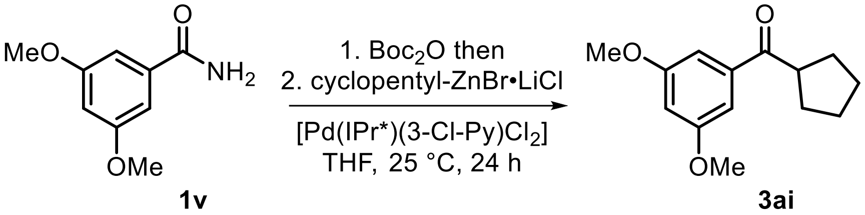

According to the general procedure, the reaction of *N*,*N*-Boc$_2$-3,5-dimethoxybenzamide (1.0 equiv), [Pd(IPr*)(3-Cl-Py)Cl$_2$] (20 mol%), and cyclopentyl-ZnBr·LiCl (3.0 equiv, 0.35 M in THF) was stirred at 25 °C for 24 h. Purification by chromatography on silica gel (EtOAc/petroleum ether = 1:10) afforded the title product in 56% yield (26.4 mg). Yellow oil. ^{1}H NMR (400 MHz, CDCl$_3$) δ 7.11 (d, *J* = 2.3 Hz, 2H), 6.64 (t, *J* = 2.3 Hz, 1H), 3.84 (s, 6H), 3.65 (p, *J* = 7.9 Hz, 1H), 1.96 – 1.84 (m, 4H), 1.79 – 1.60 (m, 4H). ^{13}C NMR (101 MHz, CDCl$_3$) δ 202.50, 160.80, 138.97, 106.32, 104.86, 55.58, 46.47, 30.11, 26.32. The characterization data matched those previously reported.[19]

Cyclopentyl(5,6,7,8-tetrahydronaphthalen-2-yl)methanone (3aj) (Scheme 3)

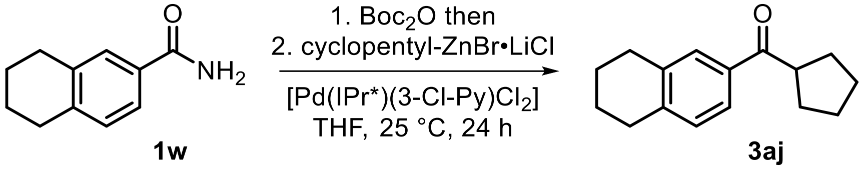

According to the general procedure, the reaction of *N*,*N*-Boc$_2$-5,6,7,8-tetrahydronaphthalene-2-carboxamide (1.0 equiv), [Pd(IPr*)(3-Cl-Py)Cl$_2$] (20 mol%), and cyclopentyl-ZnBr·LiCl (3.0 equiv, 0.38 M in THF) was stirred at 25 °C for 24 h. Purification by chromatography on silica gel (EtOAc/petroleum ether = 1:10) afforded the title product in 70% yield (31.8 mg). Yellow oil. ^{1}H NMR (400 MHz, CDCl$_3$) δ 7.68 (d, *J* = 6.2 Hz, 2H), 7.12 (d, *J* = 8.5 Hz, 1H), 3.68 (p, *J* = 7.9 Hz, 1H), 2.80 (d, *J* = 5.8 Hz, 4H), 1.94 – 1.86 (m, 4H), 1.80 (p, *J* = 3.3 Hz, 4H), 1.75 – 1.59 (m, 4H). ^{13}C NMR (101 MHz, CDCl$_3$) δ 202.89, 142.77, 137.35, 134.43, 129.39, 129.26, 125.59, 46.22, 30.10, 29.62, 29.45, 26.34, 23.02, 22.88. The characterization data matched those previously reported.[20]

1-(5,6,7,8-Tetrahydronaphthalen-2-yl)butan-1-one (3ak) (Scheme 3)

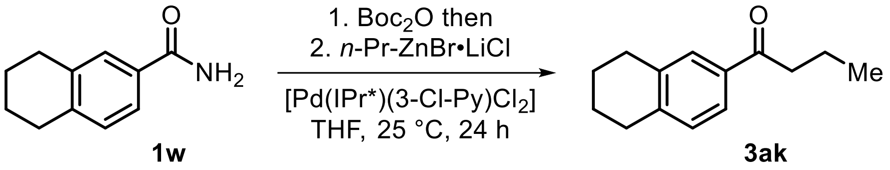

According to the general procedure, the reaction of *N*,*N*-Boc$_2$-5,6,7,8-tetrahydronaphthalene-2-carboxamide (1.0 equiv), [Pd(IPr*)(3-Cl-Py)Cl$_2$] (10 mol%), and *n*-Pr-ZnBr·LiCl (1.5 equiv, 0.50 M in THF) was stirred at 25 °C for 24 h. Purification by chromatography on silica gel (EtOAc/petroleum ether = 1:10) afforded the title product in 91% yield (36.8 mg). Yellow oil. ^{1}H NMR (400 MHz, $CDCl_3$) δ 7.59 (m, 2H), 7.05 (d, *J* = 8.4 Hz, 1H), 2.83 (t, *J* = 7.3 Hz, 2H), 2.73 (q, *J* = 5.1 Hz, 4H), 1.80 – 1.71 (m, 4H), 1.68 (q, *J* = 7.4 Hz, 2H), 0.92 (t, *J* = 7.4 Hz, 3H). ^{13}C NMR (101 MHz, $CDCl_3$) δ 200.52, 142.92, 137.40, 134.63, 129.30, 128.97, 125.18, 40.45, 29.63, 29.42, 23.01, 22.87, 17.98, 13.95. The characterization data matched those previously reported.[20]

1-(1-Methyl-1H-indol-5-yl)butan-1-one (3al) (Scheme 3)

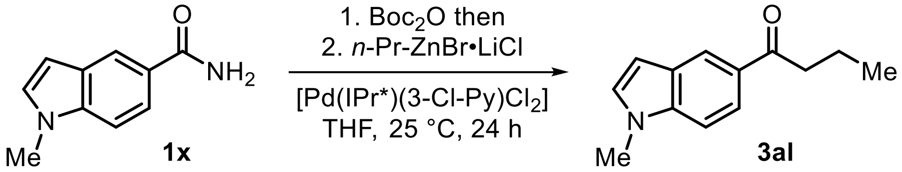

According to the general procedure, the reaction of *N*,*N*-Boc$_2$-1-methyl-1*H*-indole-5-carboxamide (1.0 equiv), [Pd(IPr*)(3-Cl-Py)Cl$_2$] (20 mol%), and *n*-Pr-ZnBr·LiCl (3.0 equiv, 0.45 M in THF) was stirred at 25 °C for 24 h. Purification by chromatography on silica gel (EtOAc/petroleum ether = 1:10) afforded the title product in 43% yield (17.3 mg). Yellow solid. ^{1}H NMR (400 MHz, $CDCl_3$) δ 8.31 (d, *J* = 1.7 Hz, 1H), 7.91 (dd, *J* = 8.7, 1.7 Hz, 1H), 7.34 (d, *J* = 8.7 Hz, 1H), 7.11 (d, *J* = 3.1 Hz, 1H), 6.60 (d, *J* = 3.1 Hz, 1H), 3.82 (s, 3H), 3.02 (t, *J* = 7.4 Hz, 2H), 1.81 (h, *J* = 7.4 Hz, 2H), 1.03 (t, *J* = 7.4 Hz, 3H). ^{13}C NMR (101 MHz, $CDCl_3$) δ 200.66, 139.07, 130.34, 129.34, 127.91, 122.78, 121.78, 109.09, 102.94, 40.49, 33.06, 28.05, 18.38, 14.08. The characterization data matched those previously reported.[21]

3-(4-Phenyl-5-(*m*-tolyl)oxazol-2-yl)propanoic acid (4u) (Scheme 3)

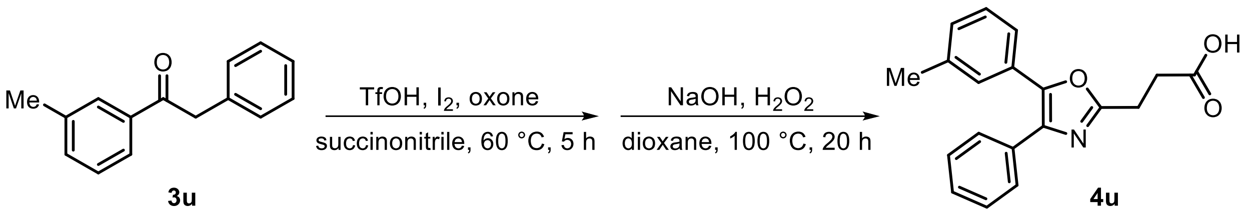

To a solution of 2-phenyl-1-(*m*-tolyl)ethan-1-one **3u** (210.3 mg, 1 mmol) in succinonitrile (6 mL), TfOH (600.3 mg, 0.35 mL, 4 mmol), iodine (177.7 mg, 0.7 mmol), and Oxone® (676.3 mg, 1.1 mmol) were added. The mixture was stirred at 60 °C for 5 h under argon. Upon completion of the reaction, the mixture was poured into a satd aq Na_2SO_3 and satd aq $NaHCO_3$ solution, followed by extraction with ethyl acetate (3×30 mL). The organic layer was dried over Na_2SO_4, filtered, and concentrated under reduced pressure. The residue was purified by short-flash column chromatography on silica gel (EtOAc /petroleum ether = 1:4) to afford 3-(4-phenyl-5-(*m*-tolyl)oxazol-2-yl)propanenitrile.

Subsequently, 3-(4-phenyl-5-(*m*-tolyl)oxazol-2-yl)propanenitrile (57.7 mg, 0.2 mmol) was dissolved in 1,4-dioxane (1 mL) and added to a mixture of 4 M NaOH (2 mL) and 30% aqueous H_2O_2 (1 mL). The mixture was stirred at 100 °C for 20 h under argon. After cooling to room temperature, the reaction mixture was diluted with 1 M HCl (20 mL) and extracted with ethyl acetate (3×30 mL). The organic layer was dried over Na_2SO_4, filtered, and concentrated under reduced pressure. Purification by short-flash column chromatography on silica gel (EtOAc /petroleum ether/acetic acid = 30:60:1) gave the title product in 63% yield (38.8 mg). White solid. ^{1}H NMR (400 MHz, $CDCl_3$) δ 7.62 (dd, *J* = 8.1, 1.6 Hz, 2H), 7.40 (s, 1H), 7.38 – 7.30 (m, 4H), 7.22 (t, *J* = 7.7 Hz, 1H), 7.13 (d, *J* = 7.5 Hz, 1H), 3.19 (t, *J* = 7.4 Hz, 2H), 2.95 (t, *J* = 7.4 Hz, 2H), 2.33 (s, 3H). ^{13}C NMR (101 MHz, $CDCl_3$) δ 176.66, 161.80, 145.77, 138.43, 134.73, 132.08, 129.43, 128.64, 128.56, 128.16, 127.94, 127.13, 123.71, 30.98, 23.25, 21.43. The characterization data matched those previously reported.[13]

(*E*)-3-Hydroxy-N'-(1-(5,6,7,8-tetrahydronaphthalen-2-yl)butylidene)-2-naphthohydrazide (4ak) (Scheme 3)

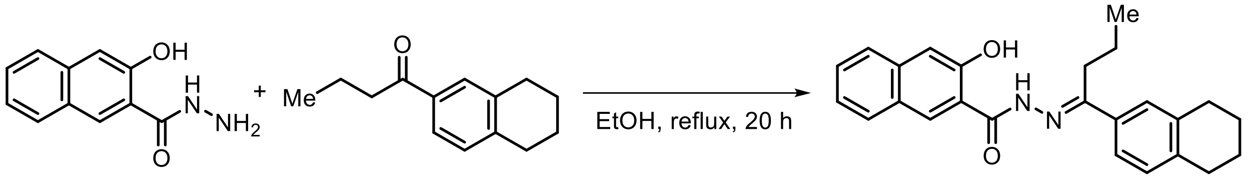

1z' **3ak** **4ak**

A mixture of 3-hydroxy-2-naphthohydrazide (40.4 mg, 0.2 mmol) and 1-(5,6,7,8-tetrahydronaphthalen-2-yl)butan-1-one (40.5 mg, 0.2 mmol) was refluxed in ethanol under a nitrogen atmosphere for 20 h. The reaction mixture was filtered and washed with warm ethanol to afford the title product in 73% yield (56.5 mg). White solid. ^{1}H NMR (400 MHz, DMSO-d_6) δ 11.82 (s, 1H), 11.69 (s, 1H), 8.66 (s, 1H), 8.01 (d, *J* = 8.2 Hz, 1H), 7.79 (d, *J* = 8.3 Hz, 1H), 7.59 (d, *J* = 8.3 Hz, 2H), 7.53 (t, *J* = 7.5 Hz, 1H), 7.38 (d, *J* = 7.3 Hz, 2H), 7.12 (t, *J* = 7.1 Hz, 1H), 2.81 – 2.74 (m, 6H), 1.76 (s, 4H), 1.59 (q, *J* = 7.8 Hz, 2H), 1.04 (t, *J* = 7.3 Hz, 3H). ^{13}C NMR (101 MHz, DMSO-d_6) δ 161.55, 155.76, 152.95, 138.80, 137.12, 136.22, 134.69, 133.03, 129.51, 129.45, 128.76, 127.76, 127.44, 126.20, 126.07, 124.41, 124.21, 121.28, 111.22, 29.38, 29.28, 29.14, 23.18, 23.10, 19.72, 14.63. In the ^{1}H NMR, each peak appears twice with an intensity ratio of approximately 5:1. This is due to the presence of two isomers, *E* and *Z*, in a ratio of 5:1. The characterization data matched those previously reported.[20]

Comparative Cross-Coupling of Different Acyl Precursors

General Procedure. An oven-dried vial equipped with a stir bar was charged with an acyl precursor (neat, 1.0 equiv), [Pd(IPr*)(3-Cl-Py)Cl_2] (typically, 10 mol%), and $PhCH_2$-ZnBr·LiCl (1.5 equiv, solution in THF), placed under a positive pressure of argon, and subjected to three evacuation/backfilling cycles under high vacuum. The reaction mixture was stirred at 25 °C for 24 h. After the indicated time, the reaction mixture was diluted with CH_2Cl_2 (10 mL), filtered, and concentrated. The sample was analyzed by ^{1}H NMR ($CDCl_3$, 400 MHz or 500 MHz) and GC-MS to obtain conversion, selectivity and yield using internal standard and comparison with authentic samples.

One-Pot Reaction

<u>*General Procedure.*</u> An oven-dried vial equipped with a stir bar was charged with benzamide (neat, 0.20 mmol, 1.0 equiv), di-tert-butyl-dicarbonate (2.5 equiv), DMAP (15 mol%), and Et_3N (2.5 equiv). CH_2Cl_2 (0.25 M) was added with vigorous stirring and the resulting reaction mixture was stirred at room temperature for 15 h. After the indicated time, the solvent was removed under reduced pressure, the reaction vial was charged with [Pd(IPr*)(3-Cl-Py)Cl_2] (10 mol%), and $PhCH_2$-ZnBr·LiCl (0.633 M in THF, 1.5 equiv), placed under a positive pressure of argon, and subjected to three evacuation/backfilling cycles under high vacuum. The reaction mixture was stirred at 25 °C for 24 h. After the indicated time, the reaction mixture was diluted with CH_2Cl_2 (10 mL), filtered, and concentrated. The sample was analyzed by ^{1}H NMR ($CDCl_3$, 400 MHz) and GC-MS to obtain conversion, selectivity and yield using internal standard and comparison with authentic samples. Purification by chromatography on silica gel (EtOAc/petroleum ether = 1:10) afforded the title product. Yield 84% (32.8 mg). White solid. ^{1}H NMR (400 MHz, $CDCl_3$) δ 8.04 – 7.95 (m, 2H), 7.57 – 7.50 (m, 1H), 7.43 (t, *J* = 7.6 Hz, 2H), 7.34 – 7.29 (m, 2H), 7.28 – 7.20 (m, 3H), 4.26 (s, 2H). ^{13}C NMR (101 MHz, $CDCl_3$) δ 197.70, 136.63, 134.61, 133.25, 129.55, 128.74, 128.72, 128.68, 126.96, 45.55. The characterization data matched those previously reported.[1]

Scheme S2. One-Pot Reaction*^{a}*

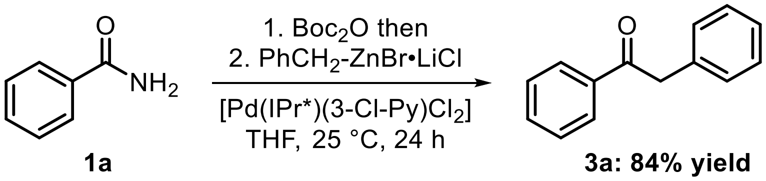

^{a}Conditions: benzamide (0.2 mmol, 1.0 equiv), Boc_2O (2.5 equiv), DMAP (15 mol%), Et_3N (2.5 equiv), DCM (0.25 M), rt, 15 h; [Pd] (10 mol%), $PhCH_2$-ZnBr·LiCl (0.63 M in THF, 1.5 equiv), 25 °C, 24 h.

References

1. B. J. Simmons, N. A. Weires, J. E. Dander, N. K. Garg, "Nickel-Catalyzed Alkylation of Amide Derivatives," *ACS Catal*. **2016**, *6*, 3176-3179.
2. N. Kranidiotis-Hisatomi, M. Oestreich, "Enantio- and Regioconvergent Nickel-Catalyzed Allylic Substitution of Racemic α- or γ-Silylated Allylic Bromides with Benzylzinc Reagents," *Org. Lett.* **2022**, *24*, 4987-4991.
3. A. Krasovskiy, V. Malakhov, A. Gavryushin, P. Knochel, "Efficient synthesis of functionalized organozinc compounds by the direct insertion of zinc into organic iodides and bromides," *Angew. Chem. Int. Ed*. **2006**, *45*, 6040-6044.
4. J. Sklyaruk, J. C. Borghs, O. El-Sepelgy, M. Rueping, "Catalytic C1 Alkylation with Methanol and Isotope-Labeled Methanol," *Angew. Chem. Int. Ed.* **2019**, *58*, 775-779.
5. F. Y. Zhou, L. S. Li, K. Lin, F. Zhang, G. J. Deng, H. Gong, "Iron-Catalyzed Cleavage Reaction of Keto Acids with Aliphatic Aldehydes for the Synthesis of Ketones and Ketone Esters," *Chemistry*. **2020**, *26*, 4246-4250.
6. C. A. Wang, M. M. Rahman, E. Bisz, B. Dziuk, R. Szostak, M. Szostak, "Palladium-NHC (NHC = N-heterocyclic Carbene)-Catalyzed Suzuki–Miyaura Cross-Coupling of Alkyl Amides," *ACS Catal.* **2022**, *12*, 2426-2433.
7. J. B. Peng, B. Chen, X. X. Qi, J. Ying, X. F. Wu, "Palladium-Catalyzed Carbonylative Coupling of Aryl Iodides with Alkyl Bromides: Efficient Synthesis of Alkyl Aryl Ketones," *Adv. Synth. Catal.* **2018**, *360*, 4153-4160.
8. C. D. Aretz, H. Escobedo, B.J. Cowen, "Cyclopentane Formation from Flexible Precursors Using Samarium(II) Reagents," *Eur. J. Org. Chem.* **2018**, *2018*, 1880-1884.

9. B. Zhao, R. Shang, G. Z. Wang, S. H. Wang, H. Chen, Y. Fu, "Palladium-Catalyzed Dual Ligand-Enabled Alkylation of Silyl Enol Ether and Enamide under Irradiation: Scope, Mechanism, and Theoretical Elucidation of Hybrid Alkyl Pd(I)-Radical Species," *ACS Catal.* **2020**, *10*, 1334-1343.
10. L. J. Goossen, P. Mamone, C. Oppel, "Catalytic Decarboxylative Cross-Ketonisation of Aryl- and Alkylcarboxylic Acids using Magnetite Nanoparticles," *Adv. Synth. Catal.* **2011**, *353*, 57-63.
11. K. Huang, G. Li, W. P. Huang, D. G. Yu, Z. J. Shi, "Arylation of α-pivaloxyl ketones with arylboronic reagents via Ni-catalyzed sp3 C-O activation," *Chem. Commun.* **2011**, *47*, 7224-7226.
12. K. Polidano, B. G. Reed-Berendt, A. Basset, A. J. A. Watson, J. M. J. Williams, L. C. Morrill, "Exploring Tandem Ruthenium-Catalyzed Hydrogen Transfer and SNAr Chemistry," *Org. Lett.* **2017**, *19*, 6716-6719.
13. S. Schierle, A. Chaikuad, F. F. Lillich, X. M. Ni, S. Woltersdorf, E. Schallmayer, B. Renelt, R. Ronchetti, S. Knapp, E. Proschak, D. Merk, "Oxaprozin Analogues as Selective RXR Agonists with Superior Properties and Pharmacokinetics," *J. Med. Chem.* **2021**, *64*, 5123-5136.
14. A. J. Wommack, D. C. Moebius, A. L. Travis, J, S. Kingsbury, "Diverse Alkanones by Catalytic Carbon Insertion into the Formyl C-H Bond. Concise Access to the Natural Precursor of Achyrofuran," *Org. Lett.* **2009**, *11*, 3202-3205.
15. X. C. Sun, J. Qiu, "Preparation of dihydropyrimidin-2(1H)-one compounds as S-nitrosoglutathione reductase (GSNOR) inhibitors," CN Patent, 2011038204, Mar 31, 2011.

16. A. Kondoh, S. Inoue, R. Ojima, M. Terada, "Brønsted Base-Catalyzed Reductive Deimination of α-Iminoketones," *Bull. Chem. Soc. Jpn.* **2023**, *96*, 849-851.
17. I. Astarloa, R. SanMartin, M T. Herrero, E. Domínguez, "Aqueous α-Arylation of Mono- and Diarylethanone Enolates at Low Catalyst Loading," *Adv. Synth. Catal.* **2018**, *360*, 1711-1718.
18. A. Gomtsyan, E. K. Bayburt, R. Keddy, S. C. Turner, T. K. Jinkerson, S. Didomenico, R. J. Perner, J. R. Koenig, I. Drizin, H. A. McDonald, C. S. Surowy, P. Honore, J. Mikusa, K. C. Marsh, J. M. Wetter, C. R. Faltynek, C. H. "Lee, α-Methylation at benzylic fragment of N-aryl-N' -benzyl ureas provides TRPV1 antagonists with better pharmacokinetic properties and higher efficacy in inflammatory pain model," *Bioorg. Med. Chem. Lett*. **2007**, *17*, 3894-3899.
19. A. K. Nadipuram, M. Krishnamurthy, A. M. Ferreira, W. Li, B. M. Moore, "Synthesis and testing of novel classical cannabinoids: Exploring the side chain ligand binding pocket of the CB1 and CB2 receptors," *Bioorg. Med. Chem*. **2003**, *11*, 3121-3132.
20. S. Barman, L. You, R. Chen, V. Codrea, G. Kago, R. Edupuganti, J. Robertus, R. M. Krug, E. V. Anslyn, "Exploring naphthyl-carbohydrazides as inhibitors of influenza A viruses," *Eur. J. Med. Chem*. **2014**, *71*, 81-90.
21. A. I. Gerasyuto, M. A. Arnold, J. S. Wang, G. M. Chen, X. Y. Zhang, S. Smith, M. G. Woll, J. Baird, N. J. Zhang, N. G. Almstead, J. Narasimhan, S. Peddi, M. Dumble, J. Sheedy, M. Weetall, A. A. Branstrom, J. V. N. Prasad, G. M. Karp, "Discovery and Optimization of Indolyl-Containing 4-Hydroxy-2-Pyridone Type II DNA Topoisomerase Inhibitors Active against Multidrug Resistant Gram-negative Bacteria," *J. Med. Chem*. **2018**, *61*, 4456-4475.

^{1}H NMR (400 MHz) and ^{13}C NMR (101 MHz) in $CDCl_3$ of 1,2-Diphenylethan-1-one (**3a**)

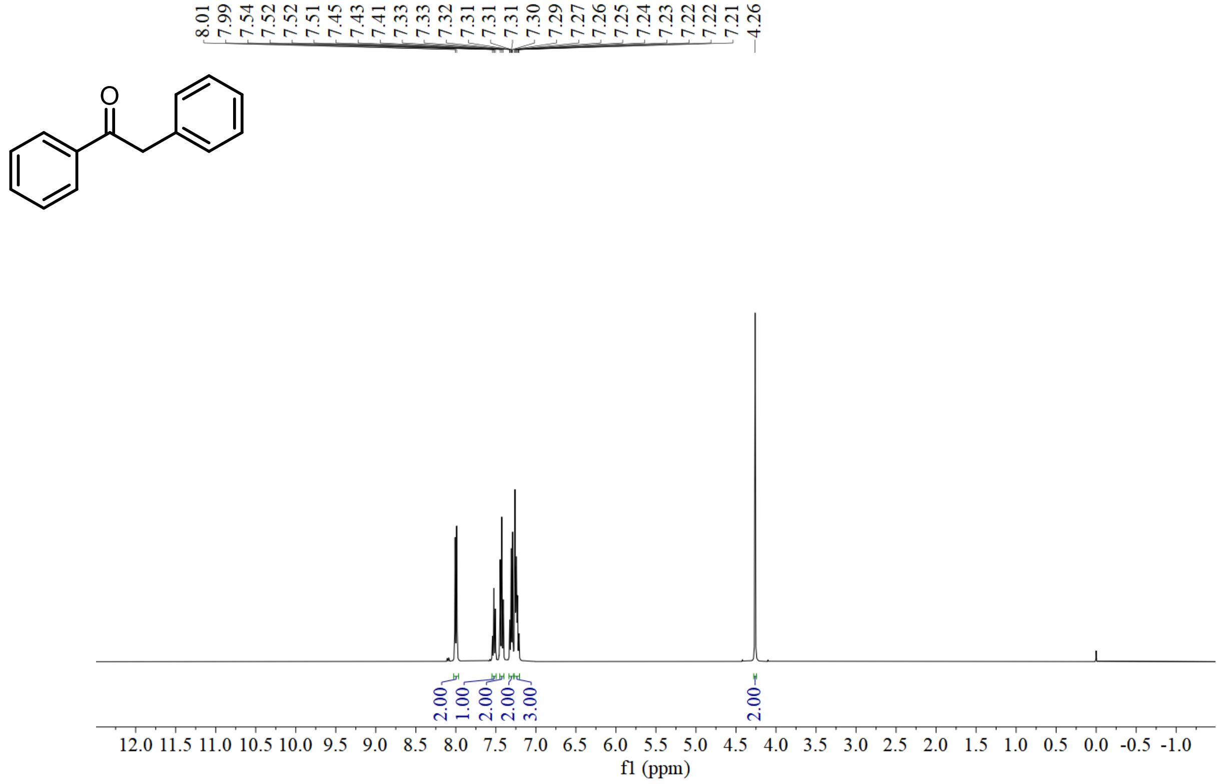

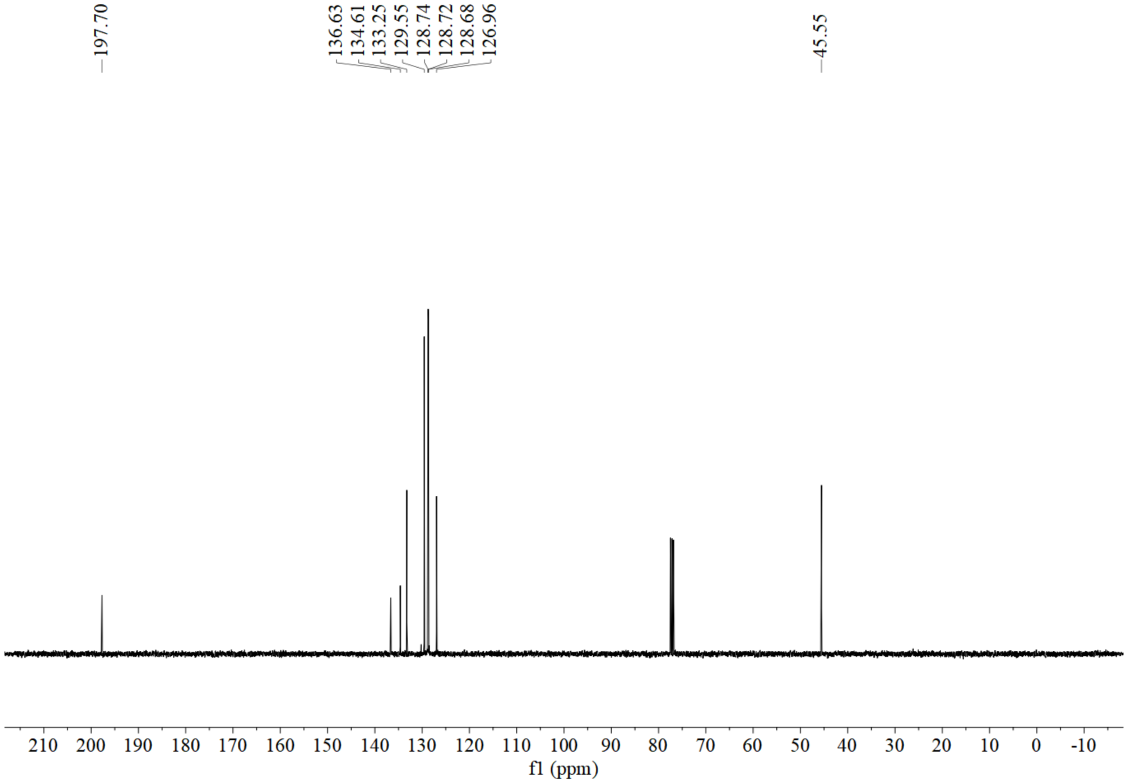

^{1}H NMR (400 MHz) and ^{13}C NMR (126 MHz) in $CDCl_3$ of 1-Phenylbutan-1-one (**3b**)

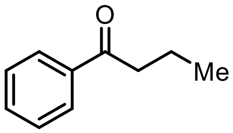

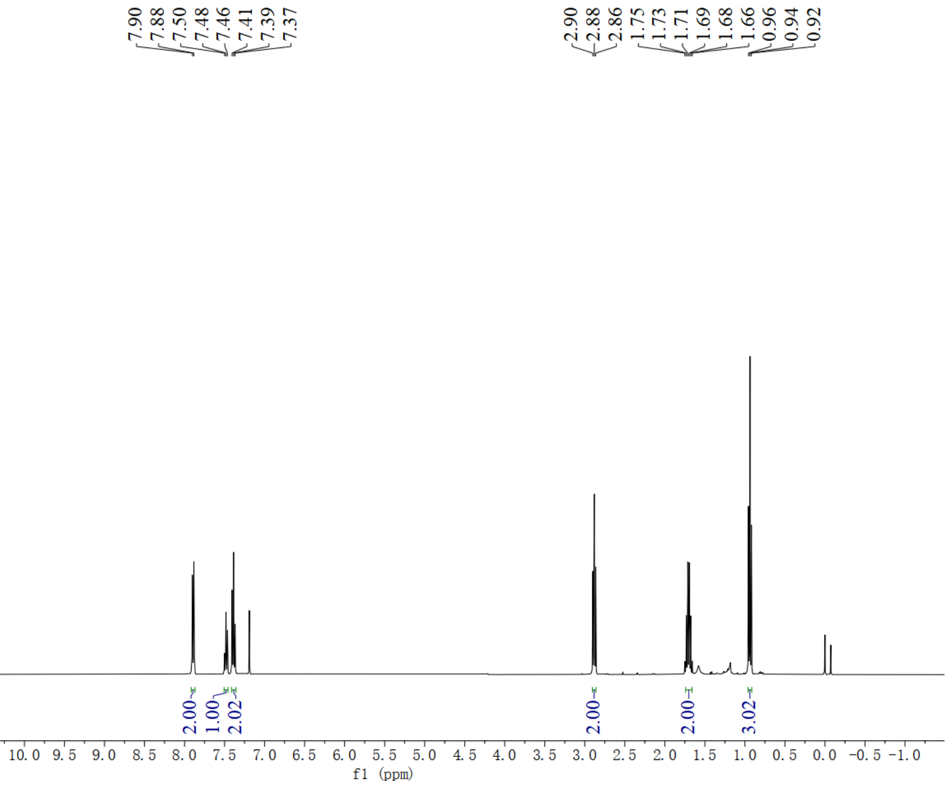

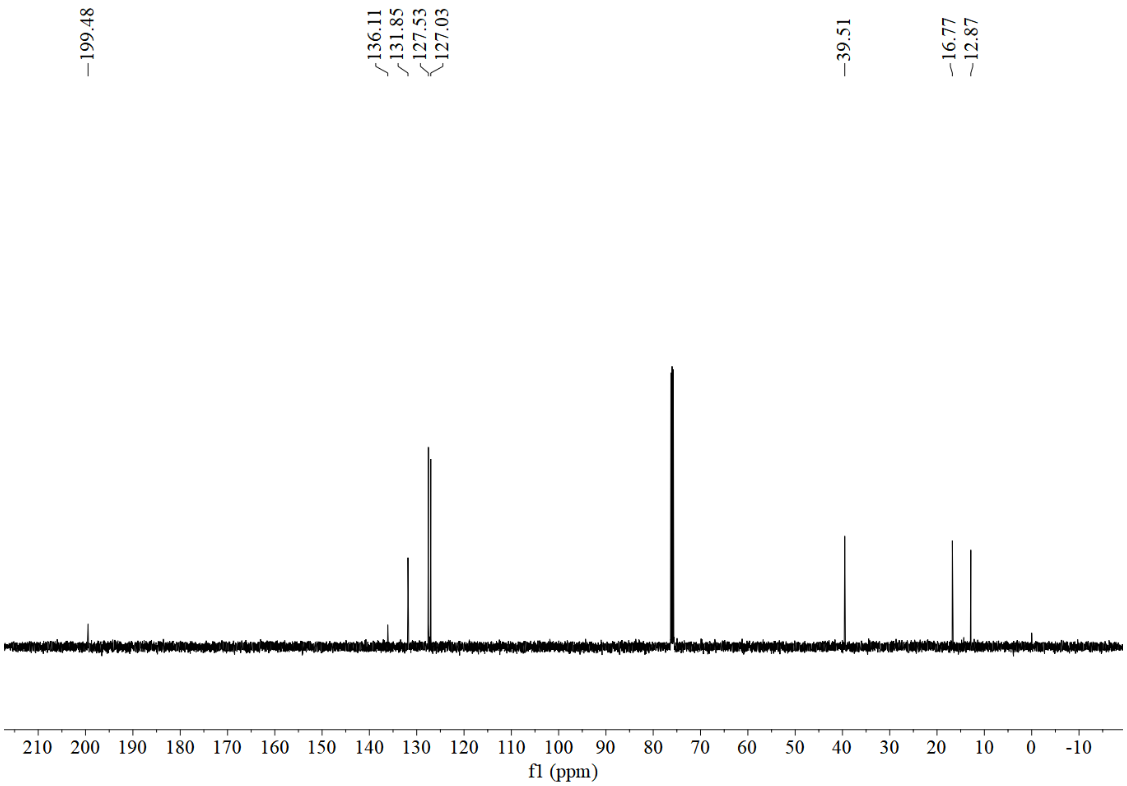

^{1}H NMR (400 MHz) and ^{13}C NMR (101 MHz) in $CDCl_3$ of 2-Methyl-1-phenylpropan-1-one (**3c**)

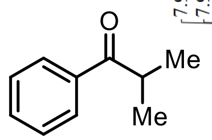

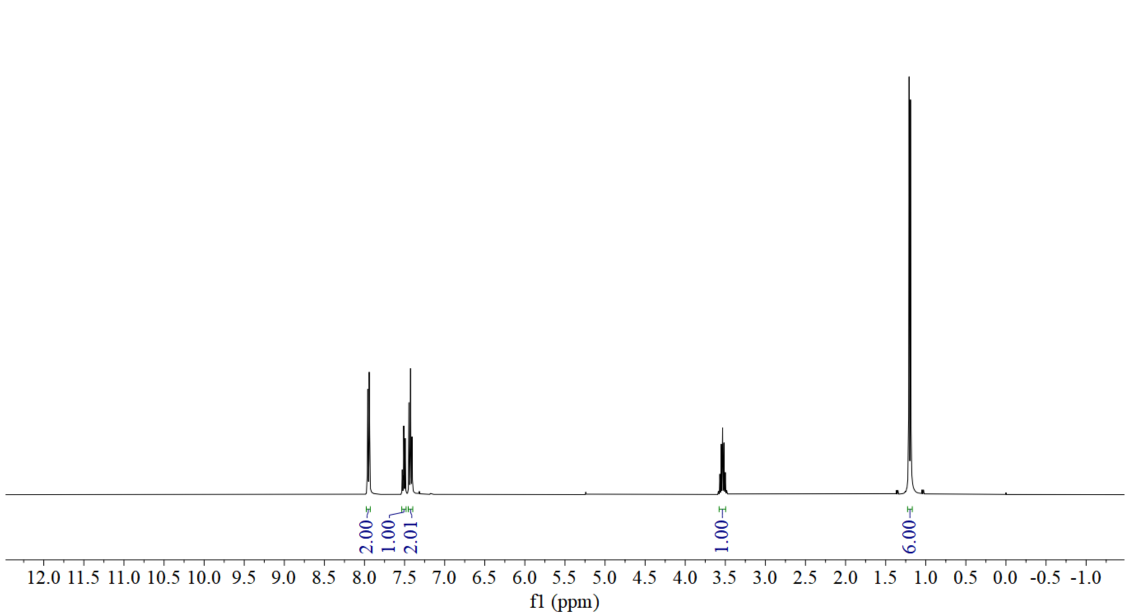

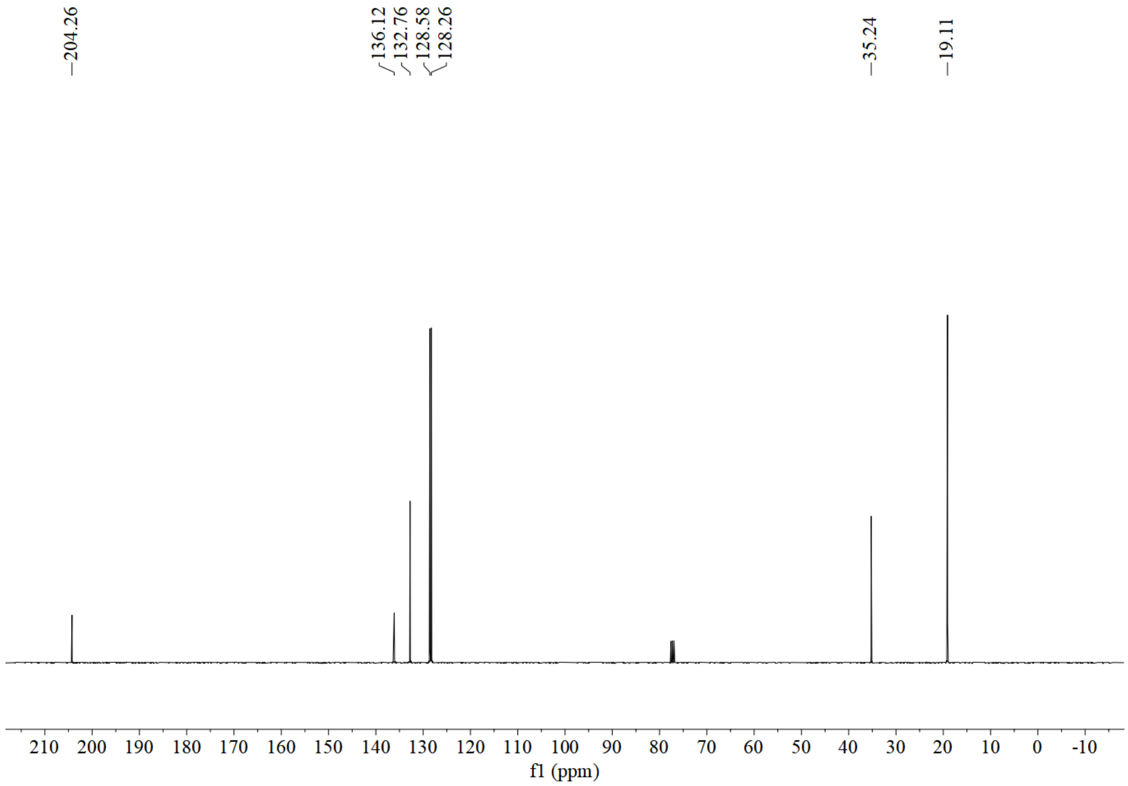

^{1}H NMR (400 MHz) and ^{13}C NMR (101 MHz) in $CDCl_3$ of 2-Methyl-1-phenylbutan-1-one (**3d**)

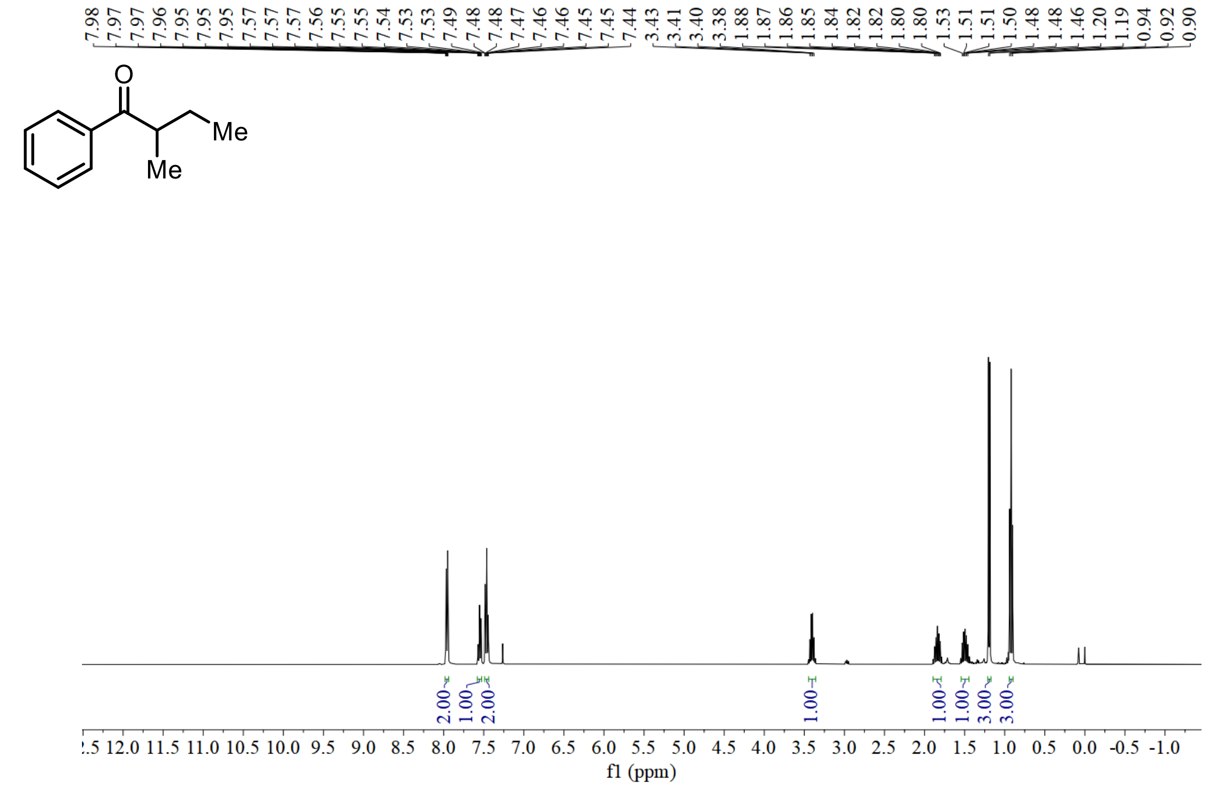

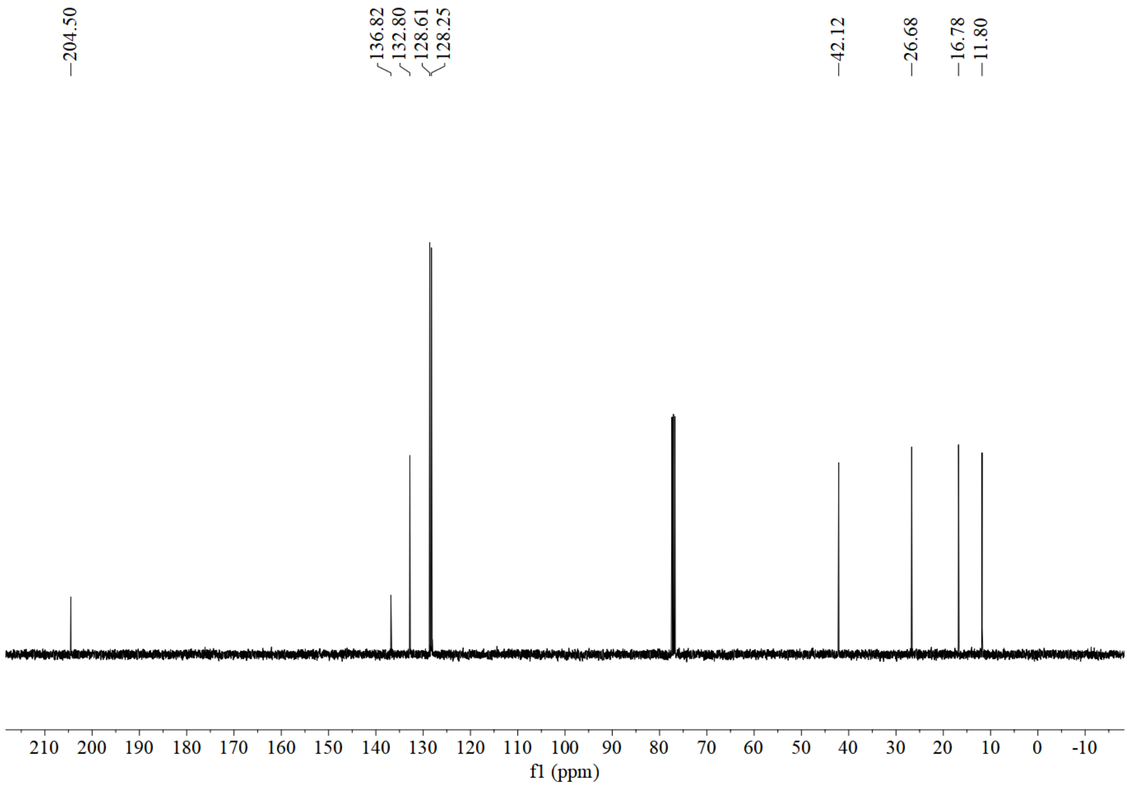

^{1}H NMR (400 MHz) and ^{13}C NMR (101 MHz) in $CDCl_3$ of 3,3-Dimethyl-1-phenylbutan-1-one (**3e**)

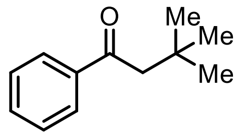

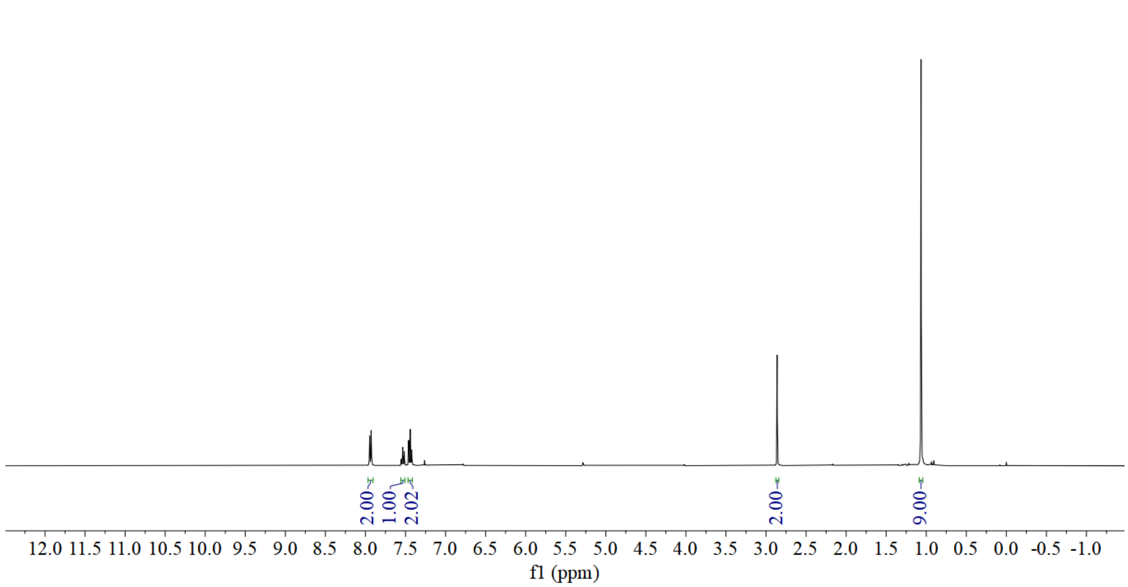

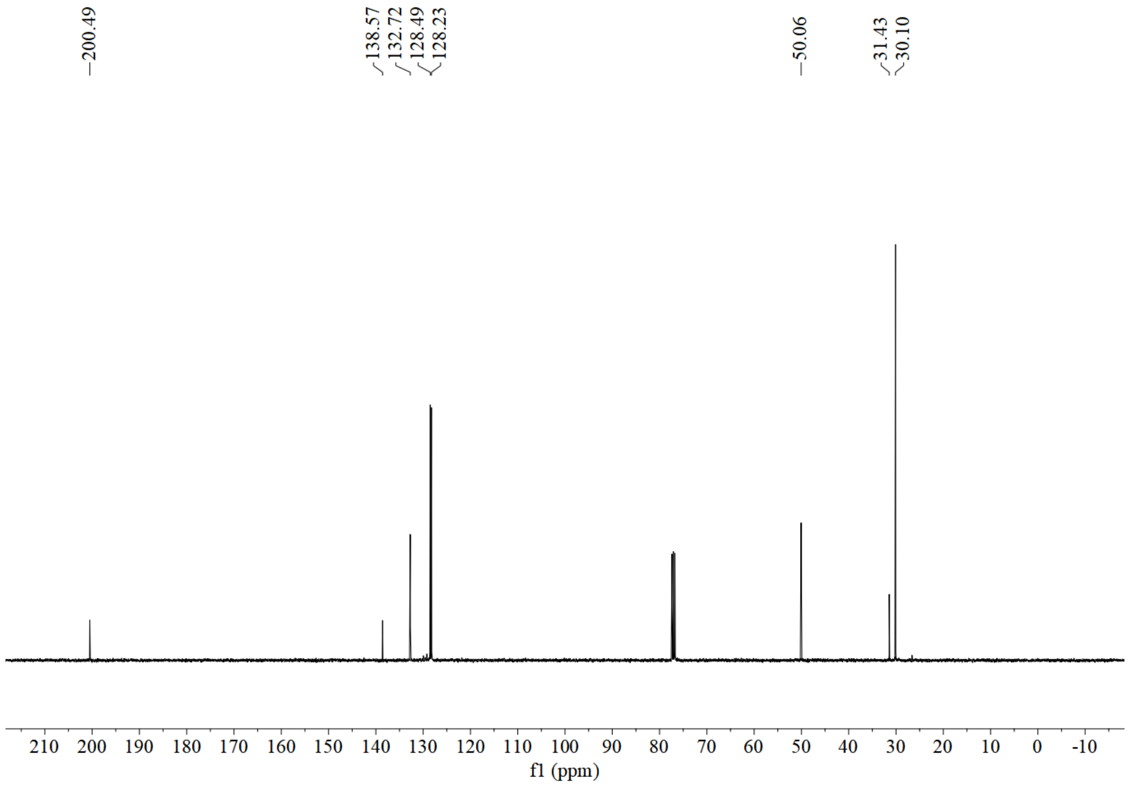

^{1}H NMR (400 MHz) and ^{13}C NMR (126 MHz) in $CDCl_3$ of Cyclopentyl(phenyl)methanone (**3f**)

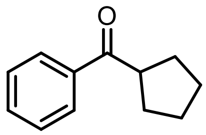

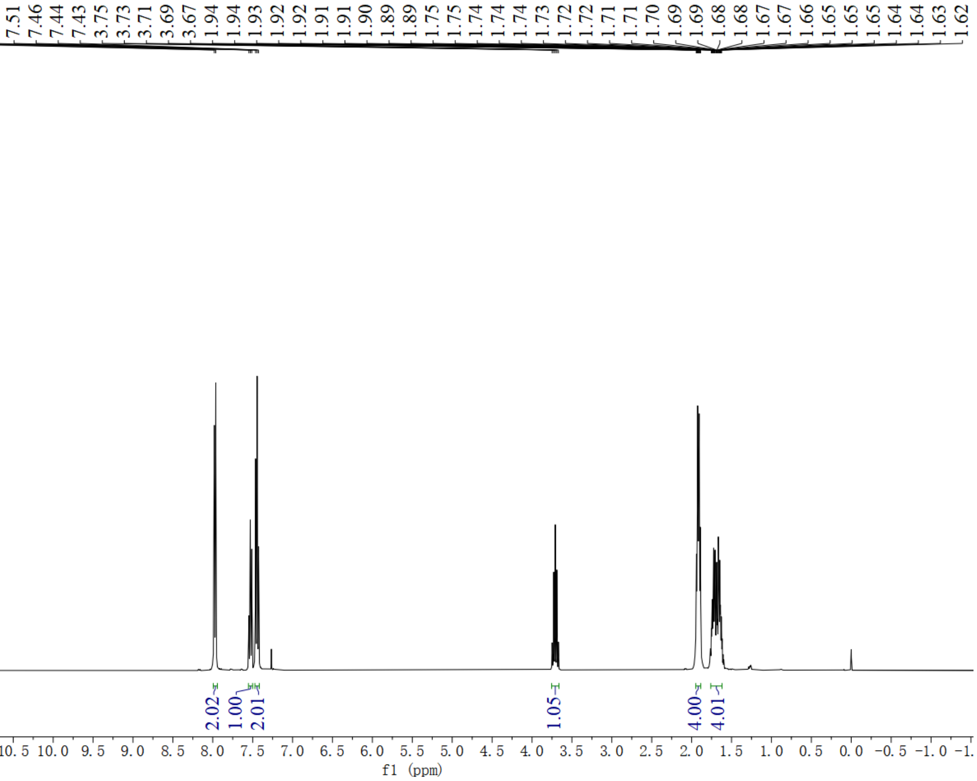

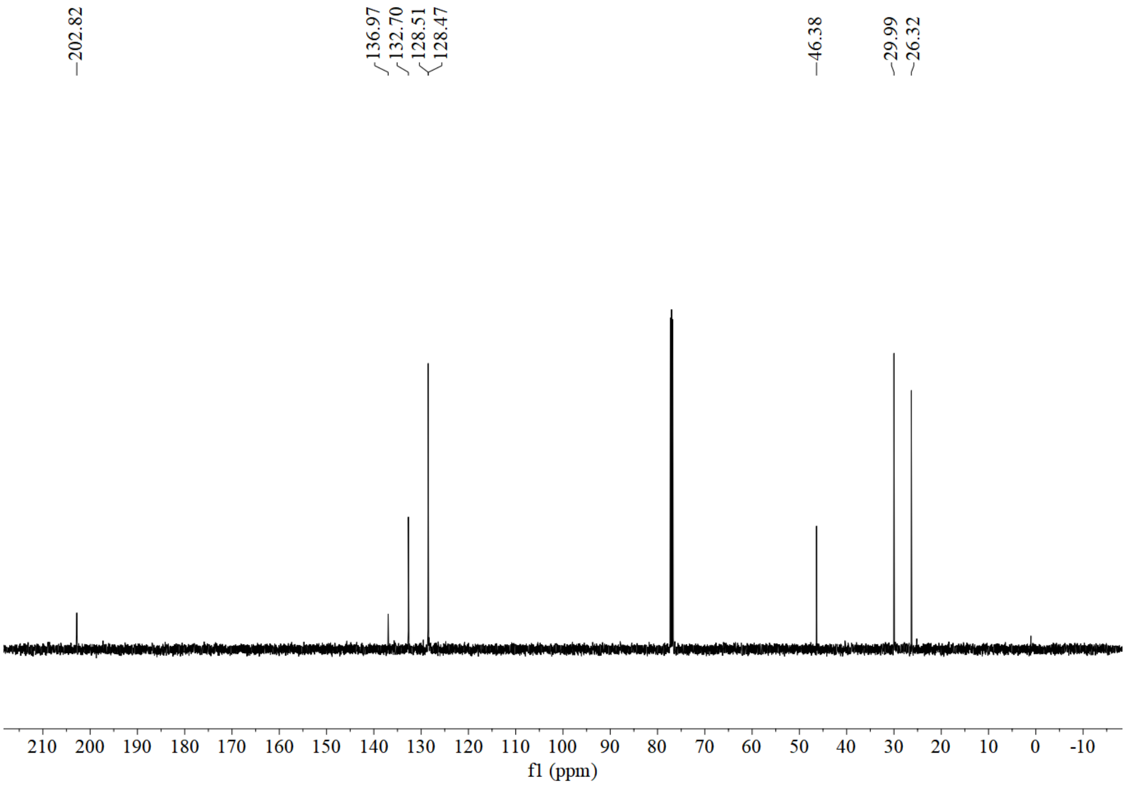

^{1}H NMR (400 MHz) and ^{13}C NMR (101 MHz) in $CDCl_3$ of Cyclohexyl(phenyl)methanone (**3g**)

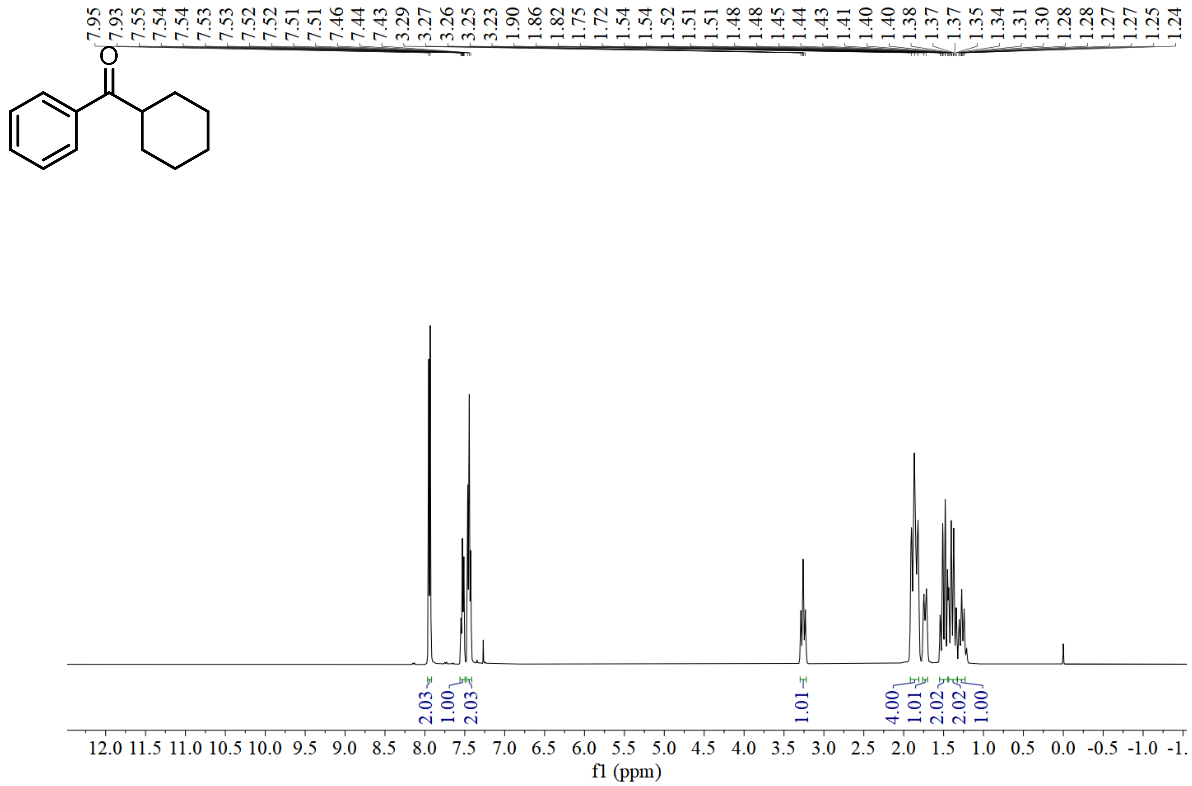

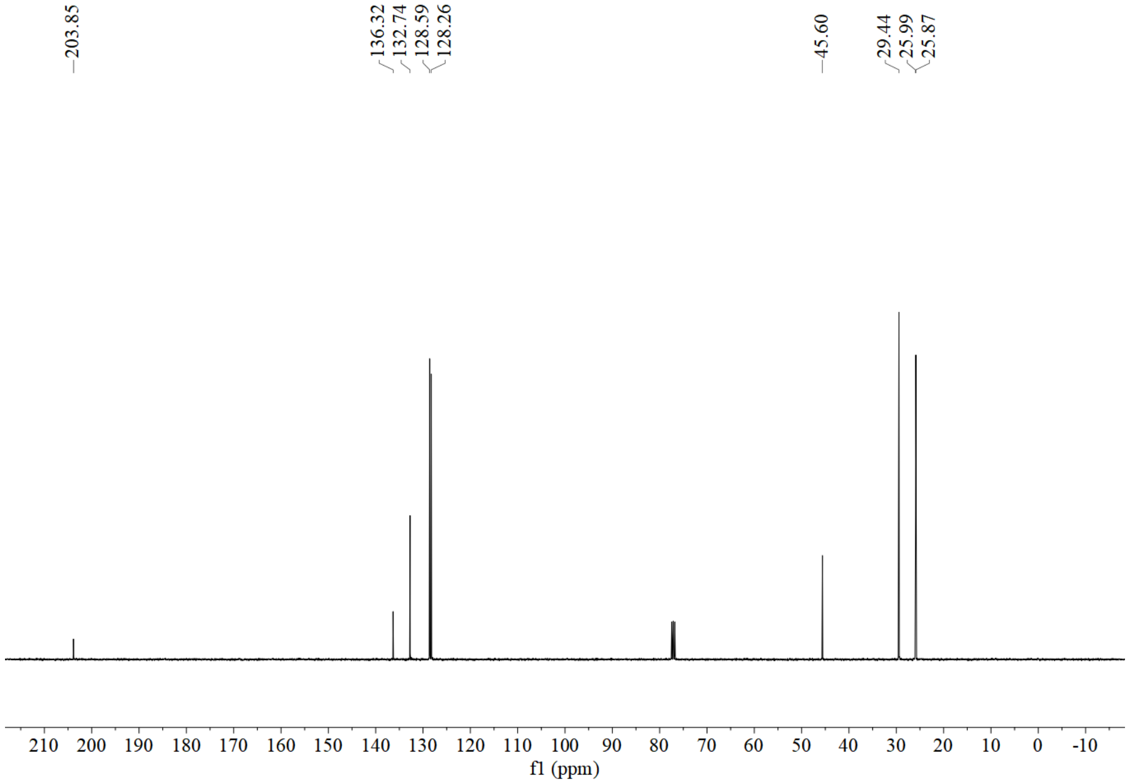

^{1}H NMR (400 MHz) and ^{13}C NMR (101 MHz) in $CDCl_3$ of 1-(Naphthalen-2-yl)butan-1-one (**3h**)

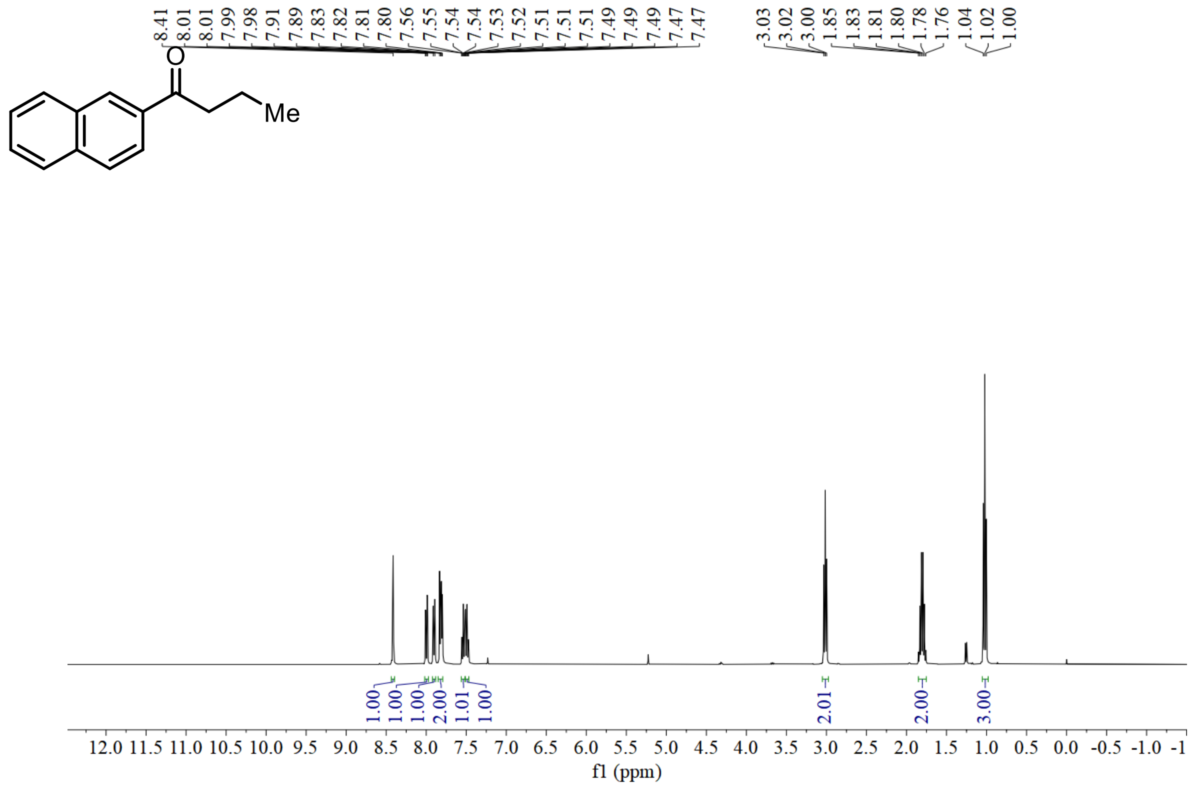

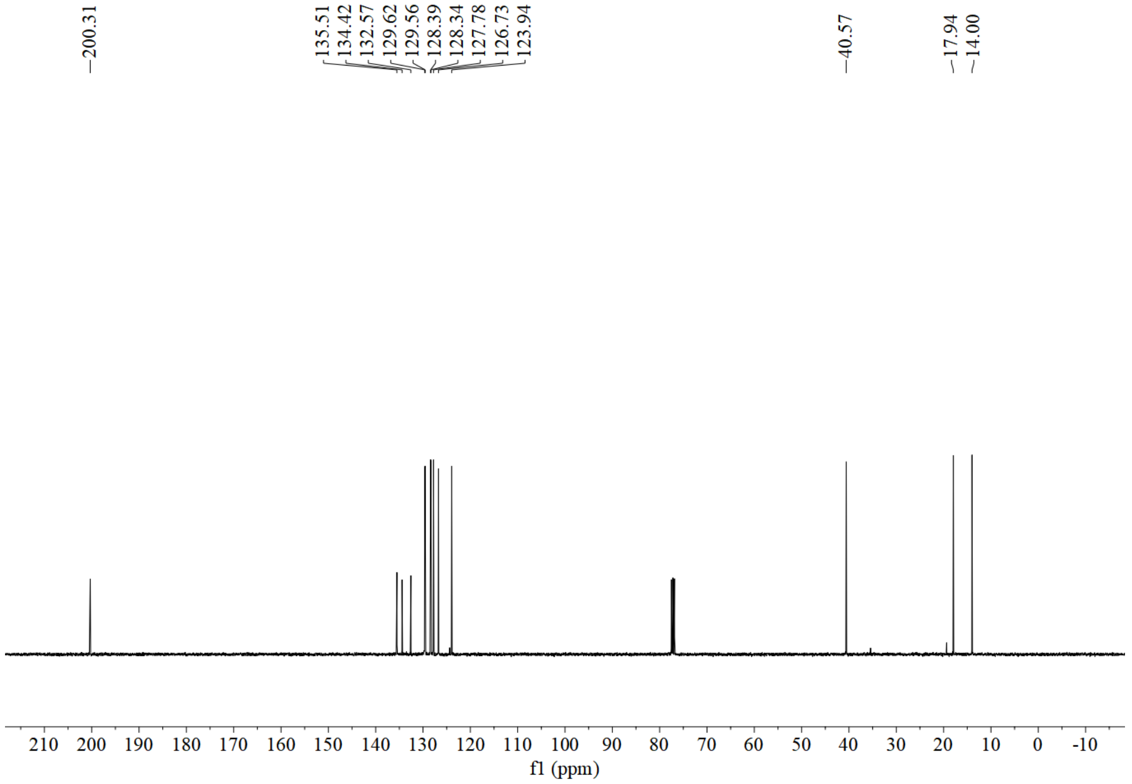

^{1}H NMR (400 MHz) and ^{13}C NMR (101 MHz) in $CDCl_3$ of 2-Methyl-1-(naphthalen-2-yl)propan-1-one (**3i**)

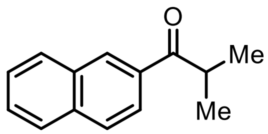

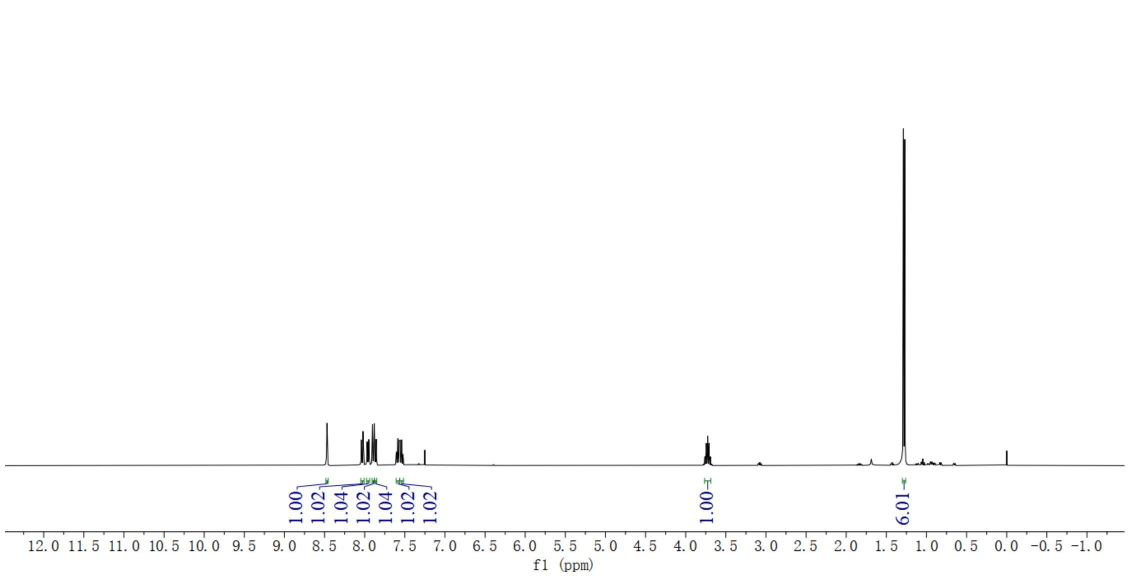

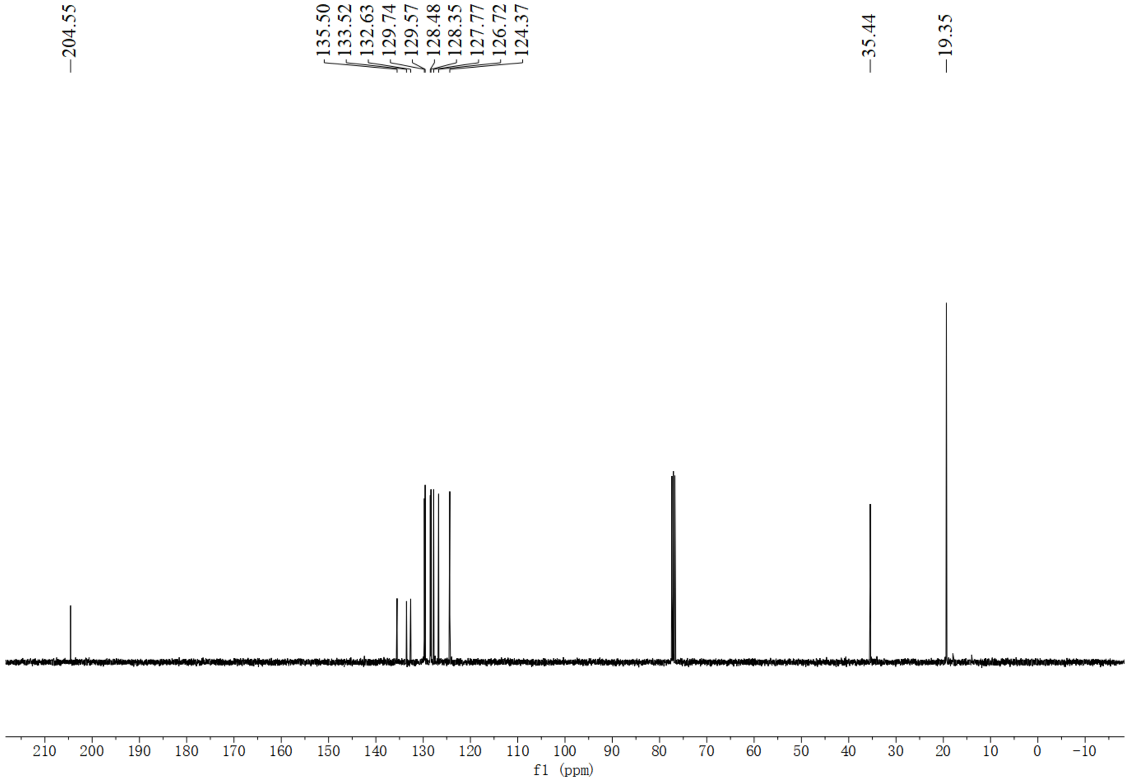

^{1}H NMR (400 MHz) and ^{13}C NMR (101 MHz) in $CDCl_3$ of 2-Methyl-1-(naphthalen-2-yl)butan-1-one (**3j**)

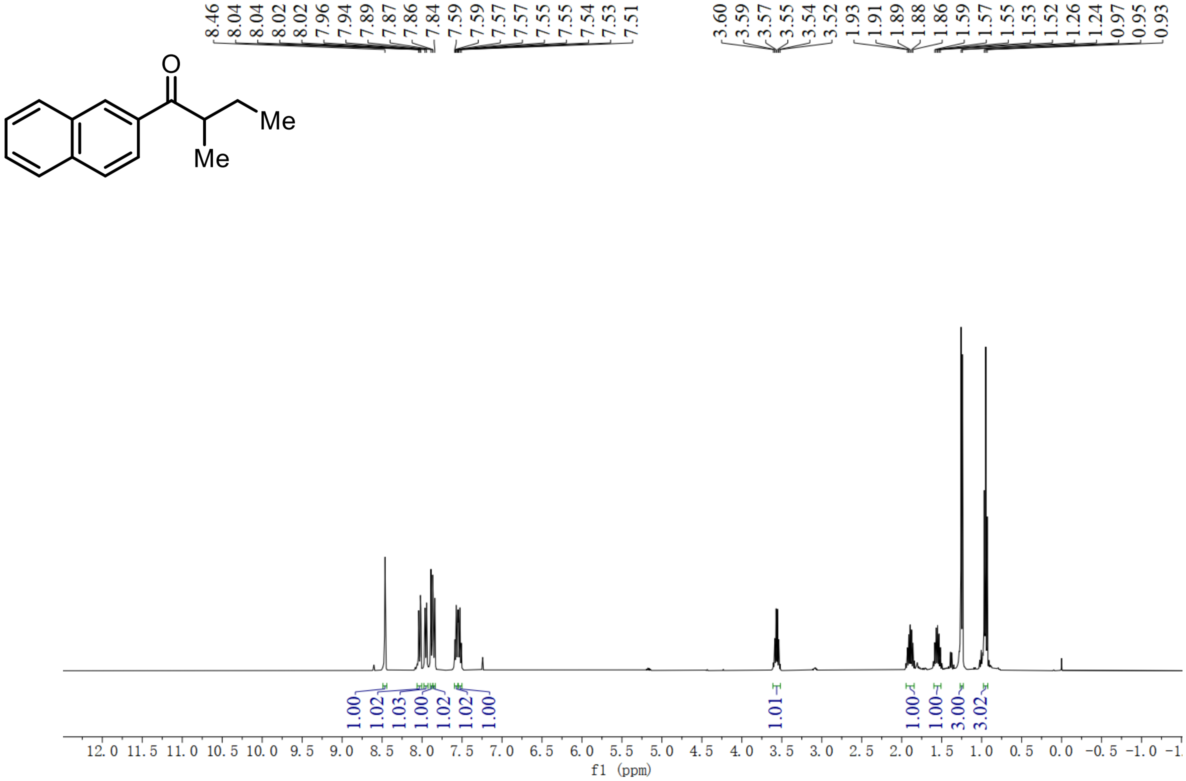

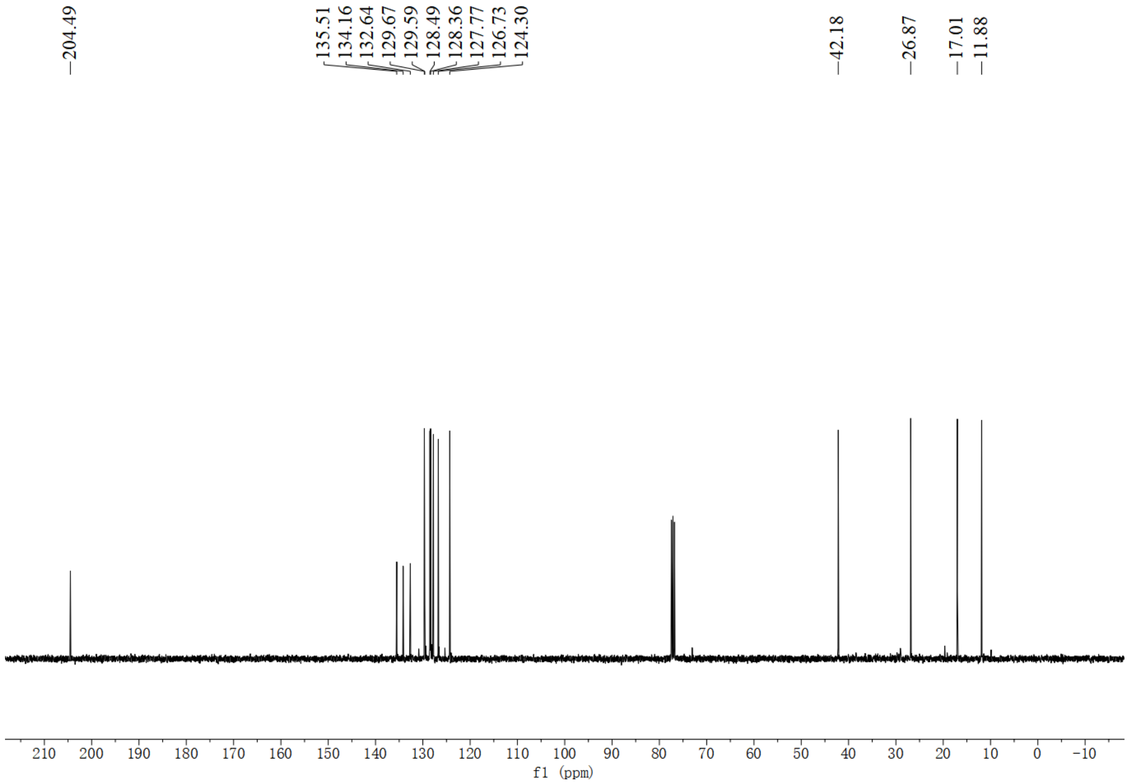

^{1}H NMR (400 MHz) and ^{13}C NMR (101 MHz) in $CDCl_3$ of 3,3-Dimethyl-1-(naphthalen-2-yl)butan-1-one (**3k**)

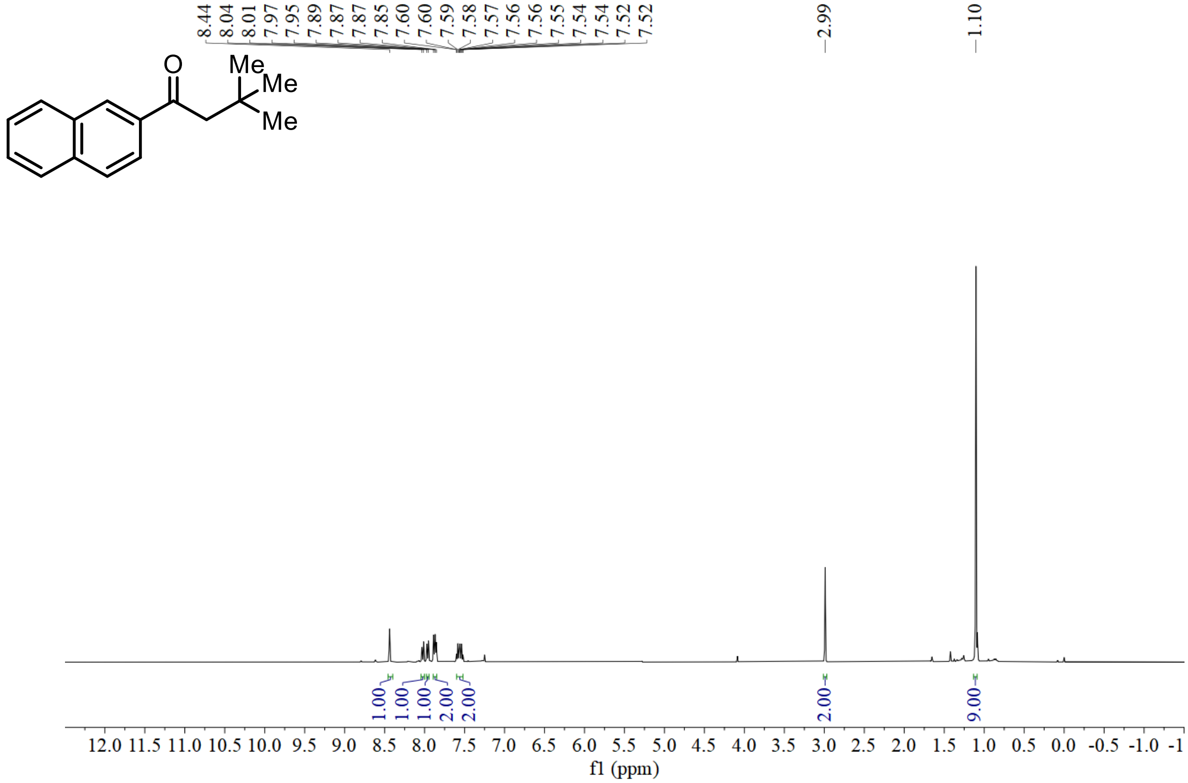

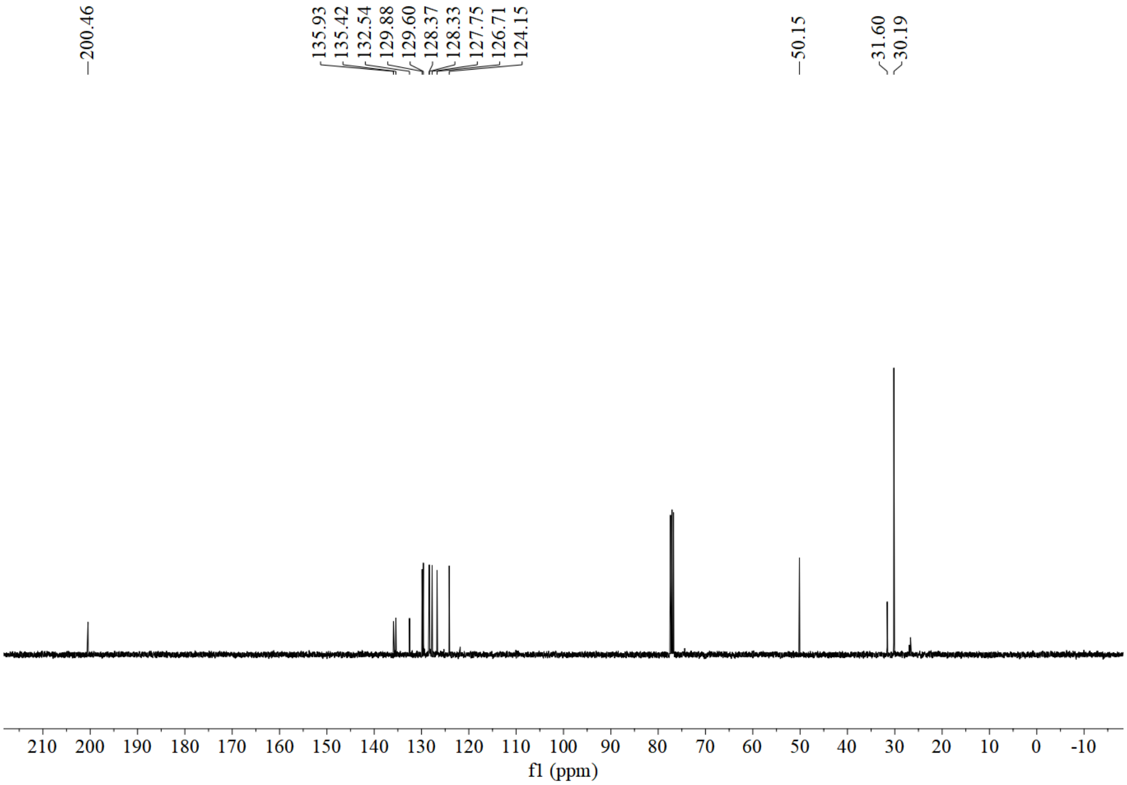

^{1}H NMR (400 MHz) and ^{13}C NMR (101 MHz) in $CDCl_3$ of Cyclopentyl(naphthalen-2-yl)methanone (**3l**)

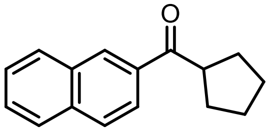

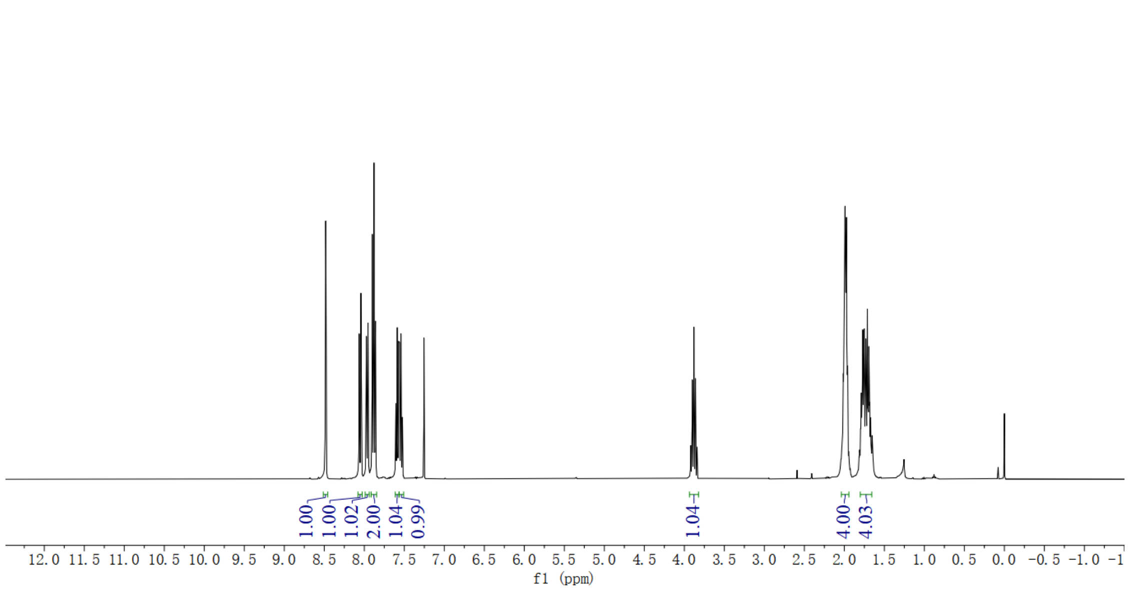

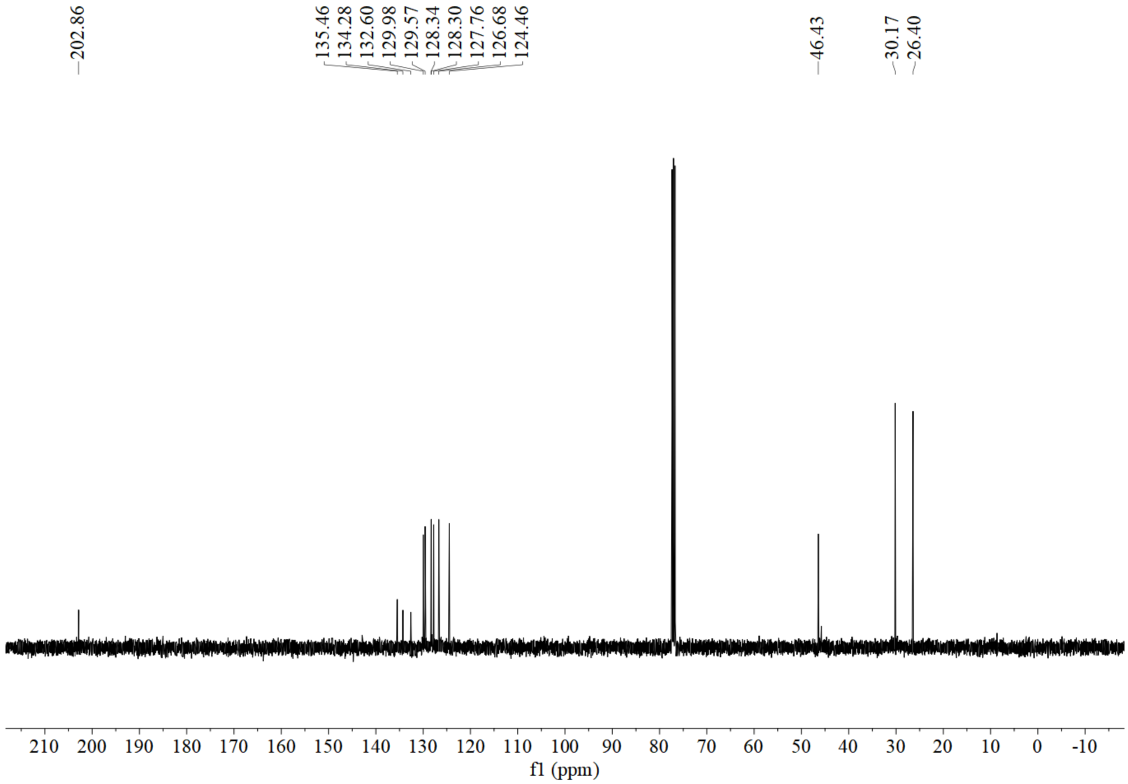

^{1}H NMR (400 MHz) and ^{13}C NMR (126 MHz) in $CDCl_3$ of Cyclohexyl(naphthalen-2-yl)methanone (**3m**)

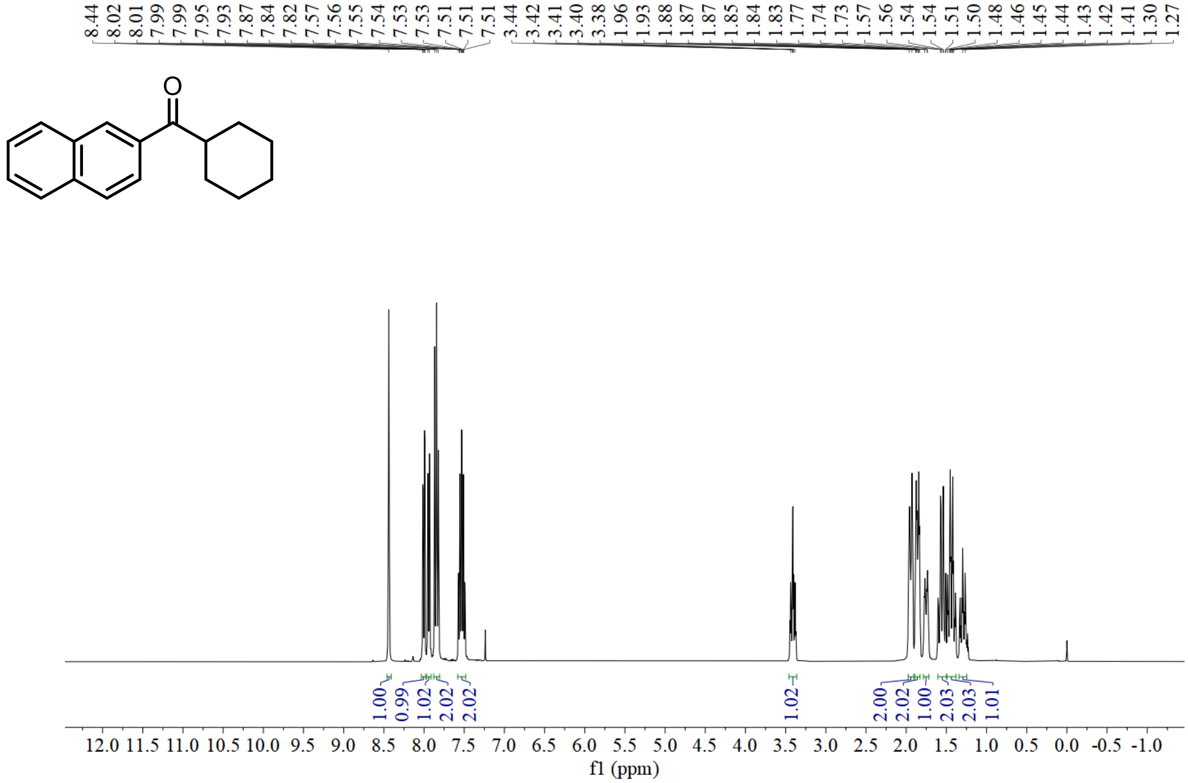

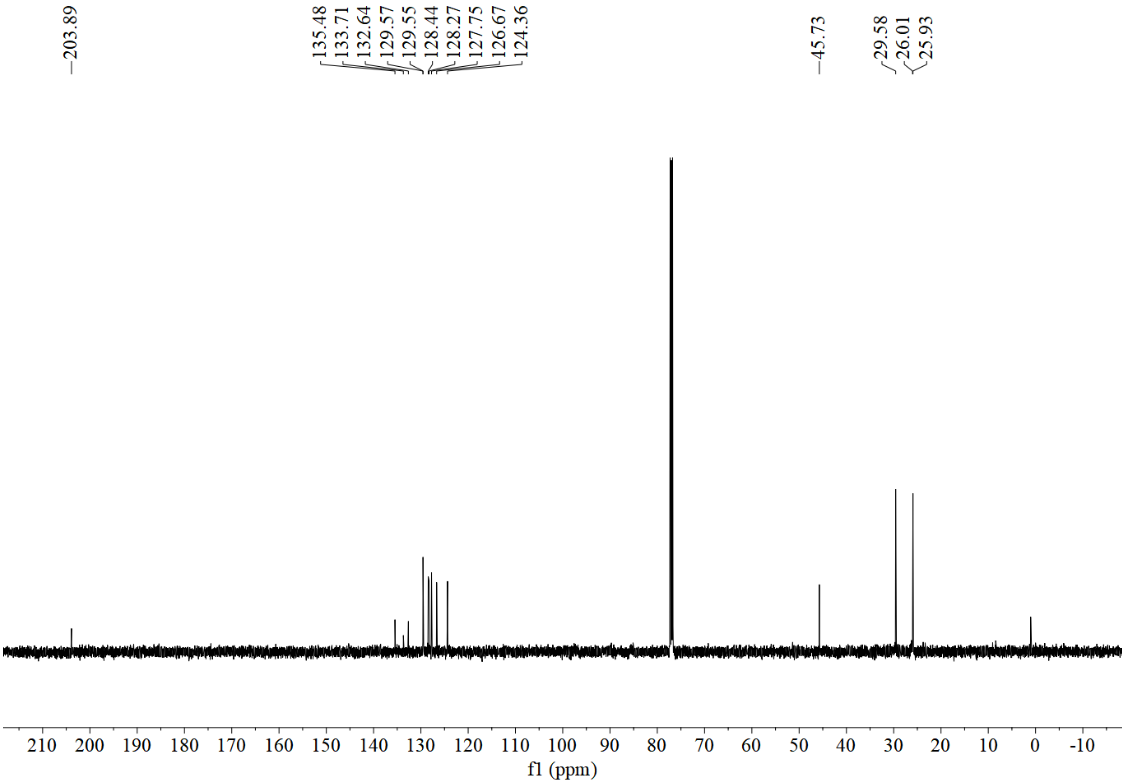

^{1}H NMR (400 MHz) and ^{13}C NMR (101 MHz) in $CDCl_3$ of 2-Phenyl-1-(*p*-tolyl)ethan-1-one (**3n**)

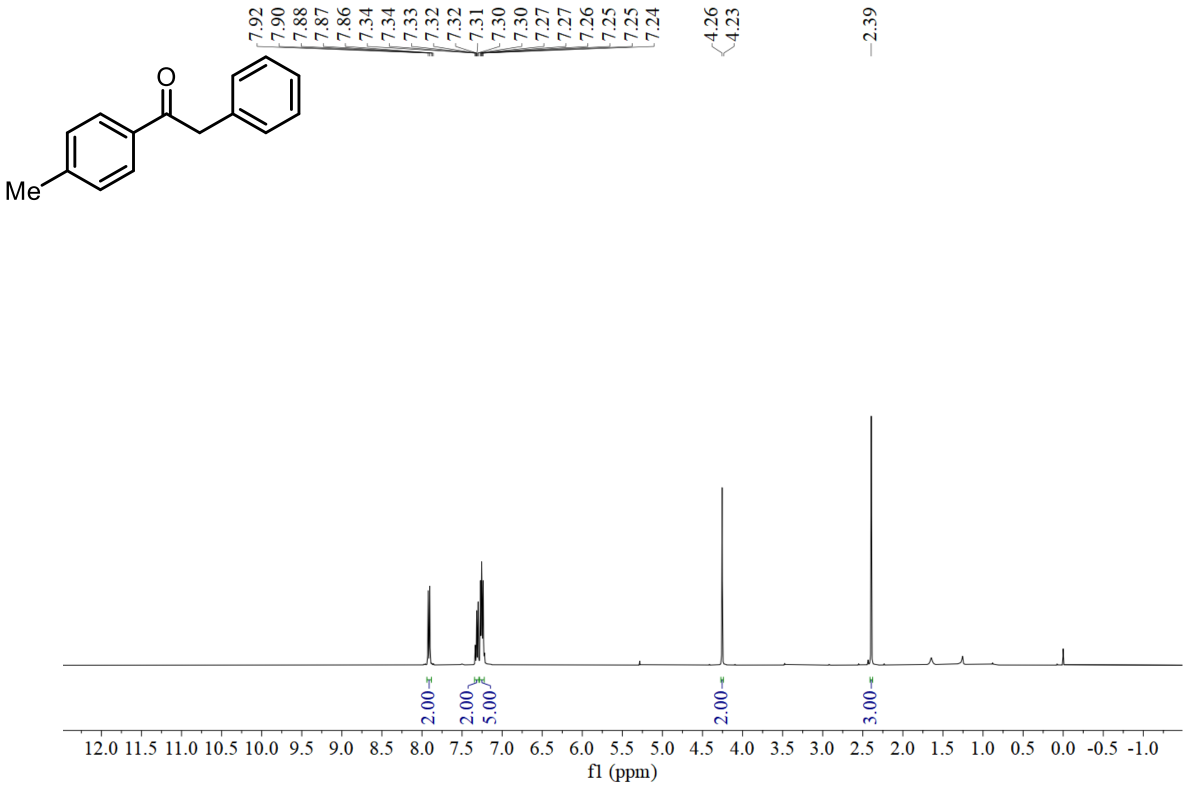

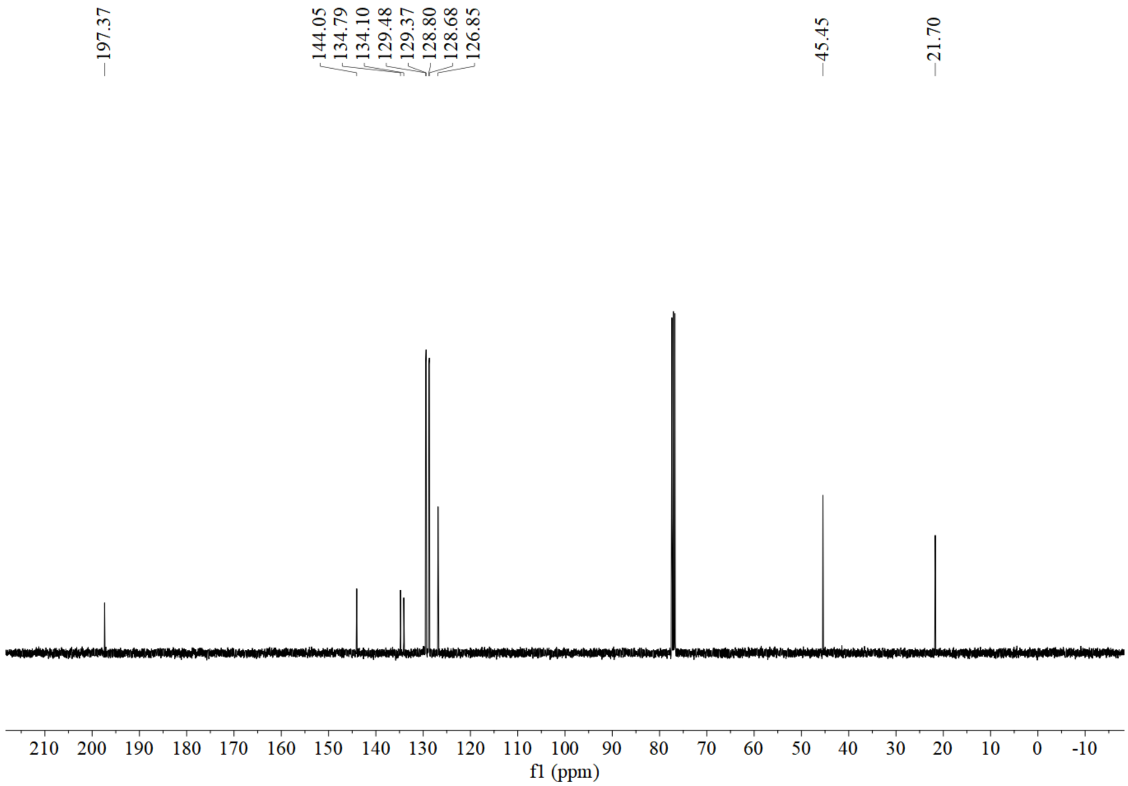

^{1}H NMR (500 MHz) and ^{13}C NMR (126 MHz) in $CDCl_3$ of 1-(4-Methoxyphenyl)-2-phenylethan-1-one (**3o**)

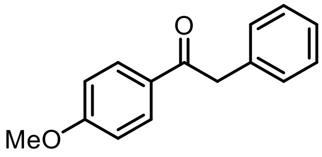

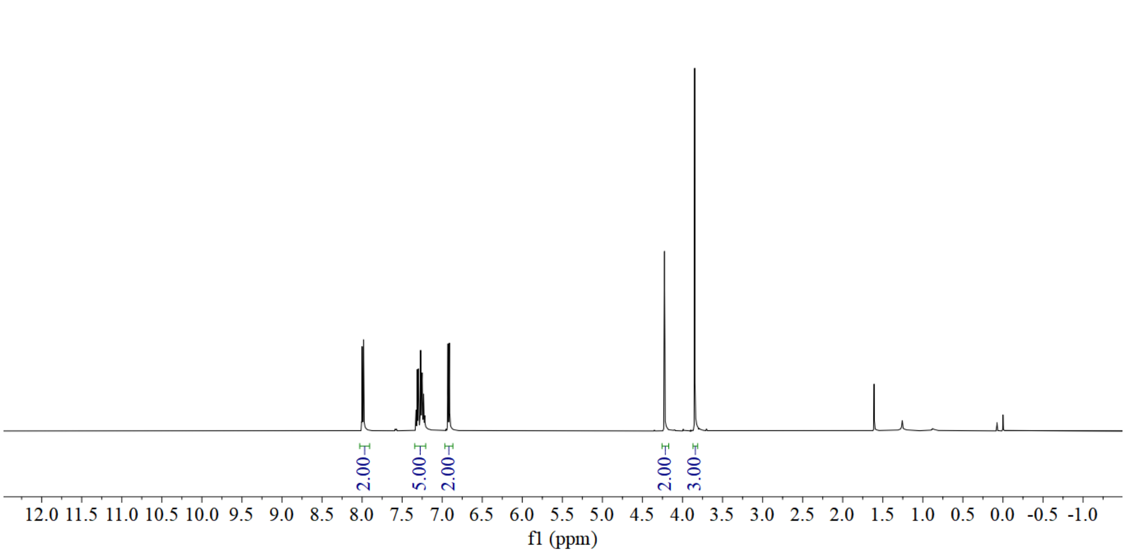

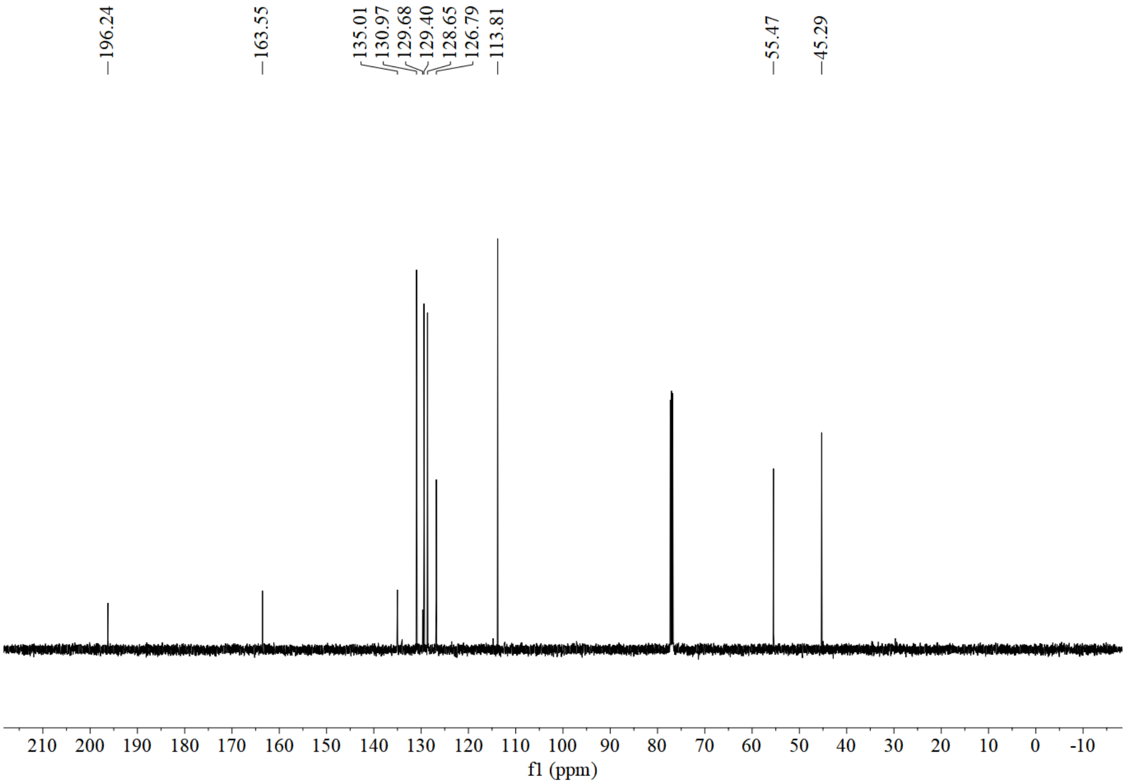

^{1}H NMR (400 MHz), ^{13}C NMR (101 MHz), and ^{19}F NMR (376 MHz) in $CDCl_3$ of 1-(4-Fluorophenyl)-2-phenylethan-1-one (**3p**)

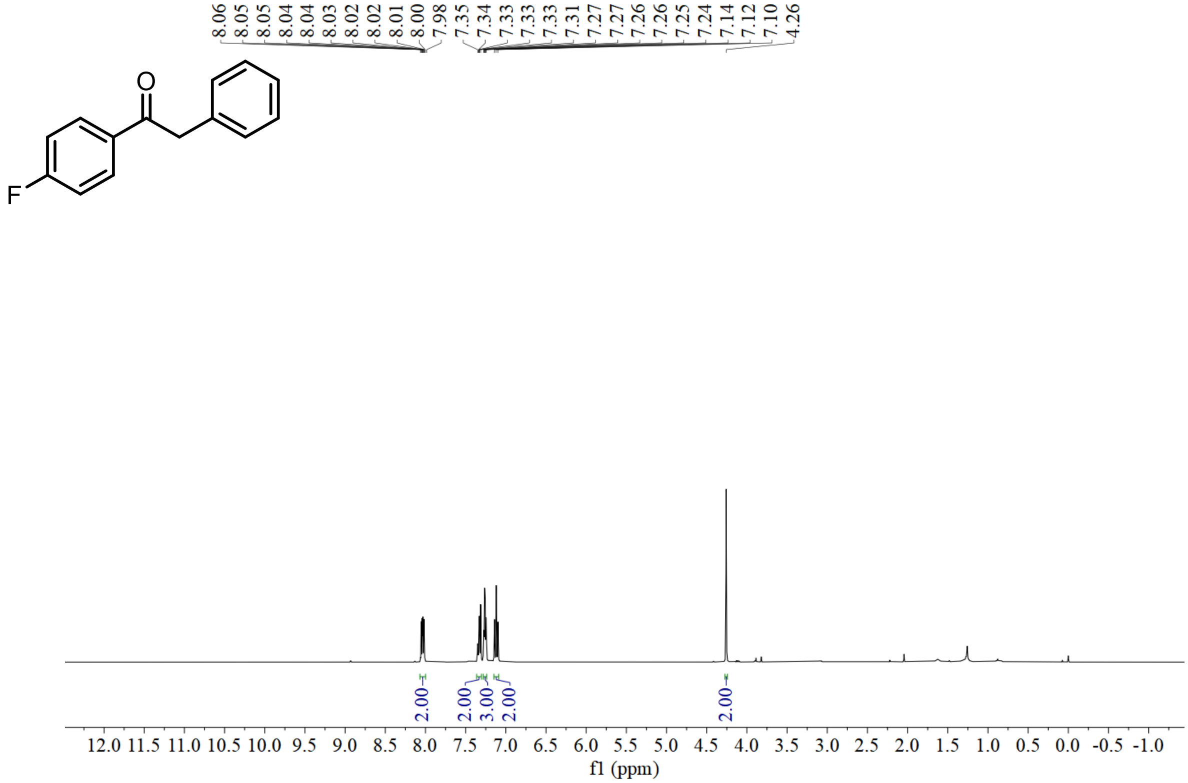

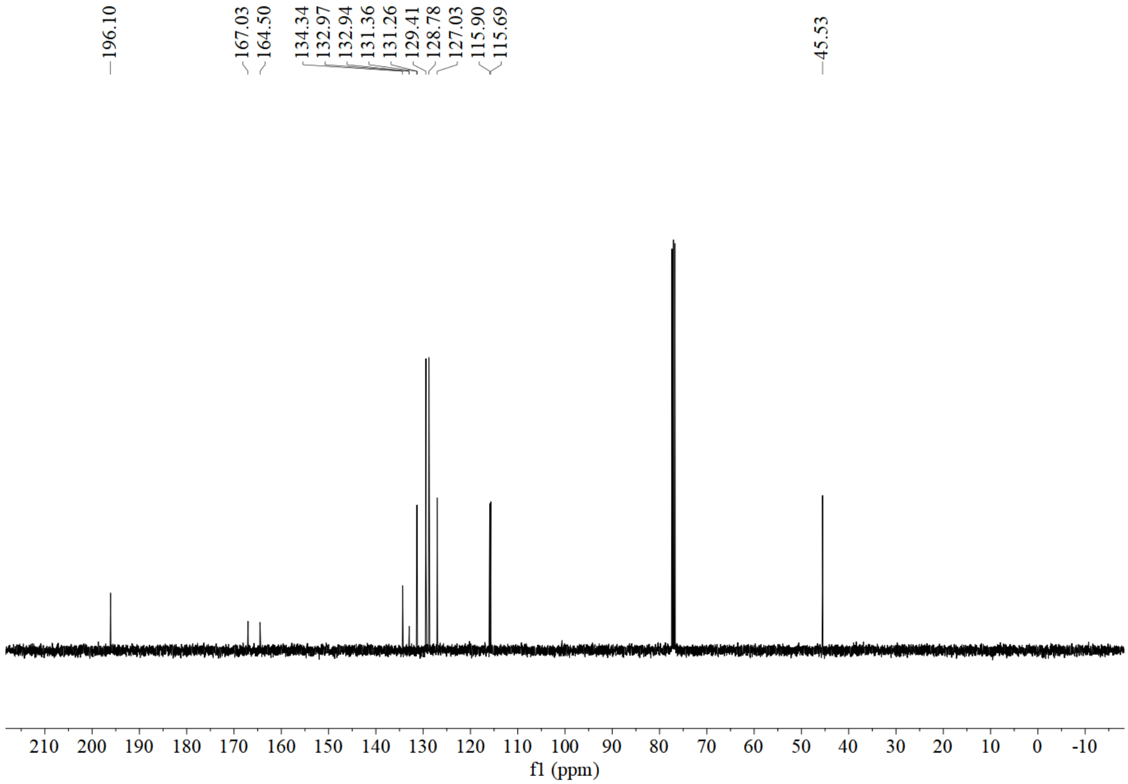

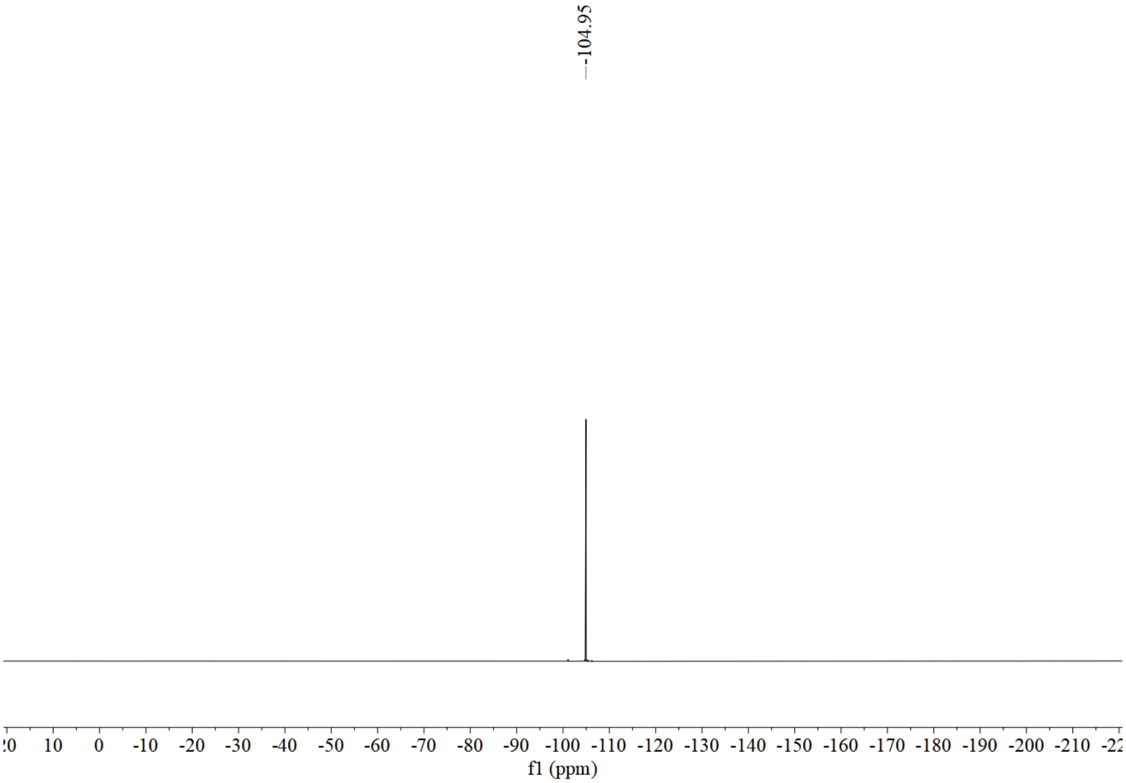
-104.95
20
10
0
-10
-20
-30
-40
-50
-60
-70
-80
-90
-100
-110
-120
-130
-140
-150
-160
-170
-180
-190
-200
-210
-22
f1 (ppm)

^{1}H NMR (400 MHz), ^{13}C NMR (101 MHz), and ^{19}F NMR (376 MHz) in $CDCl_3$ of 2-Phenyl-1-(4-(trifluoromethyl)phenyl)ethan-1-one (**3q**)

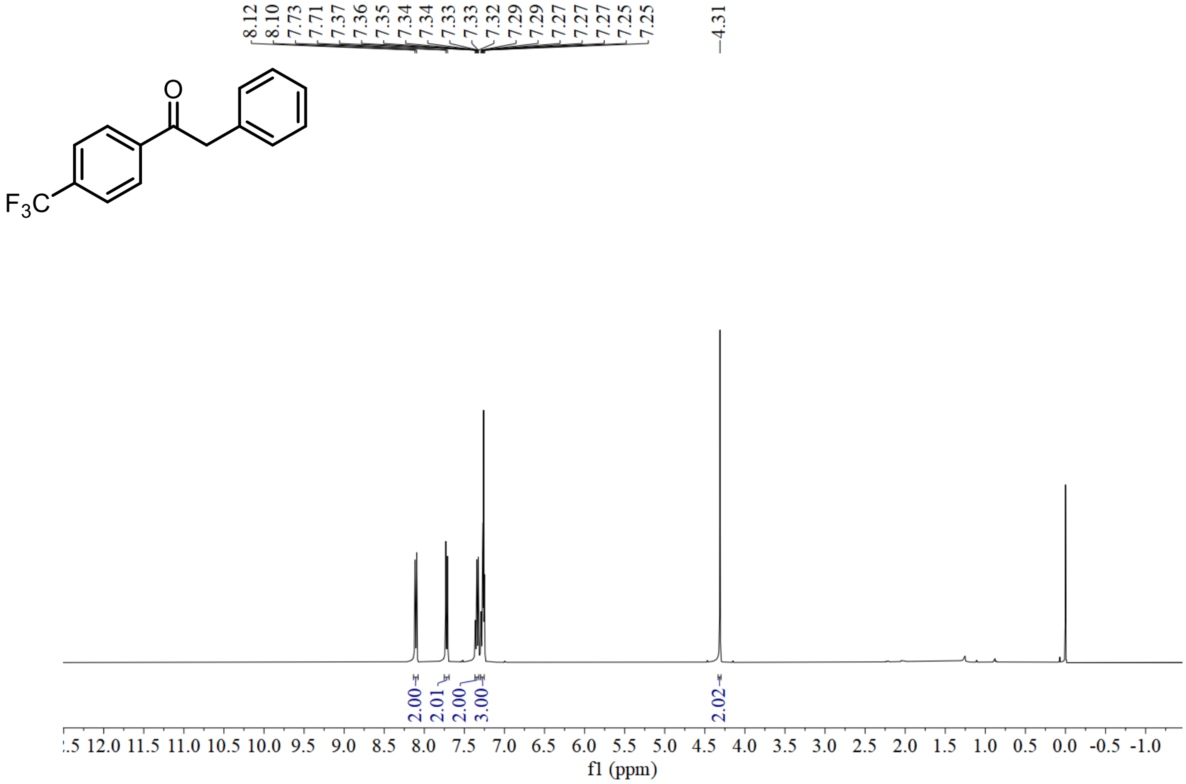

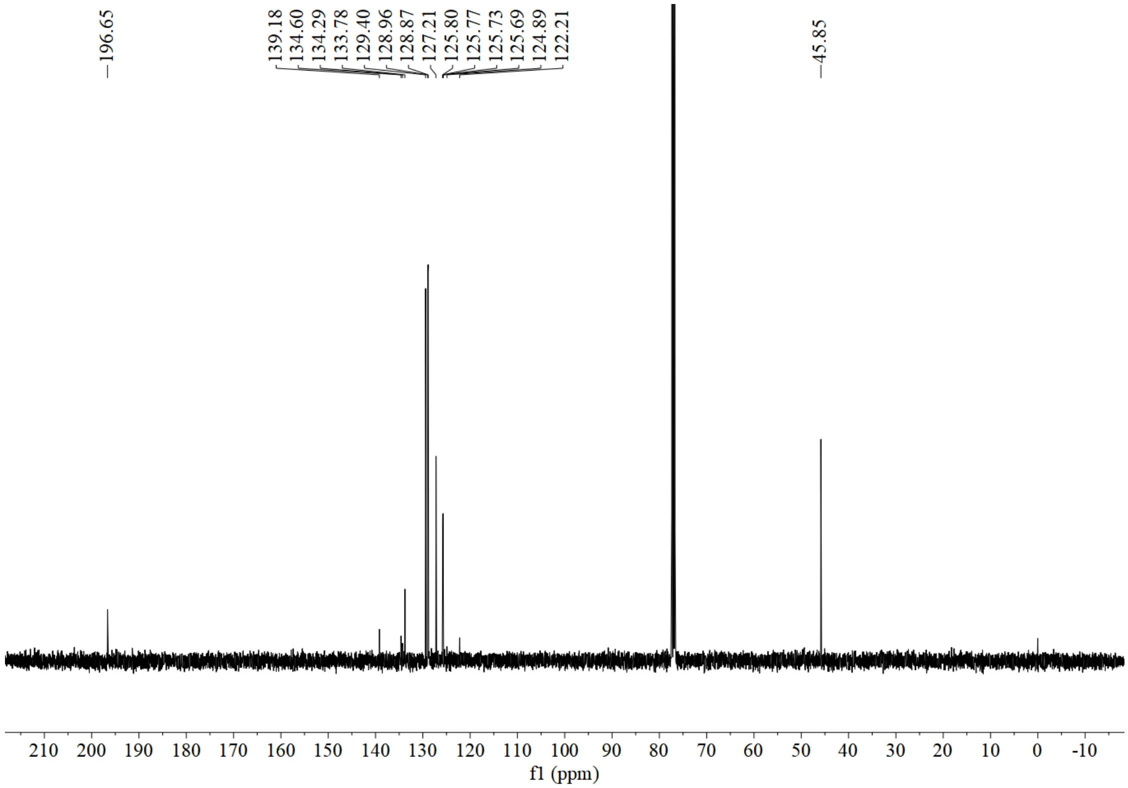

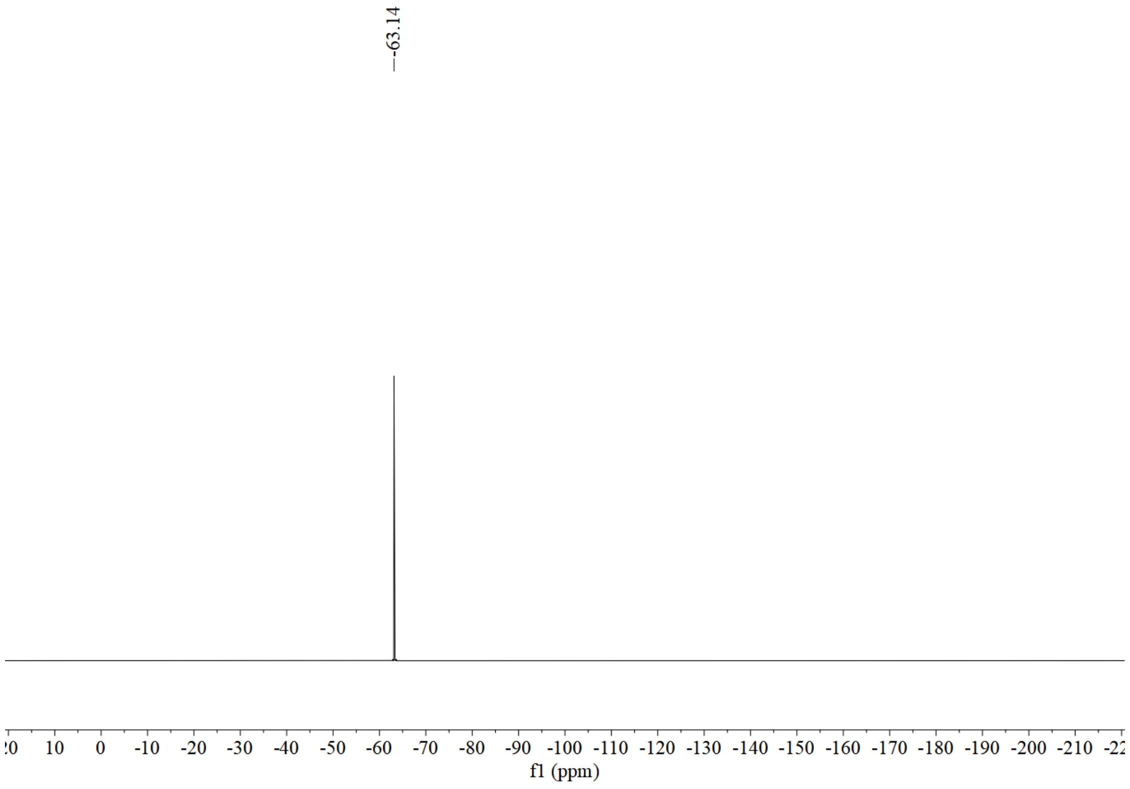
-63.14
20 10 0 -10 -20 -30 -40 -50 -60 -70 -80 -90 -100 -110 -120 -130 -140 -150 -160 -170 -180 -190 -200 -210 -22
f1 (ppm)

^{1}H NMR (400 MHz) and ^{13}C NMR (101 MHz) in $CDCl_3$ of 1-([1,1'-Biphenyl]-4-yl)-2-phenylethan-1-one (**3r**)

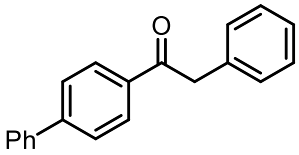

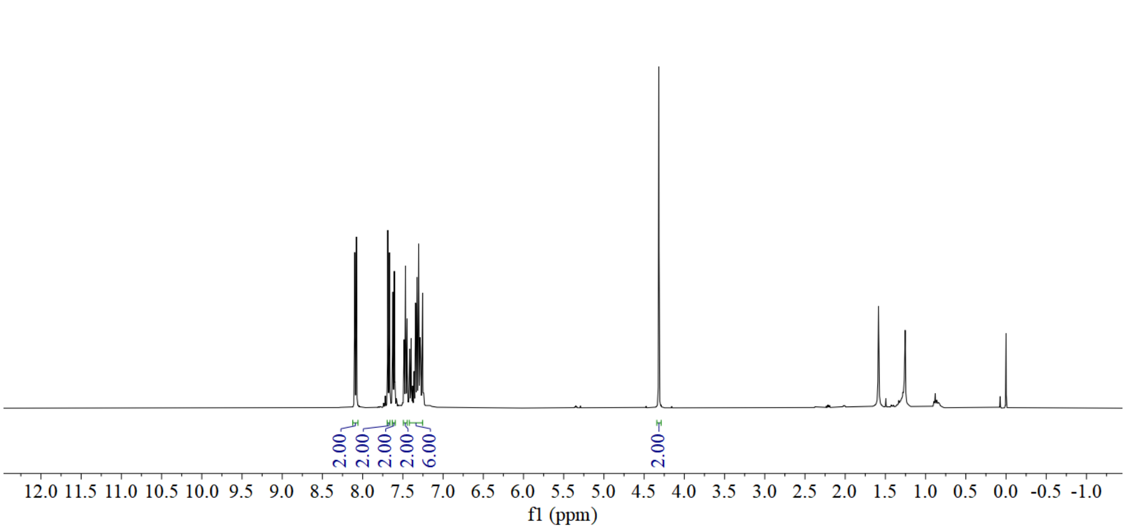

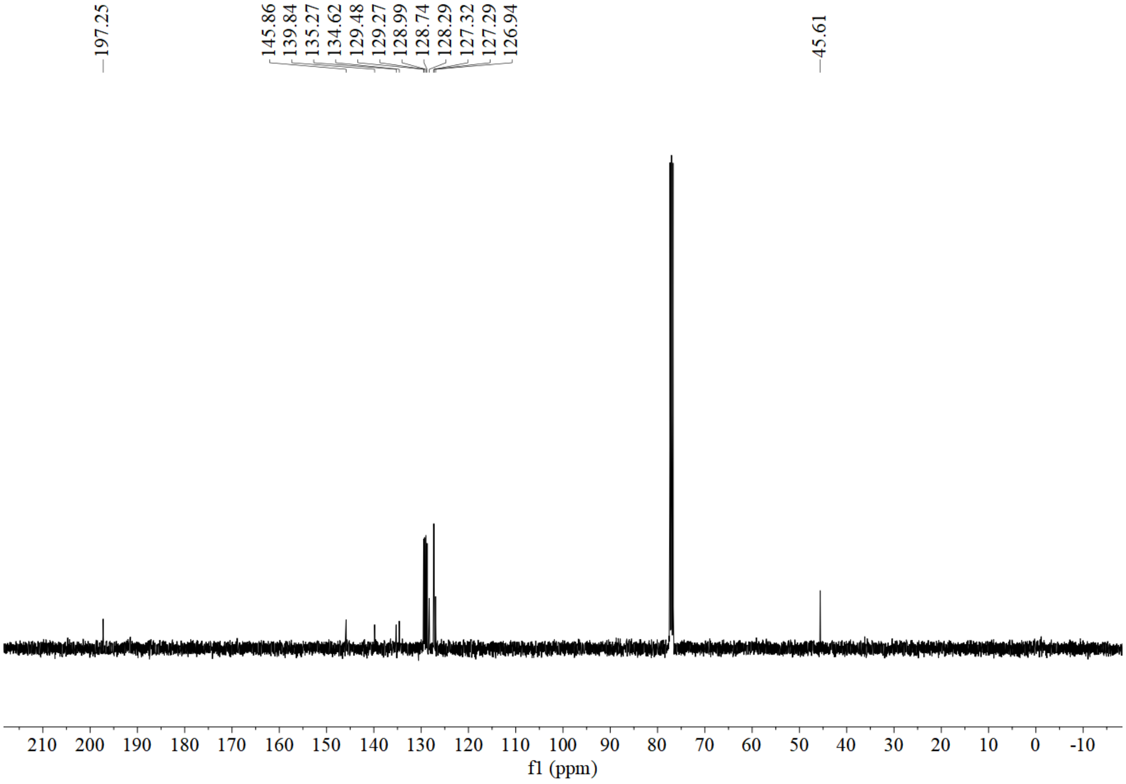

^{1}H NMR (400 MHz) and ^{13}C NMR (101 MHz) in $CDCl_3$ of 1-(4-Phenoxyphenyl)-2-phenylethan-1-one (**3s**)

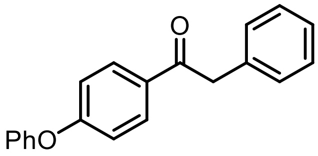

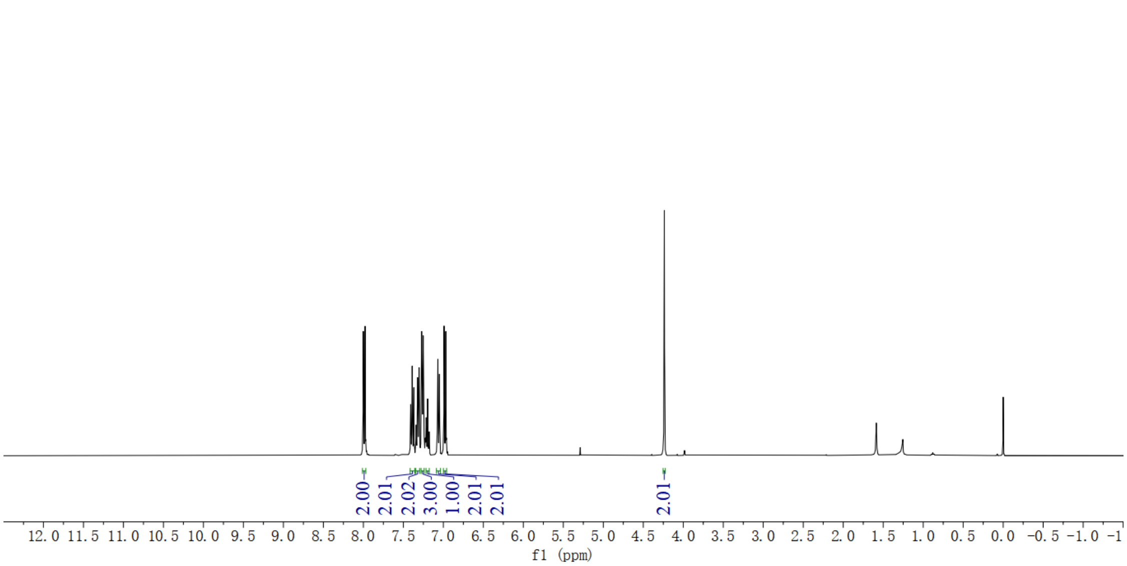

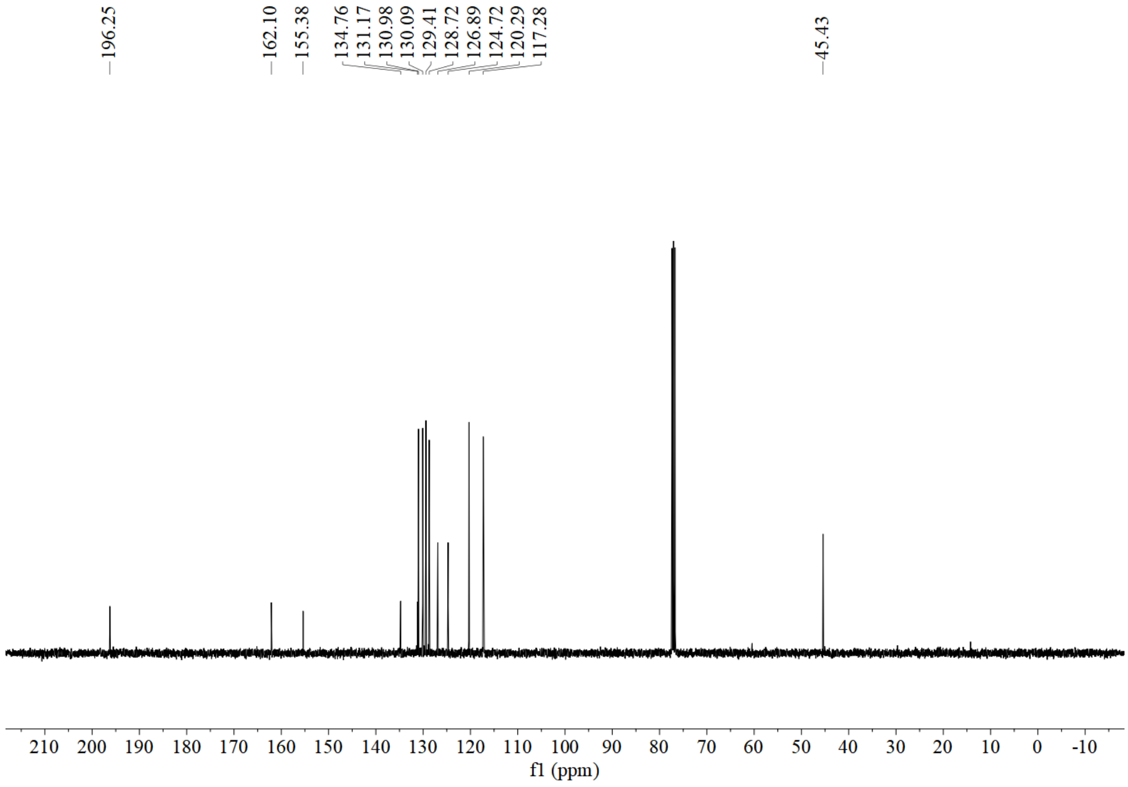

^{1}H NMR (400 MHz) and ^{13}C NMR (101 MHz) in $CDCl_3$ of 2-Phenyl-1-(*o*-tolyl)ethan-1-one (**3t**)

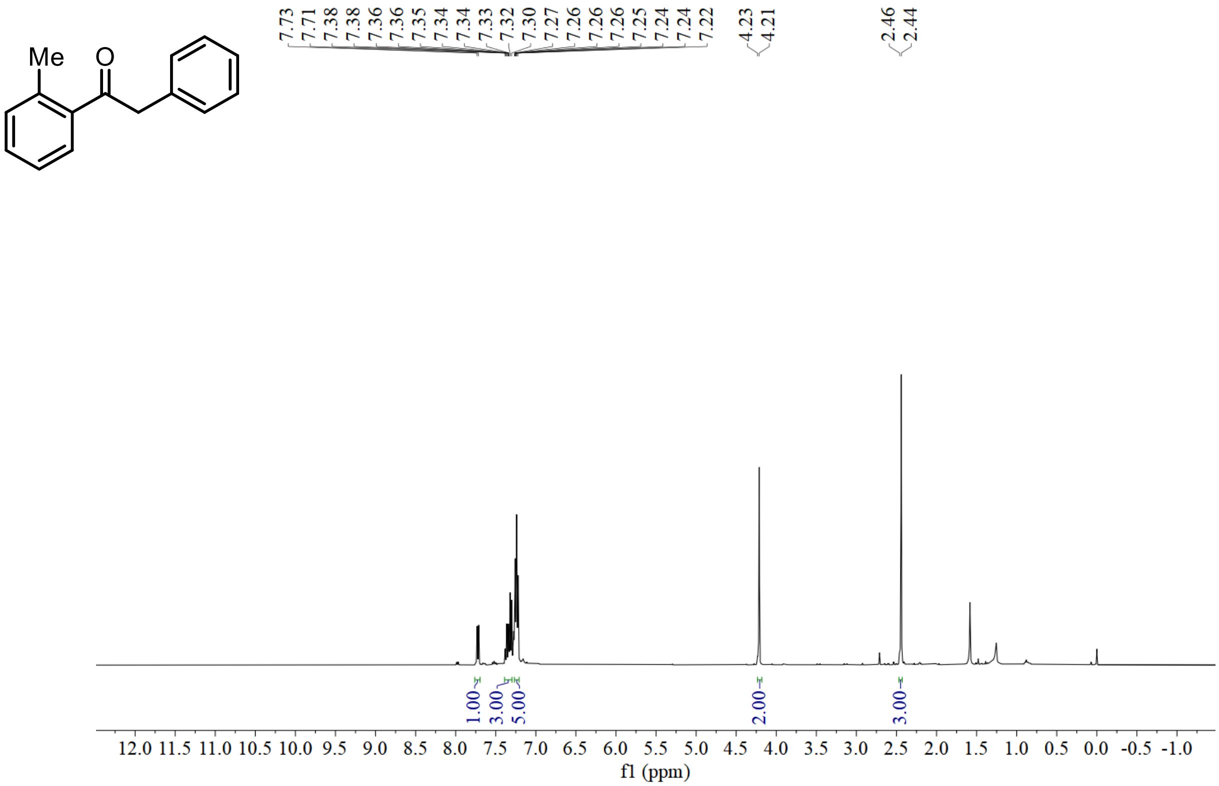

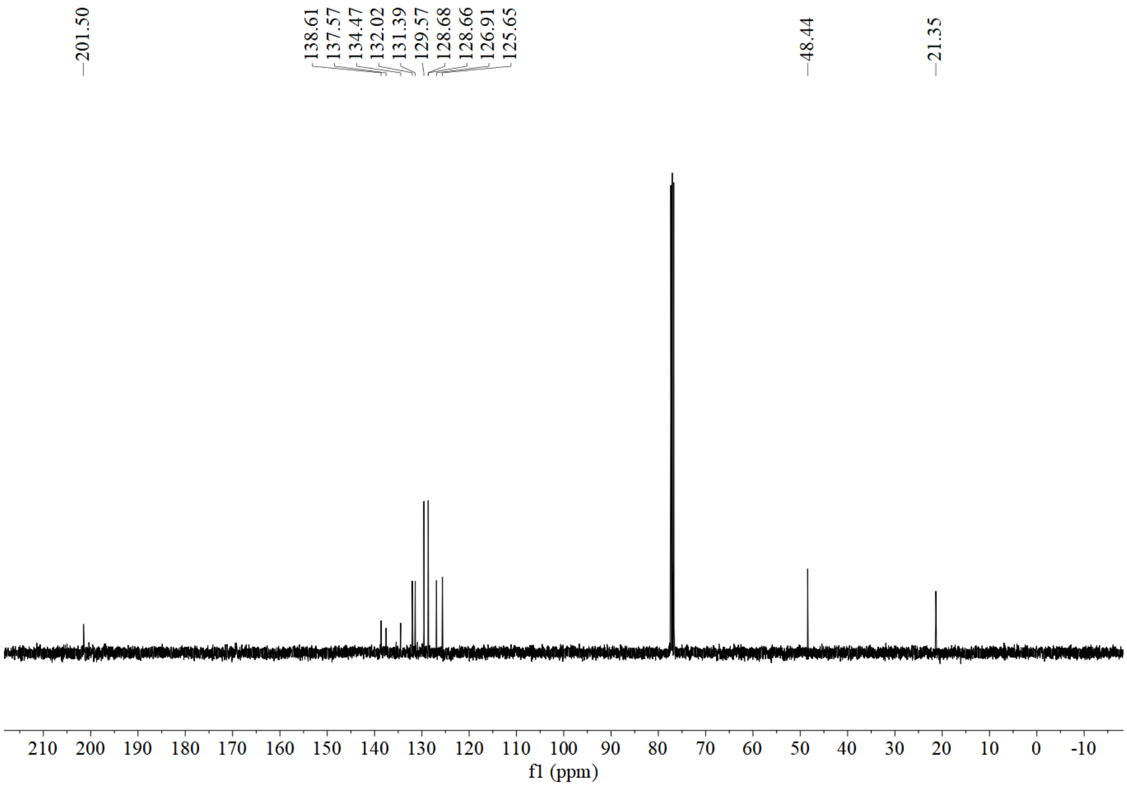

^{1}H NMR (400 MHz) and ^{13}C NMR (101 MHz) in $CDCl_3$ of 2-Phenyl-1-(*m*-tolyl)ethan-1-one (**3u**)

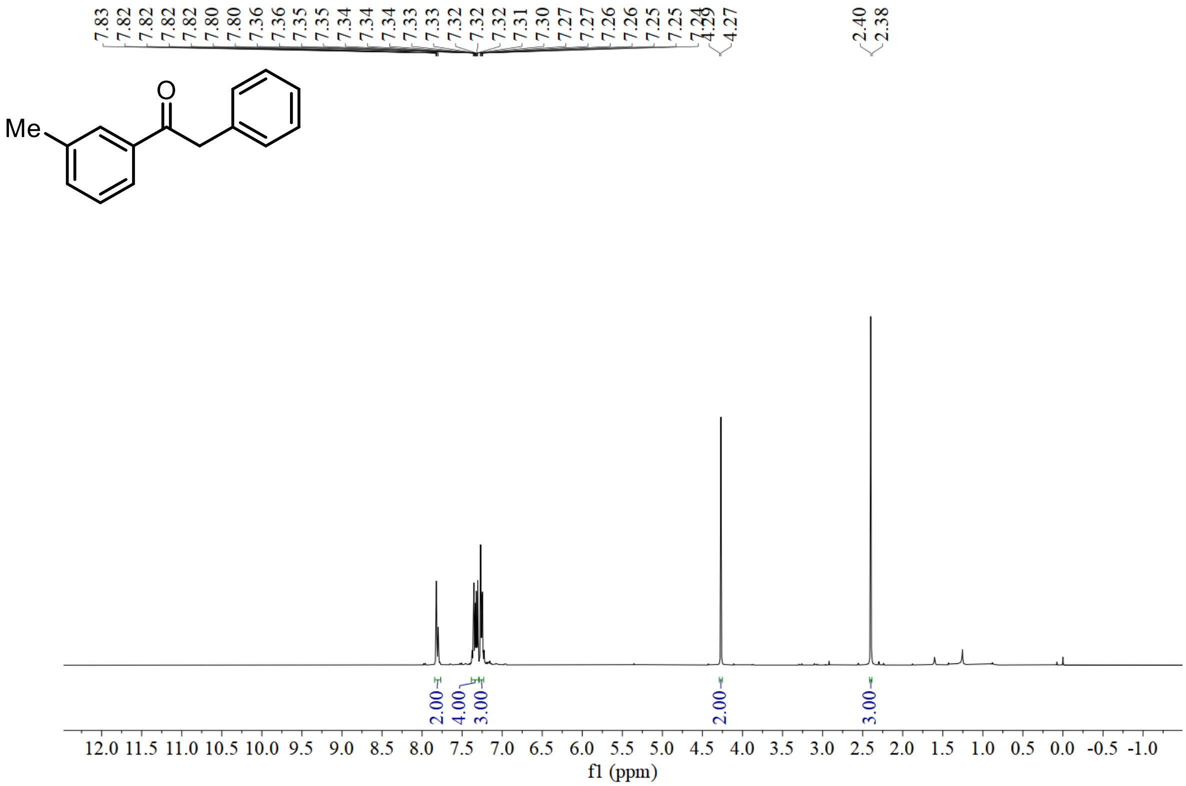

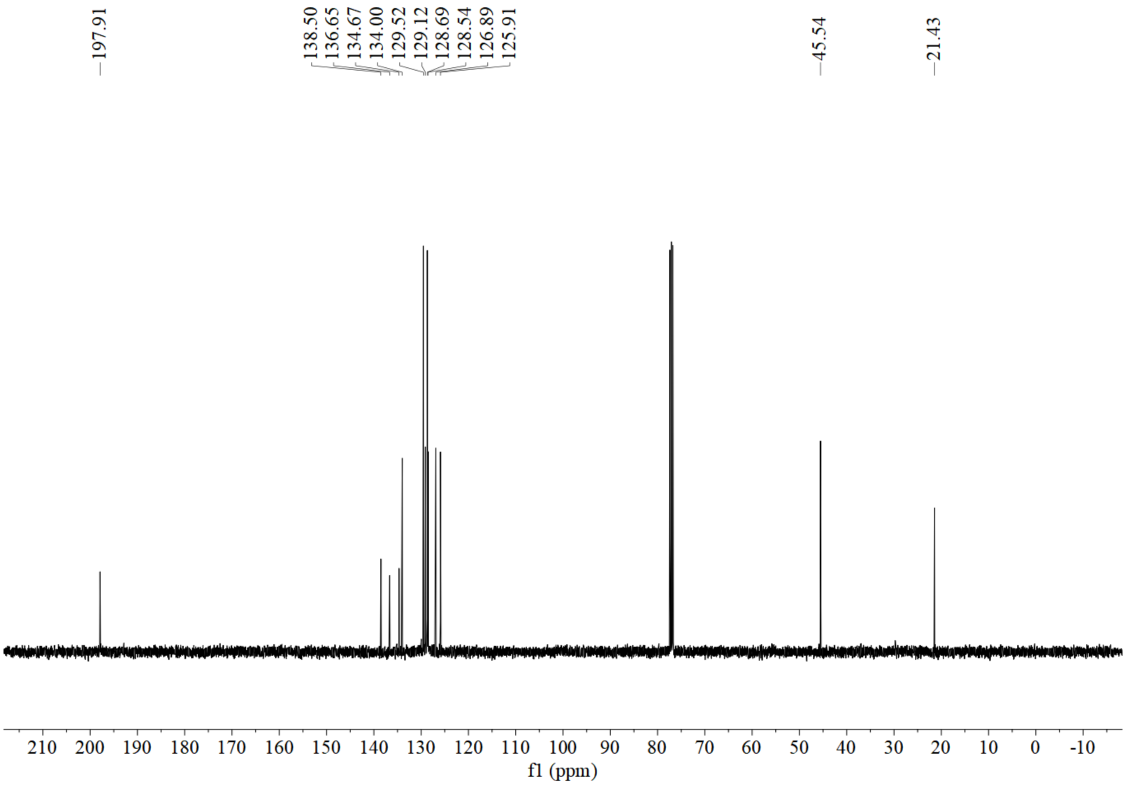

^{1}H NMR (500 MHz) and ^{13}C NMR (126 MHz) in $CDCl_3$ of 1-(2-Methoxyphenyl)-2-phenylethan-1-one (**3v**)

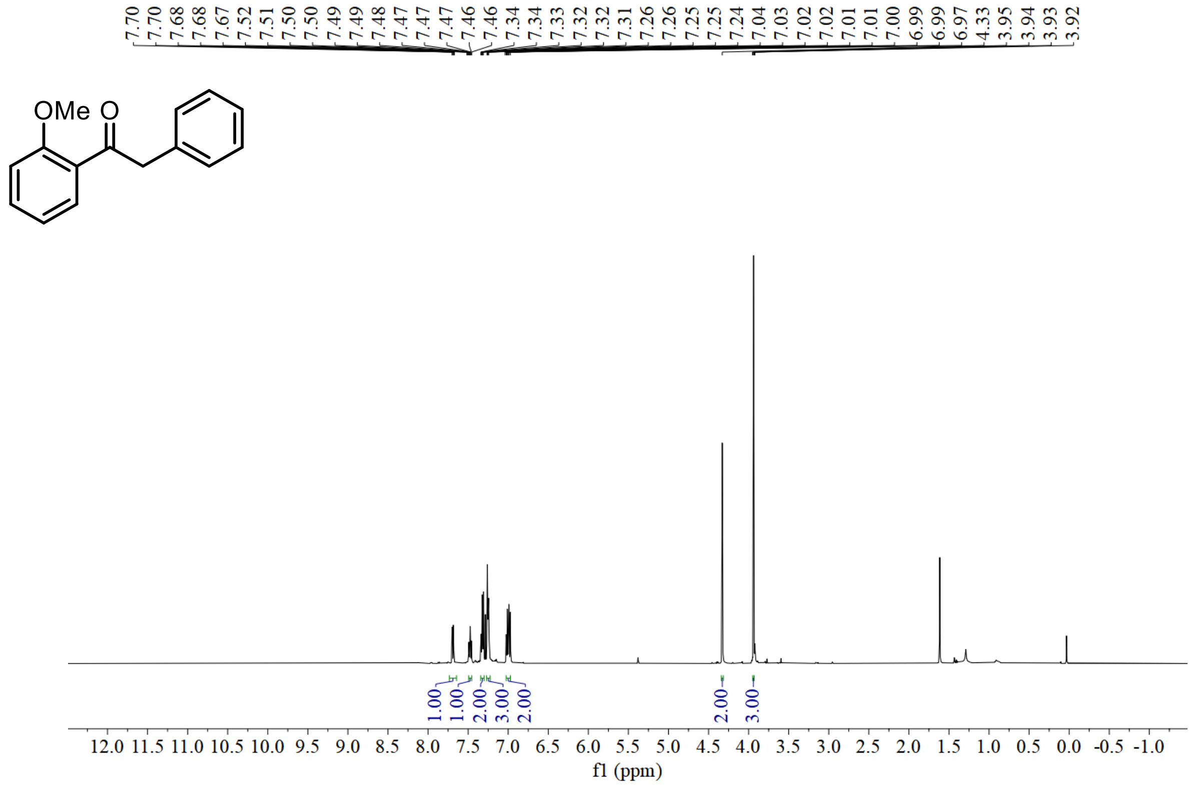

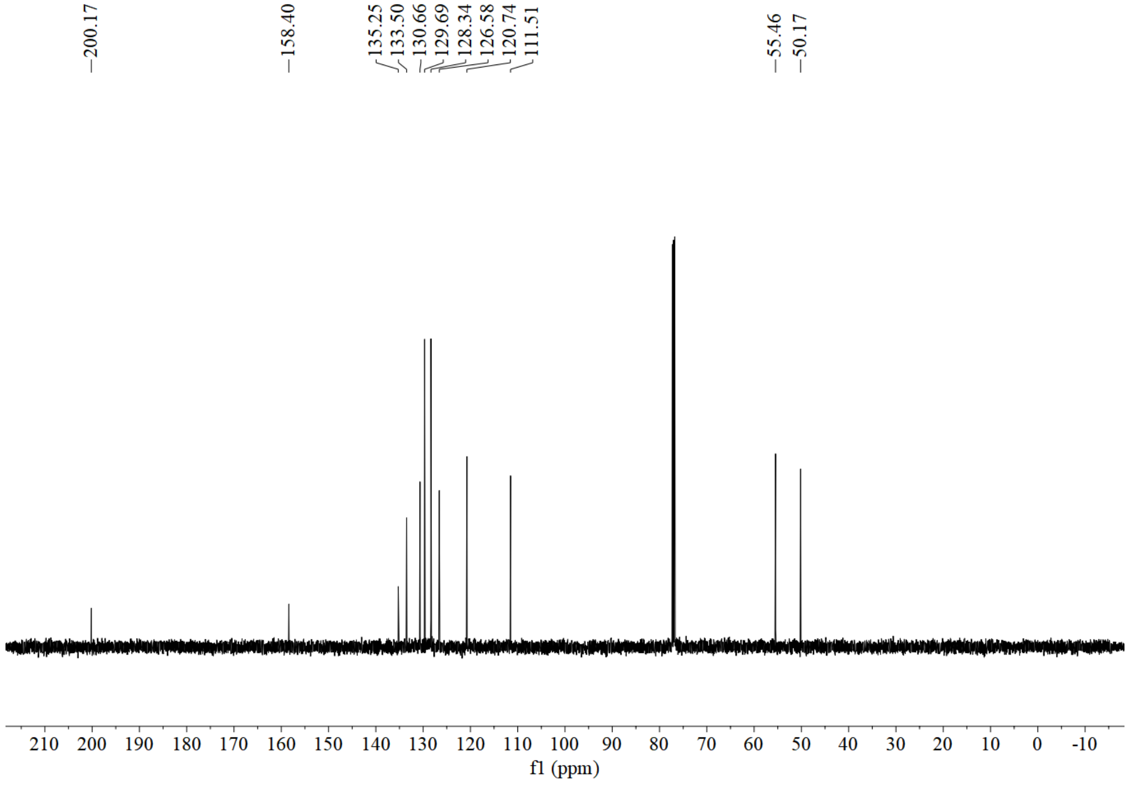

^{1}H NMR (500 MHz) and ^{13}C NMR (126 MHz) in $CDCl_3$ of 1-(3-Methoxyphenyl)-2-phenylethan-1-one (**3w**)

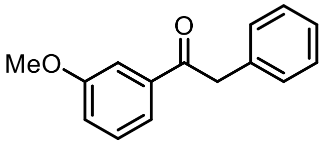

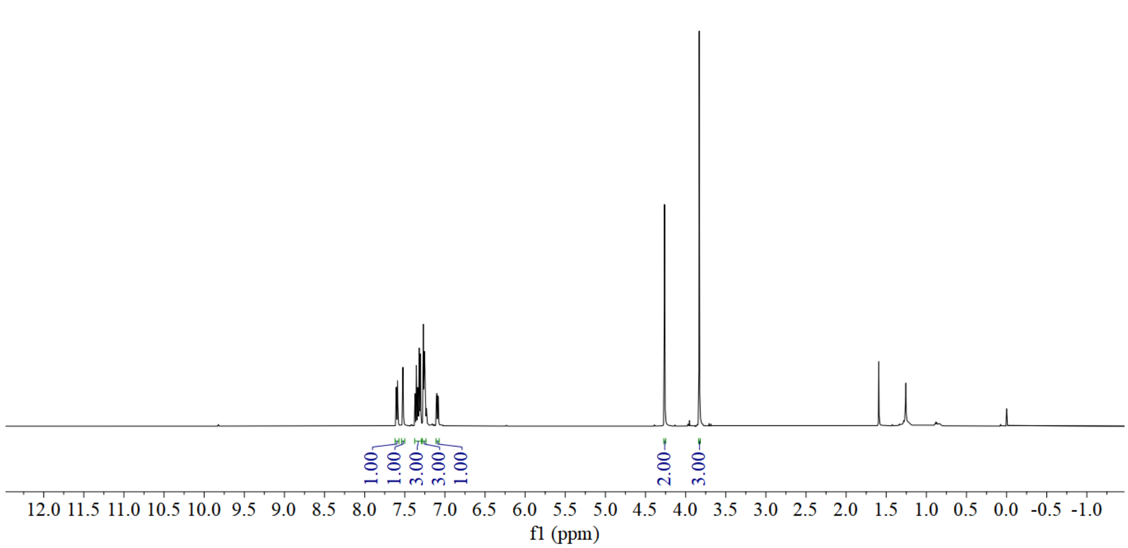

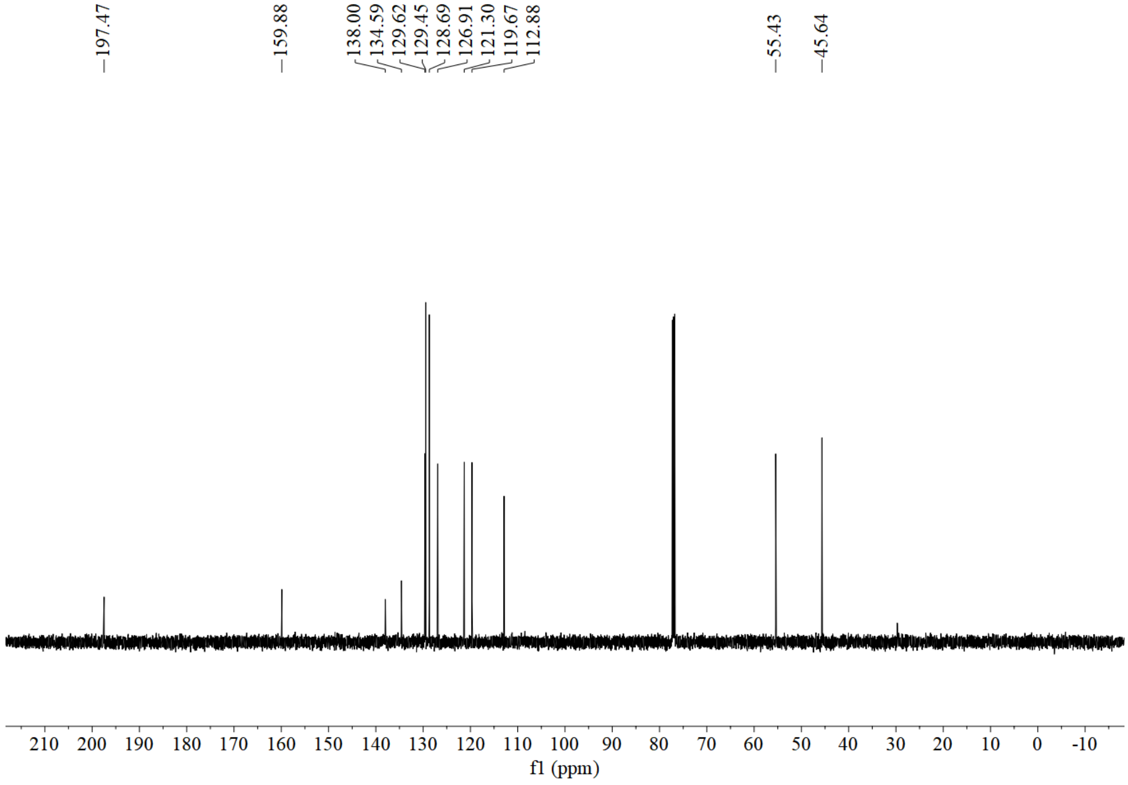

^{1}H NMR (400 MHz), ^{13}C NMR (101 MHz), and ^{19}F NMR (376 MHz) in $CDCl_3$ of 1-(2-Fluorophenyl)-2-phenylethan-1-one (**3x**)

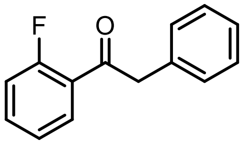

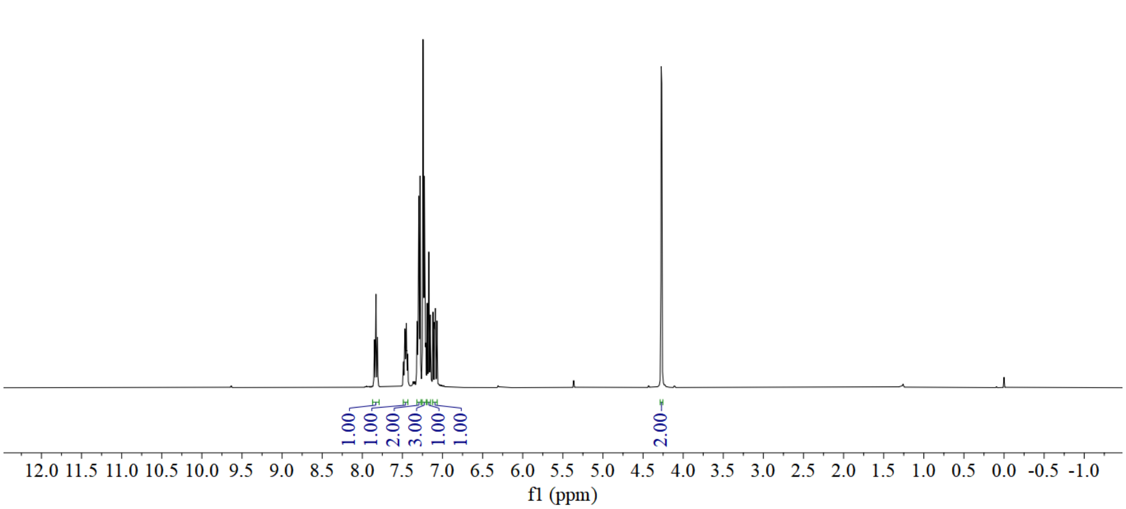

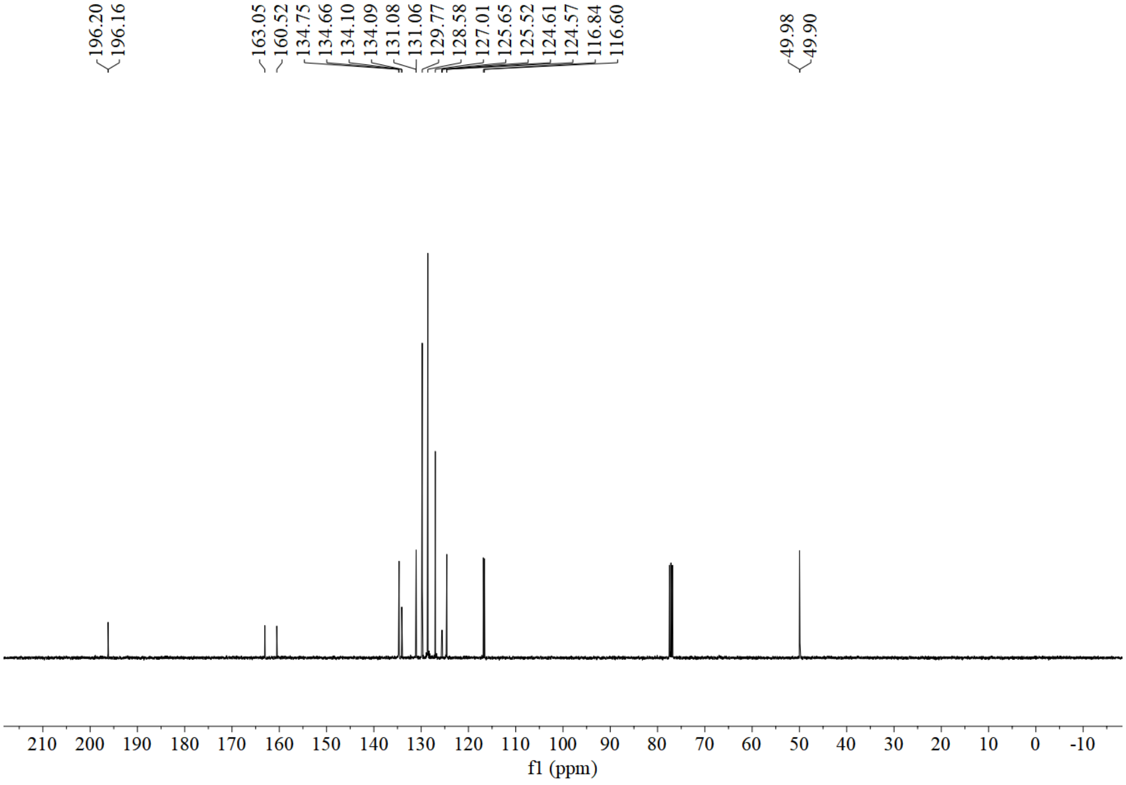

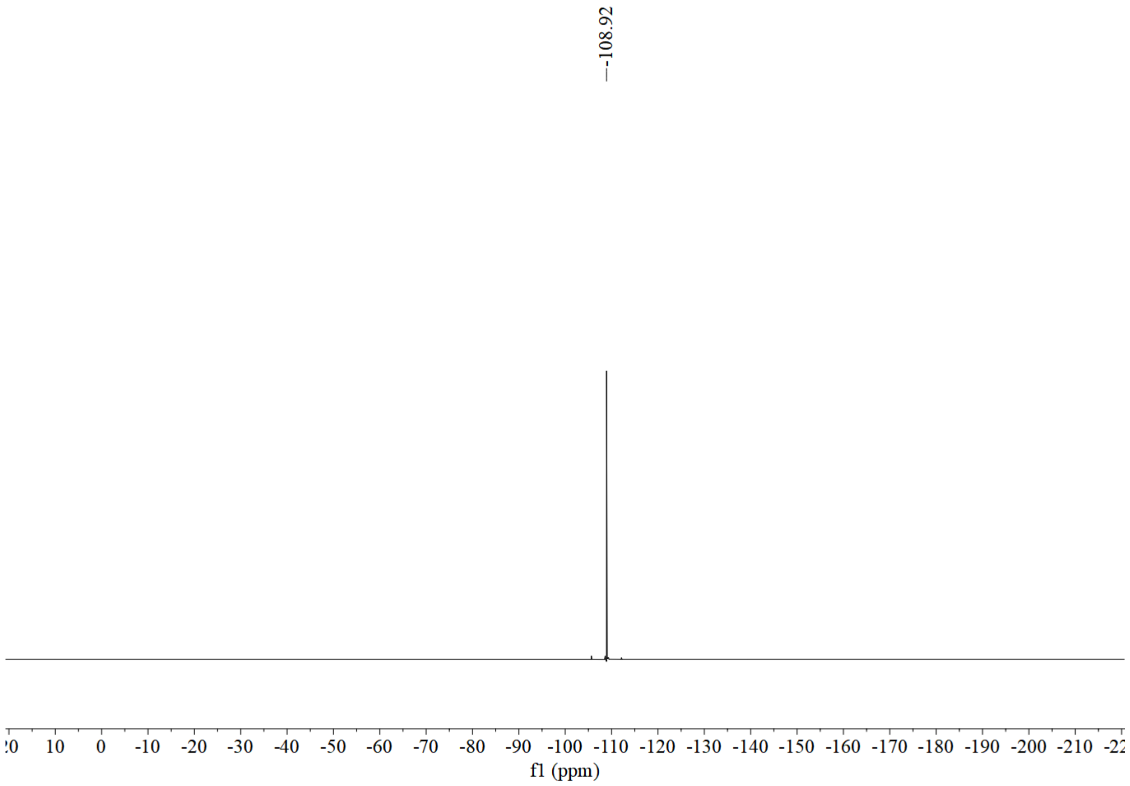
-108.92
20
10
0
-10
-20
-30
-40
-50
-60
-70
-80
-90
-100
-110
-120
-130
-140
-150
-160
-170
-180
-190
-200
-210
f1 (ppm)

^{1}H NMR (400 MHz), ^{13}C NMR (101 MHz), and ^{19}F NMR (376 MHz) in $CDCl_3$ of 1-(3-Fluorophenyl)-2-phenylethan-1-one (**3y**)

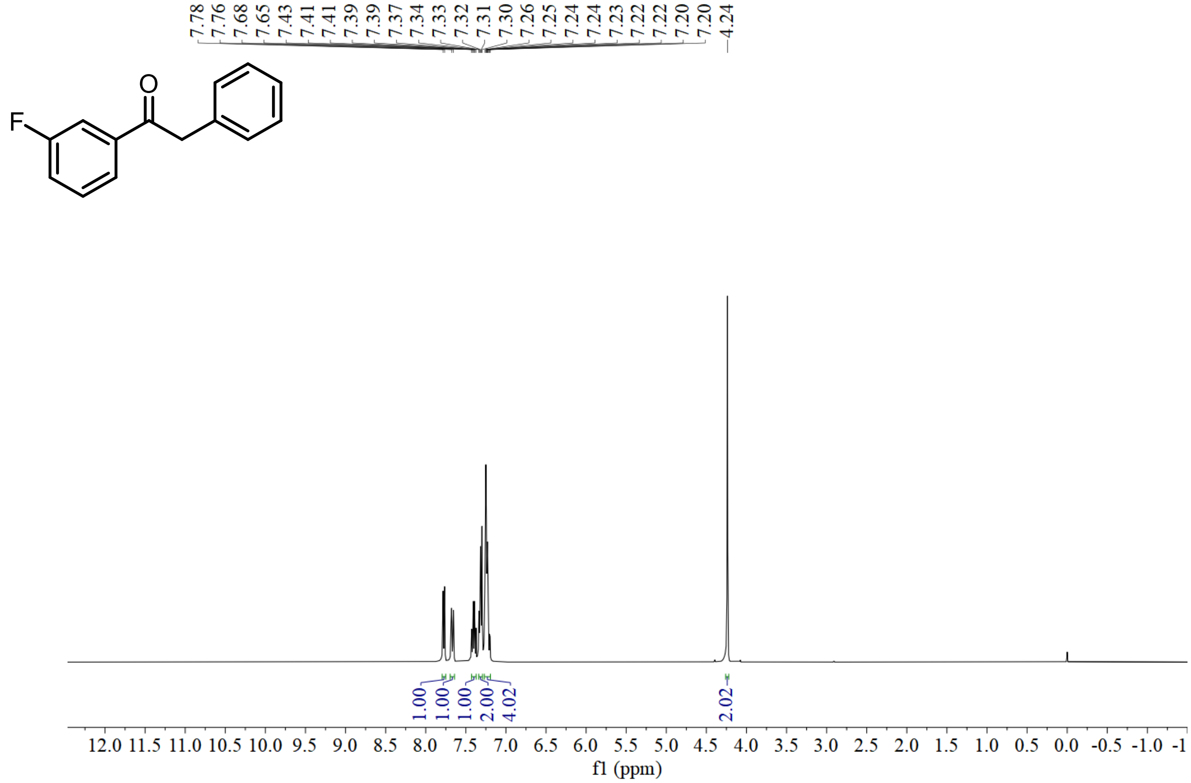

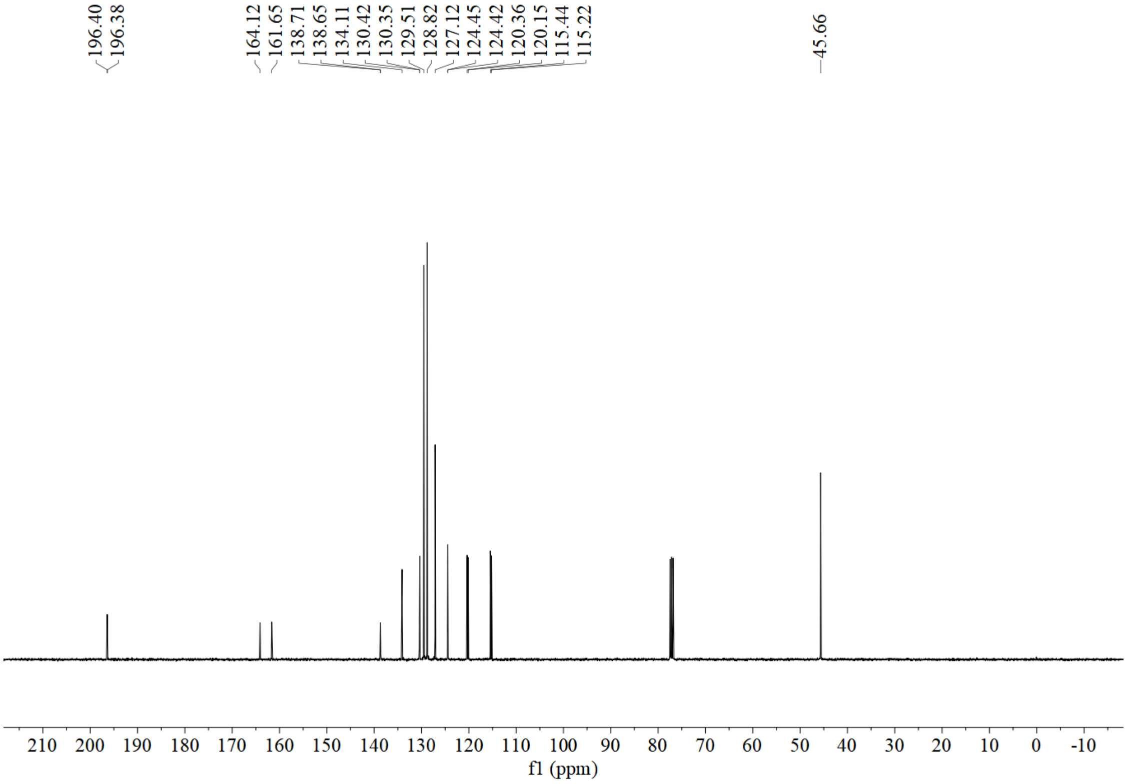

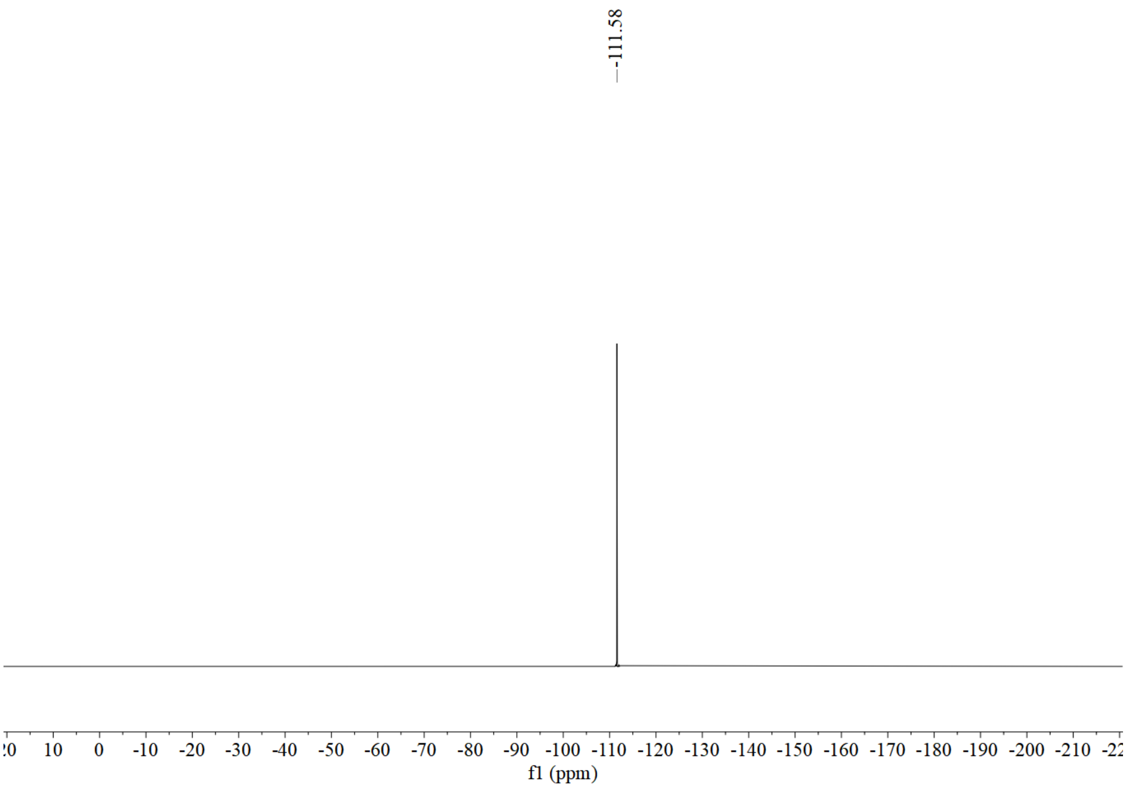
-111.58
20 10 0 -10 -20 -30 -40 -50 -60 -70 -80 -90 -100 -110 -120 -130 -140 -150 -160 -170 -180 -190 -200 -210 -22
f1 (ppm)

^{1}H NMR (500 MHz), ^{13}C NMR (101 MHz), and ^{19}F NMR (376 MHz) in $CDCl_3$ of 2-Phenyl-1-(3-(trifluoromethyl)phenyl)ethan-1-one (**3z**)

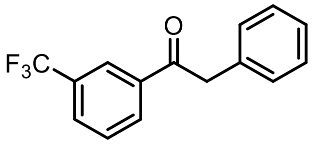

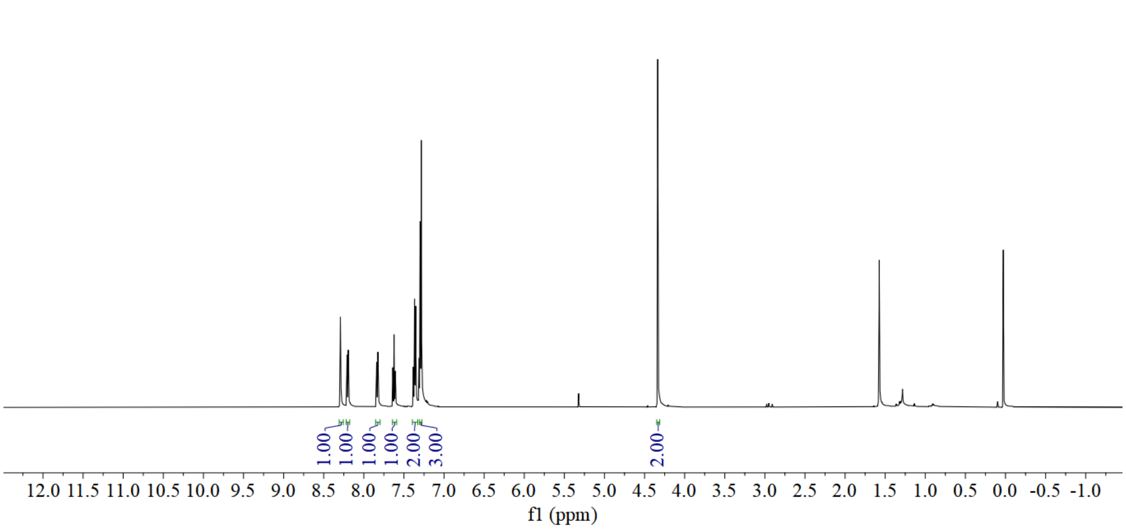

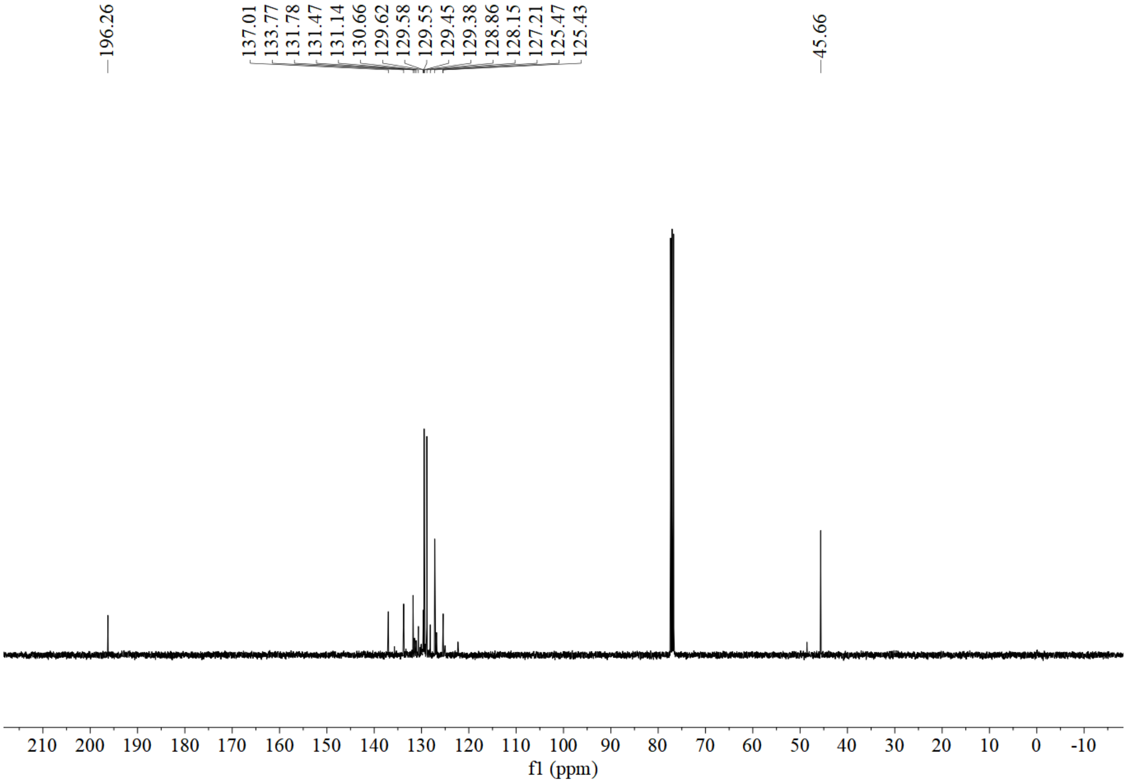

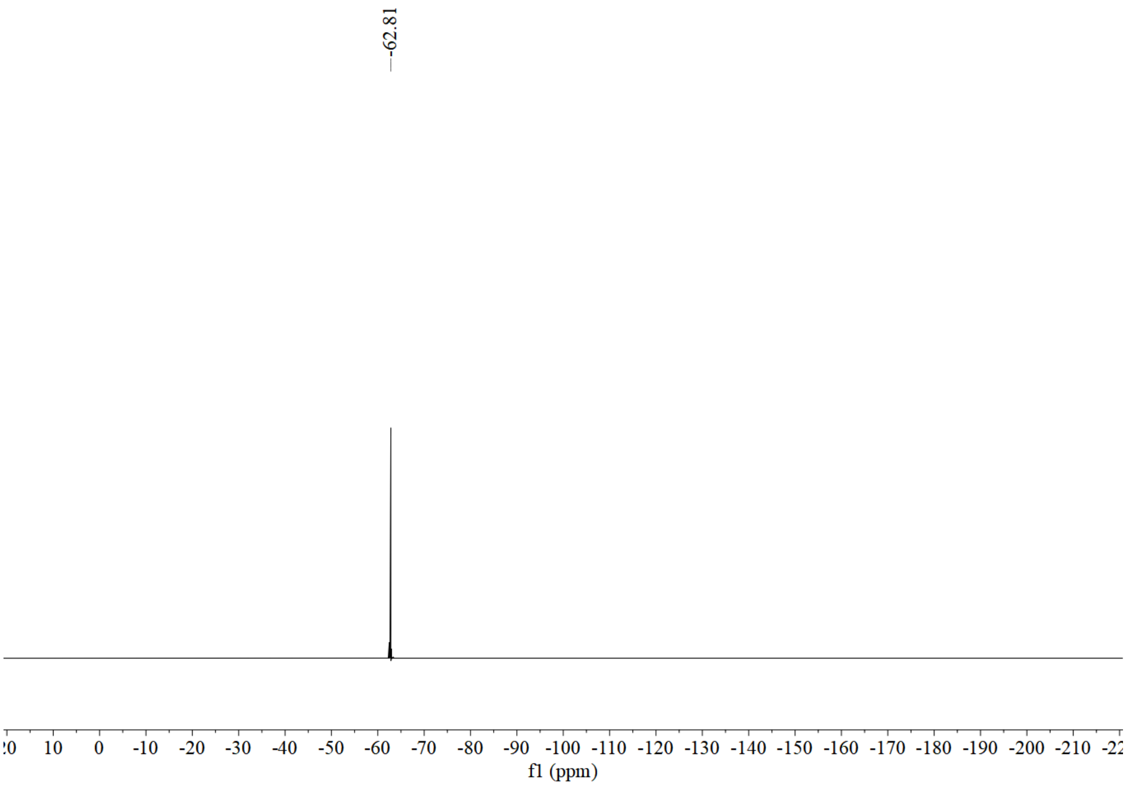
-62.81
20 10 0 -10 -20 -30 -40 -50 -60 -70 -80 -90 -100 -110 -120 -130 -140 -150 -160 -170 -180 -190 -200 -210 -22
f1 (ppm)

^{1}H NMR (400 MHz), ^{13}C NMR (101 MHz), and ^{19}F NMR (376 MHz) in $CDCl_3$ of 1-(3,4-Difluorophenyl)-2-phenylethan-1-one (**3aa**)

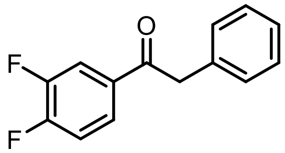

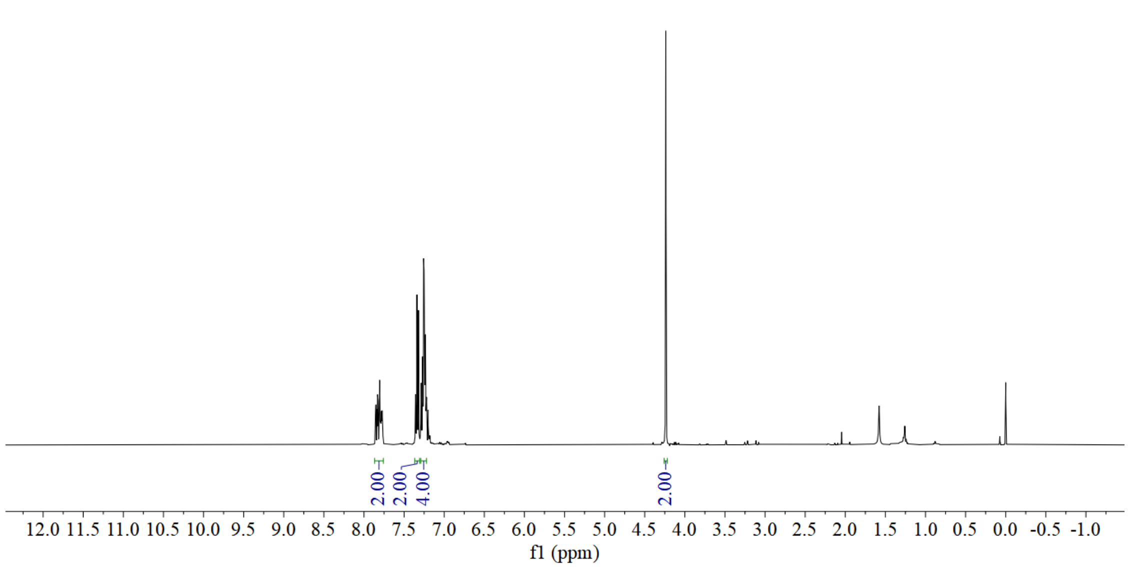

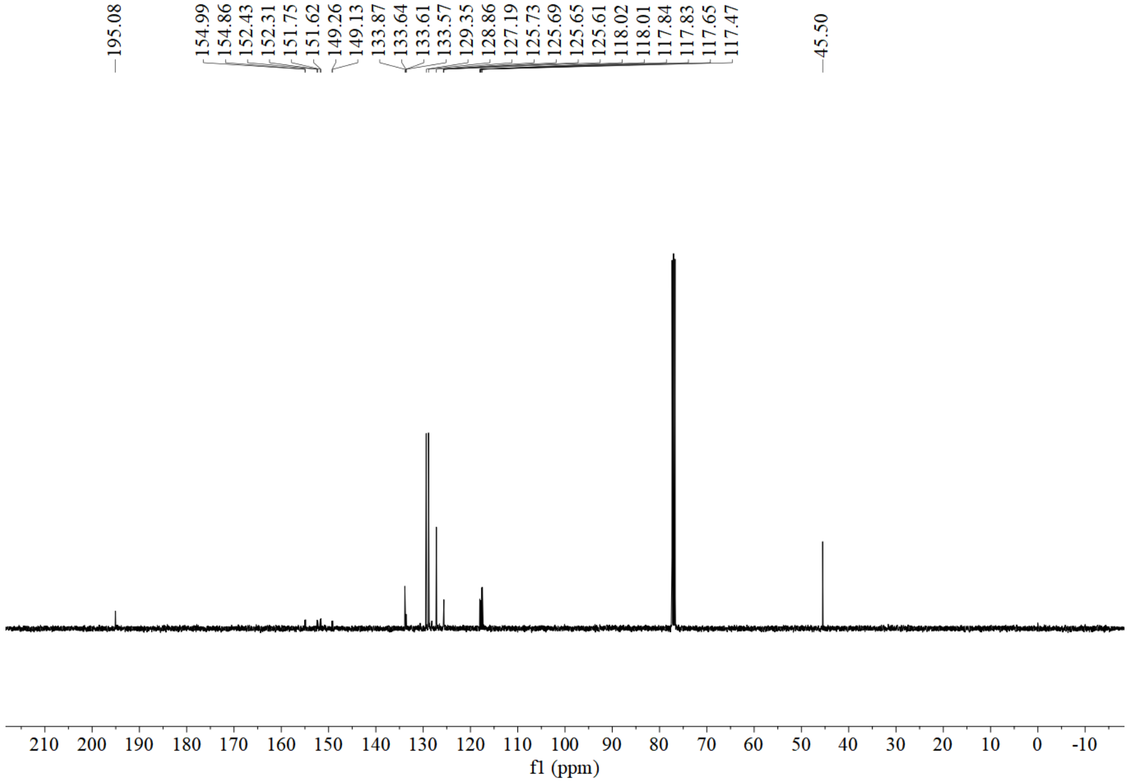

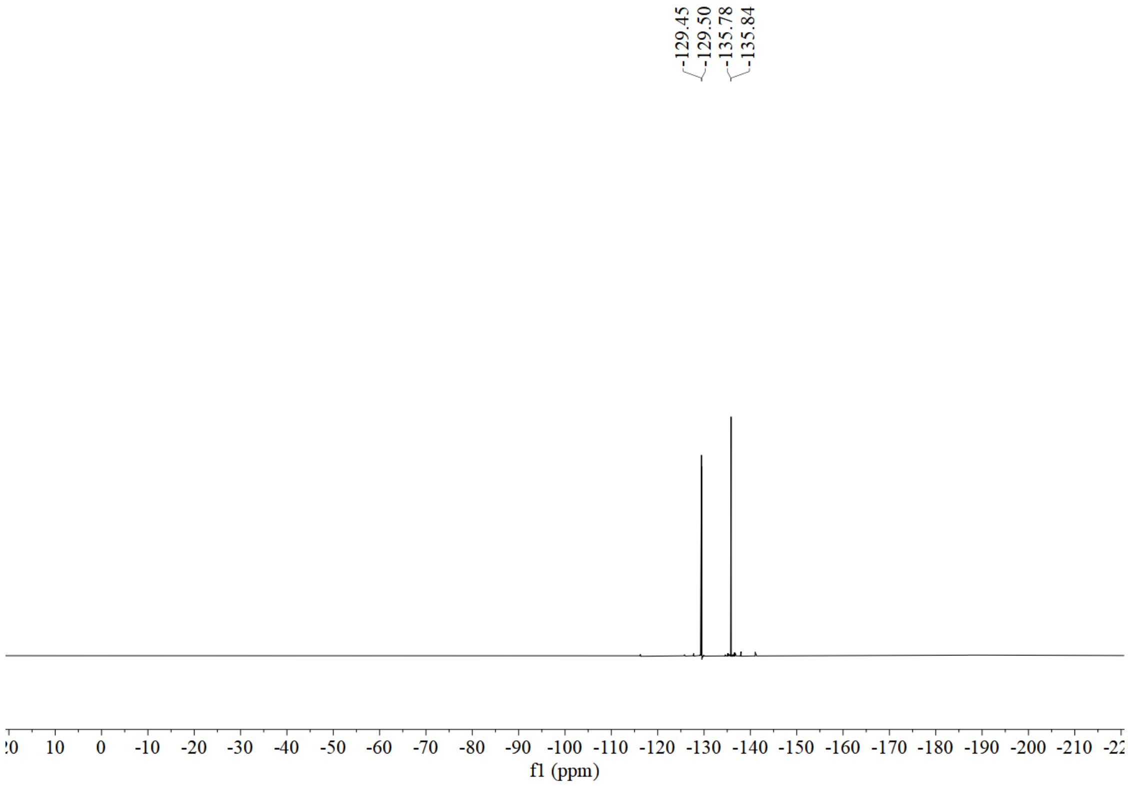
-129.45
-129.50
-135.78
-135.84
20
10
0
-10
-20
-30
-40
-50
-60
-70
-80
-90
-100
-110
-120
-130
-140
-150
-160
-170
-180
-190
-200
-210
-22
f1 (ppm)

^{1}H NMR (400 MHz) and ^{13}C NMR (101 MHz) in $CDCl_3$ of 1-(3,4-Dimethoxyphenyl)-2-phenylethan-1-one (**3ab**)

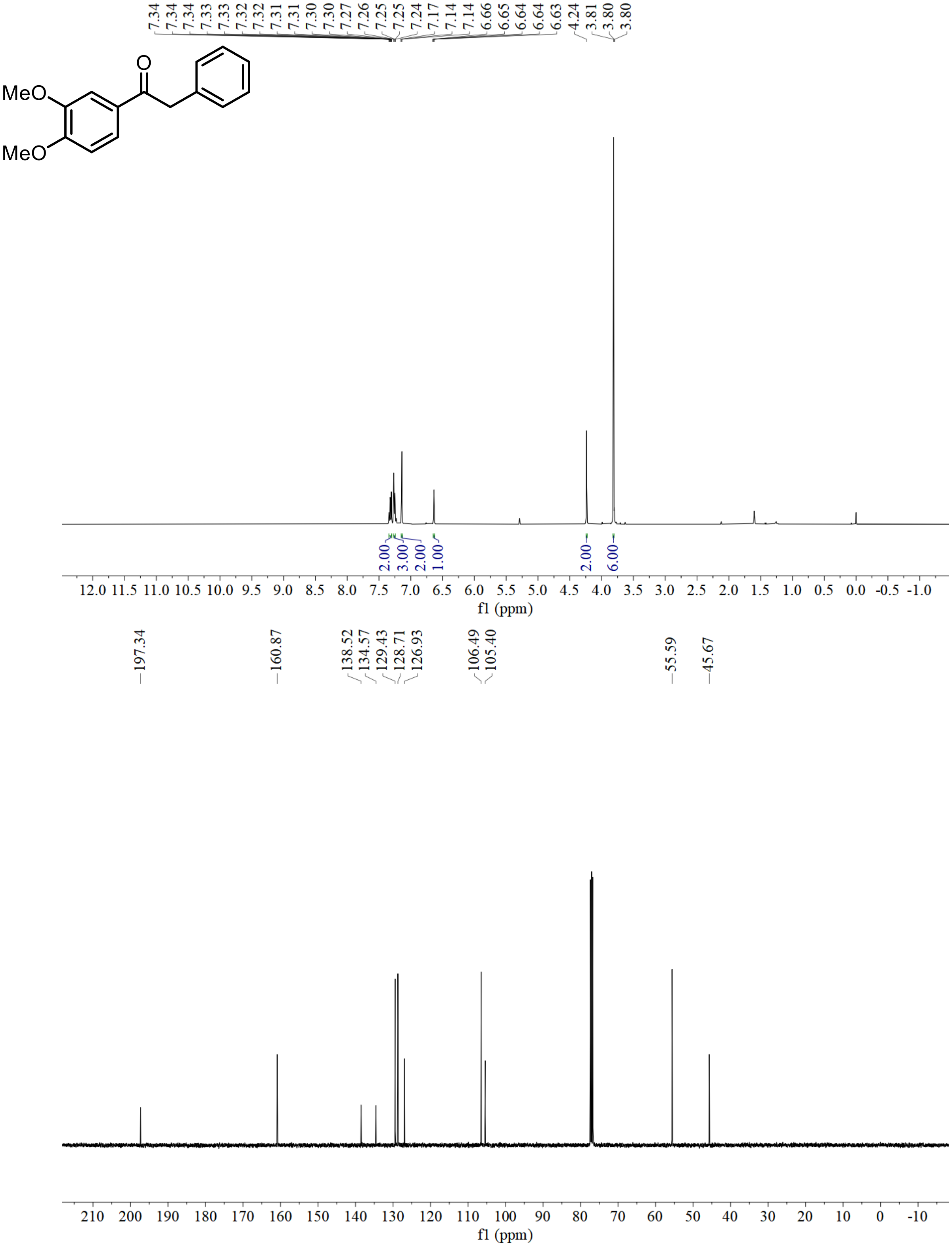

^{1}H NMR (400 MHz) and ^{13}C NMR (101 MHz) in $CDCl_3$ of 1-(Benzo[*d*][1,3]dioxol-5-yl)-2-phenylethan-1-one (**3ac**)

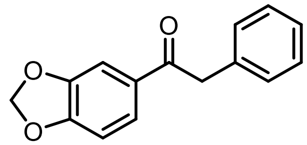

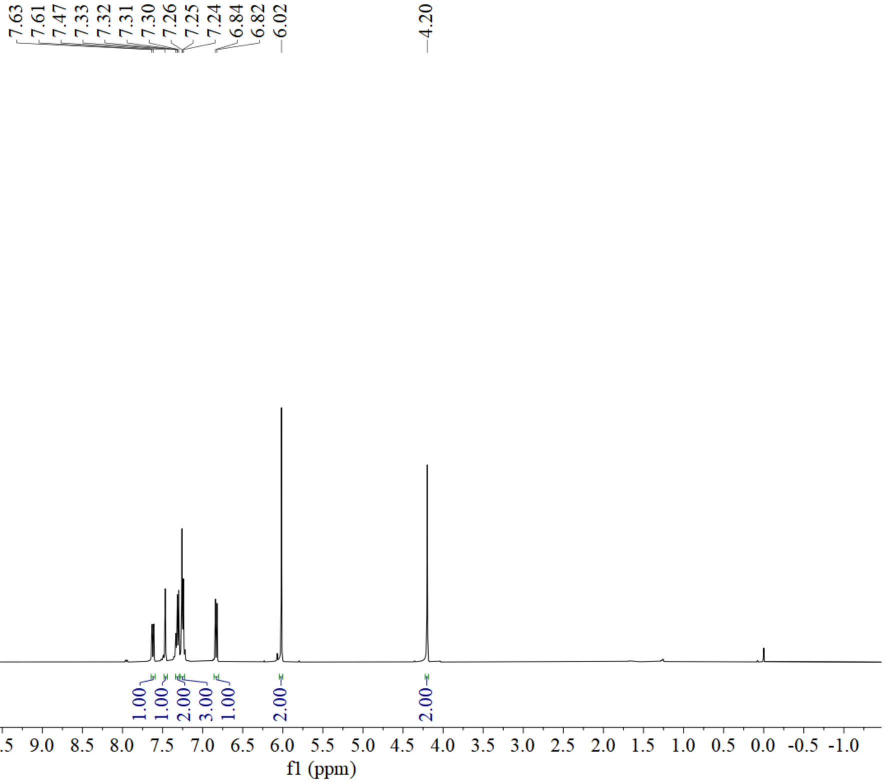

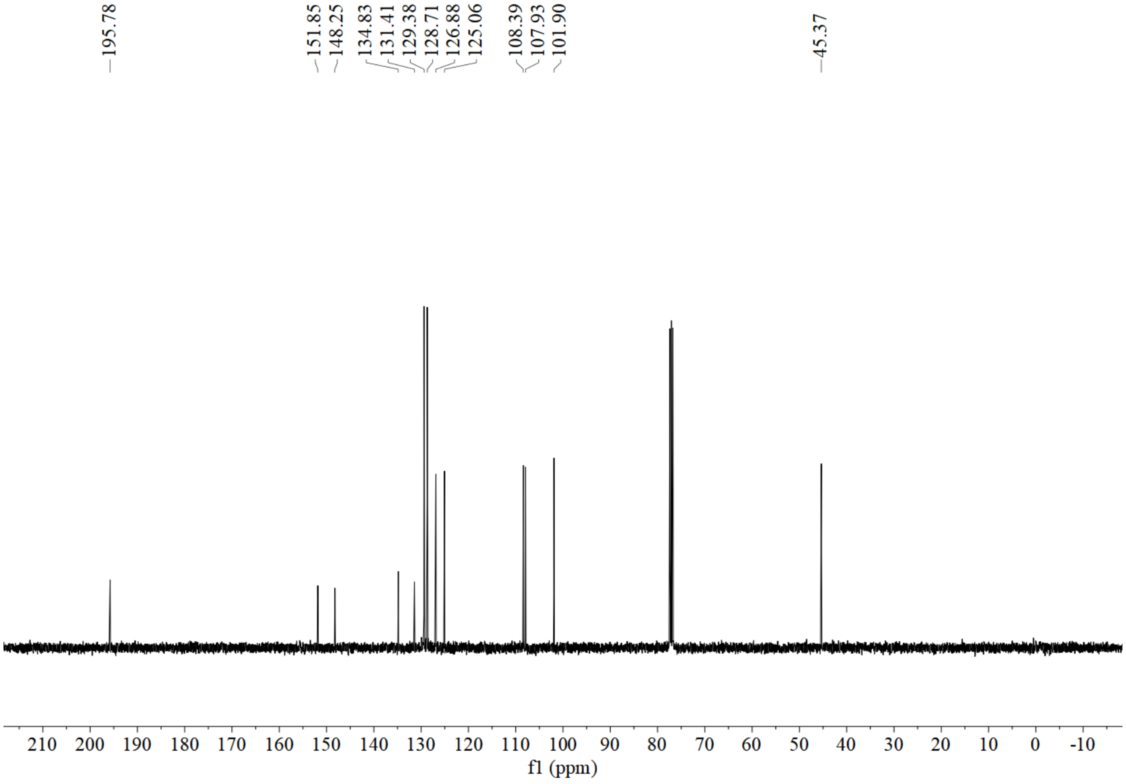

^{1}H NMR (400 MHz) and ^{13}C NMR (101 MHz) in $CDCl_3$ of 2-Phenyl-1-(thiophen-3-yl)ethan-1-one (**3ad**)

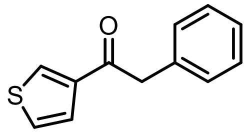

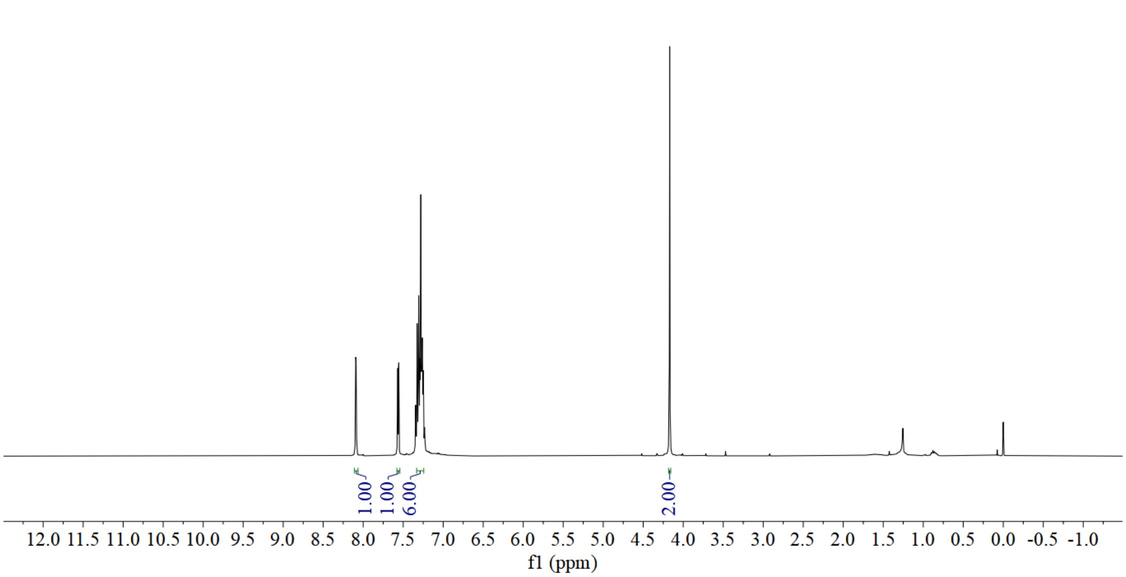

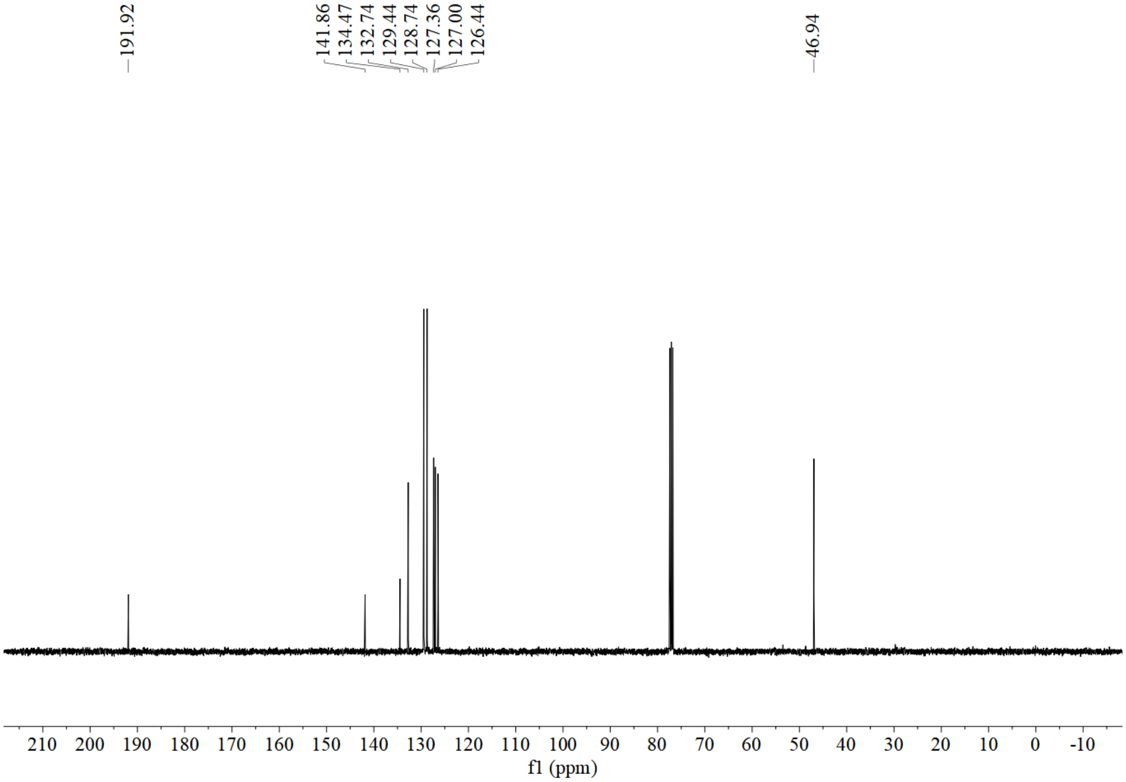

^{1}H NMR (400 MHz) and ^{13}C NMR (101 MHz) in $CDCl_3$ of 2-Phenyl-1-(thiophen-2-yl)ethan-1-one (**3ae**)

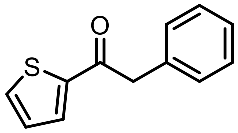

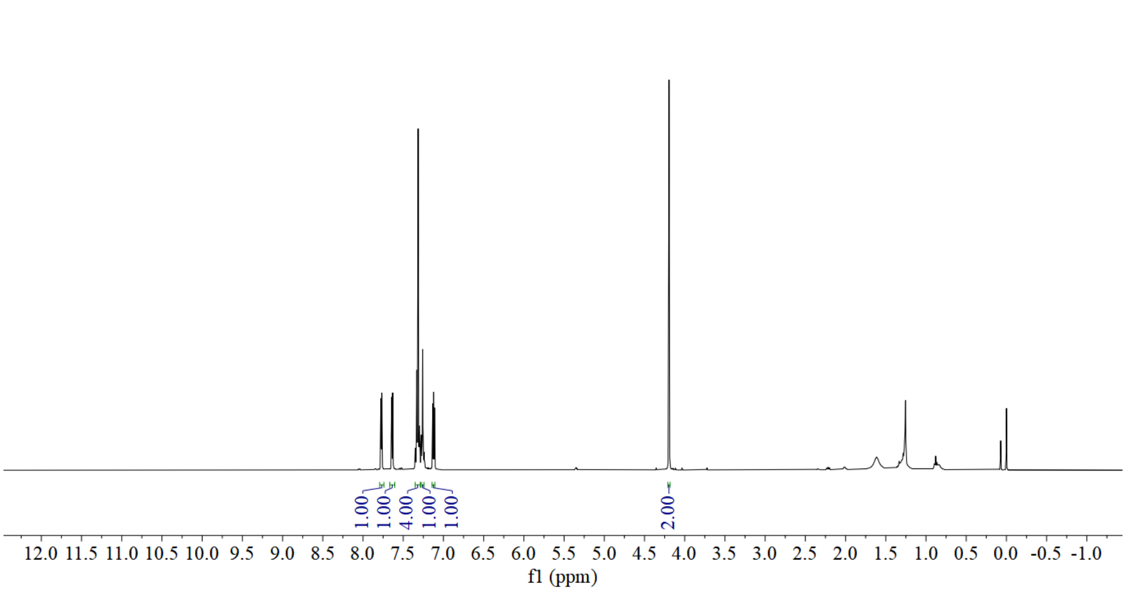

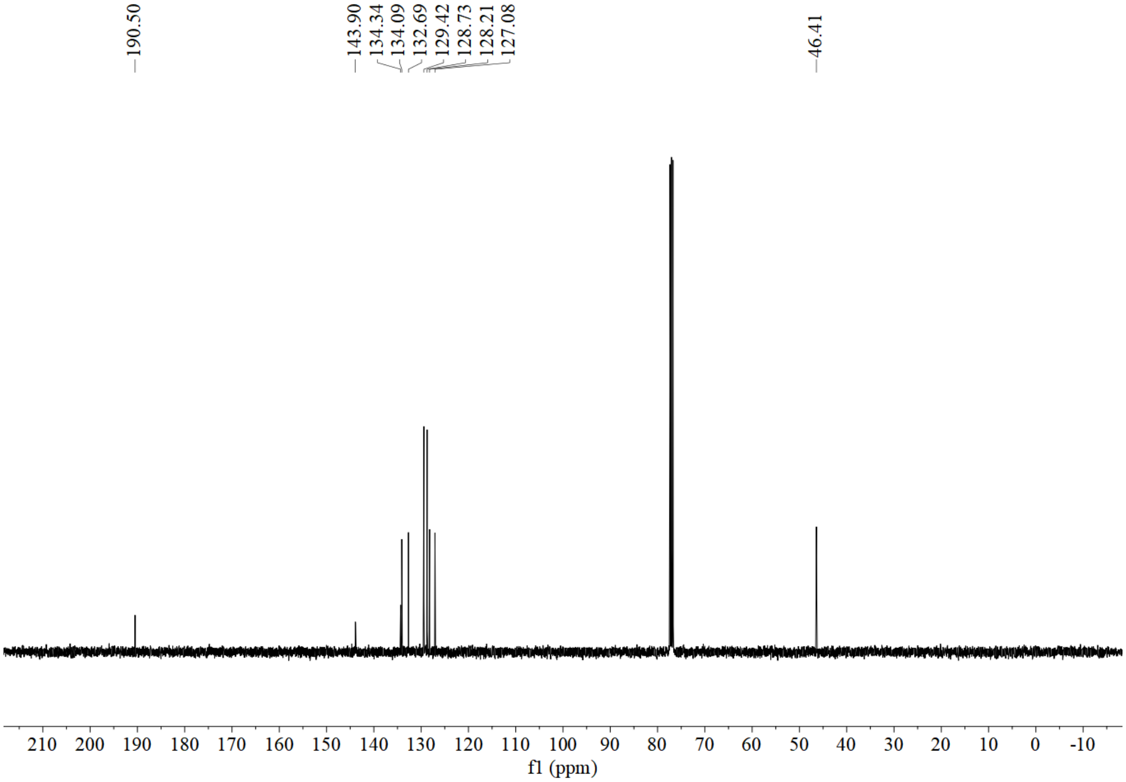

^{1}H NMR (400 MHz) and ^{13}C NMR (126 MHz) in $CDCl_3$ of 1-(Furan-2-yl)-2-phenylethan-1-one (**3af**)

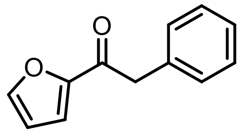

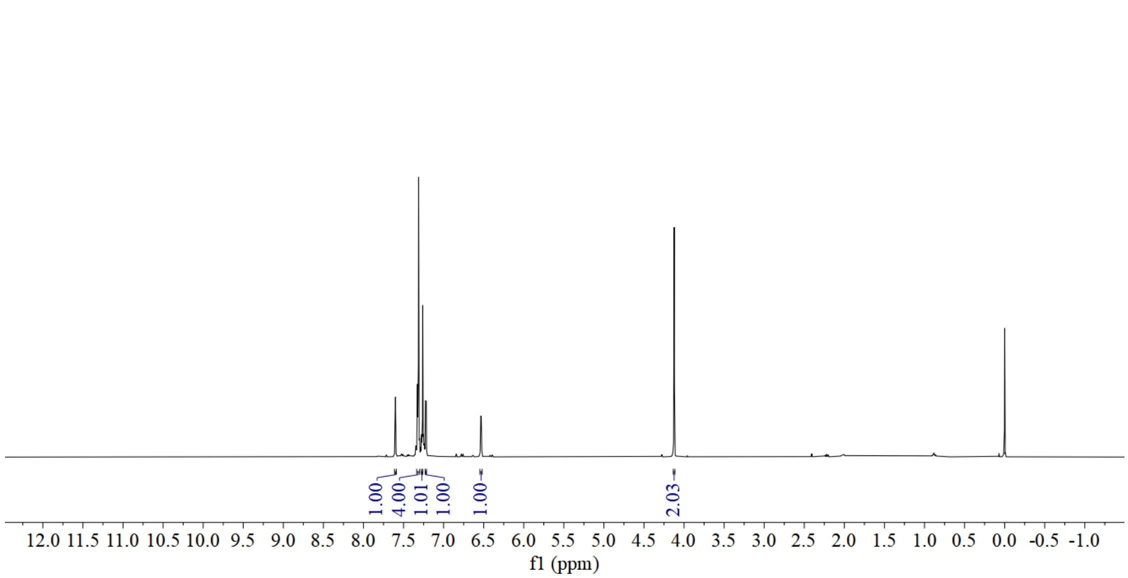

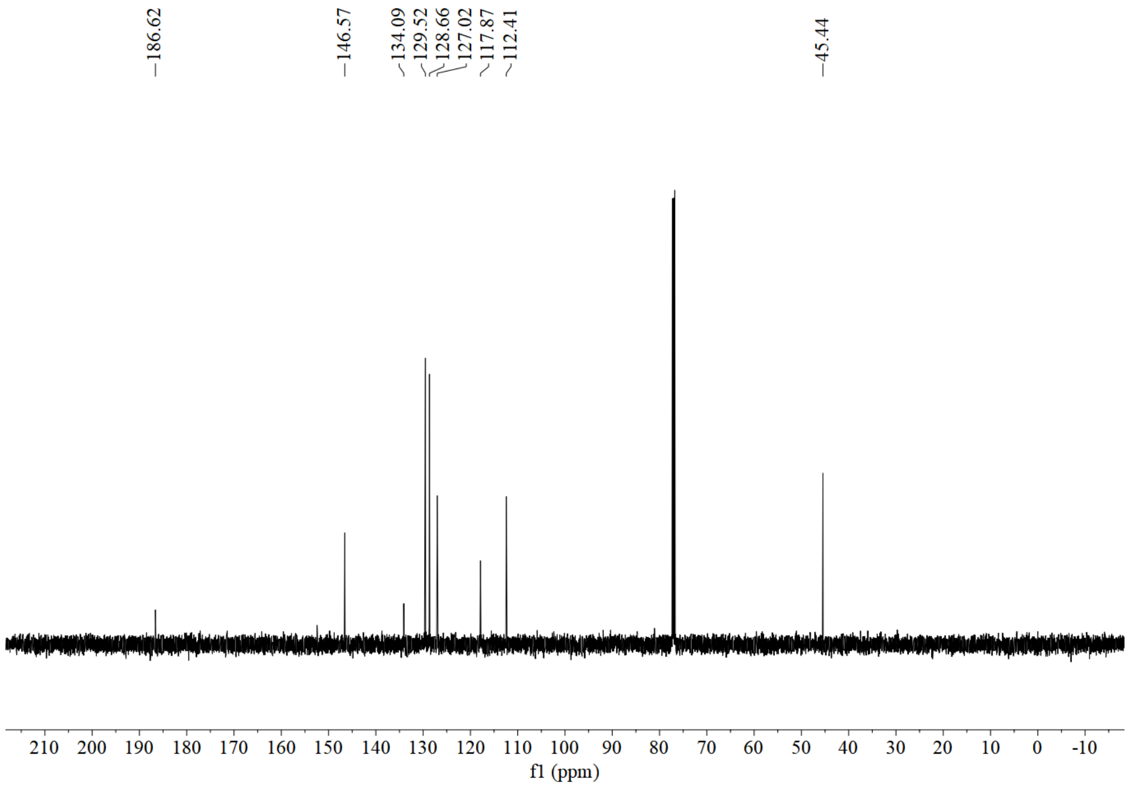

^{1}H NMR (500 MHz) and ^{13}C NMR (126 MHz) in $CDCl_3$ of 1-(Naphthalen-2-yl)-2-phenylethan-1-one (**3ag**)

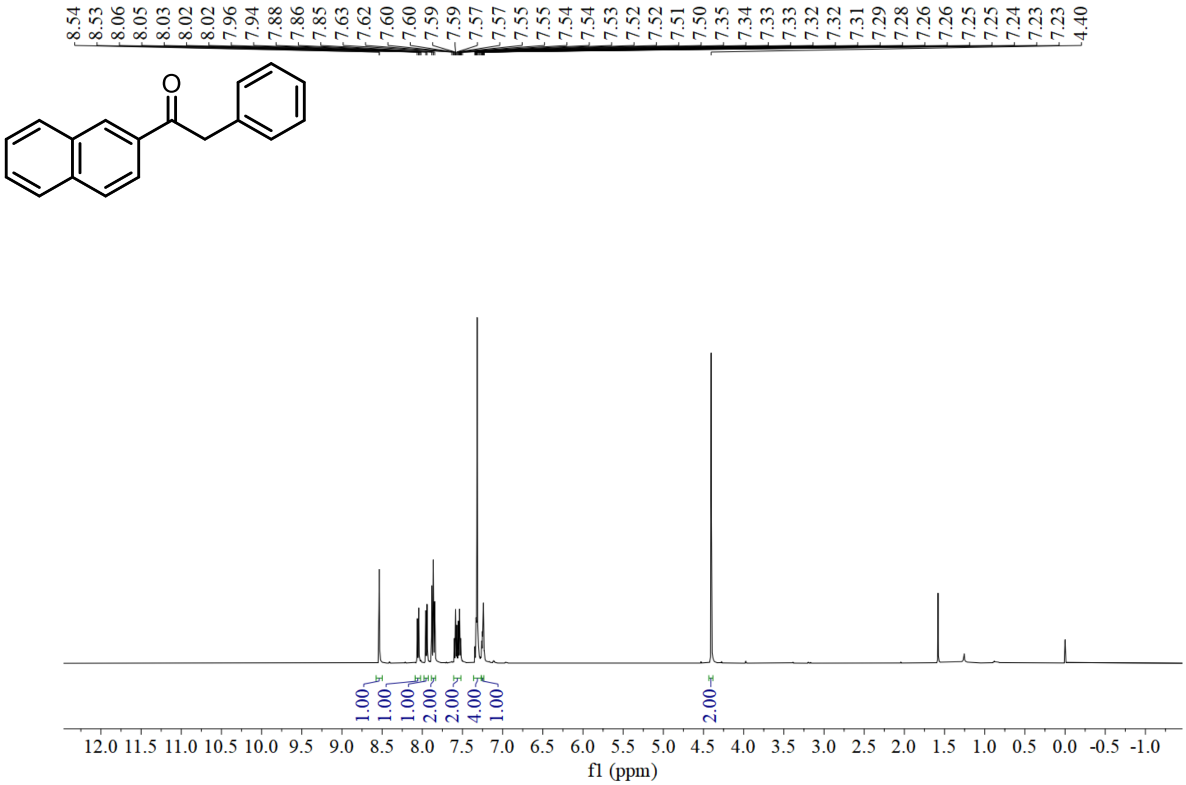

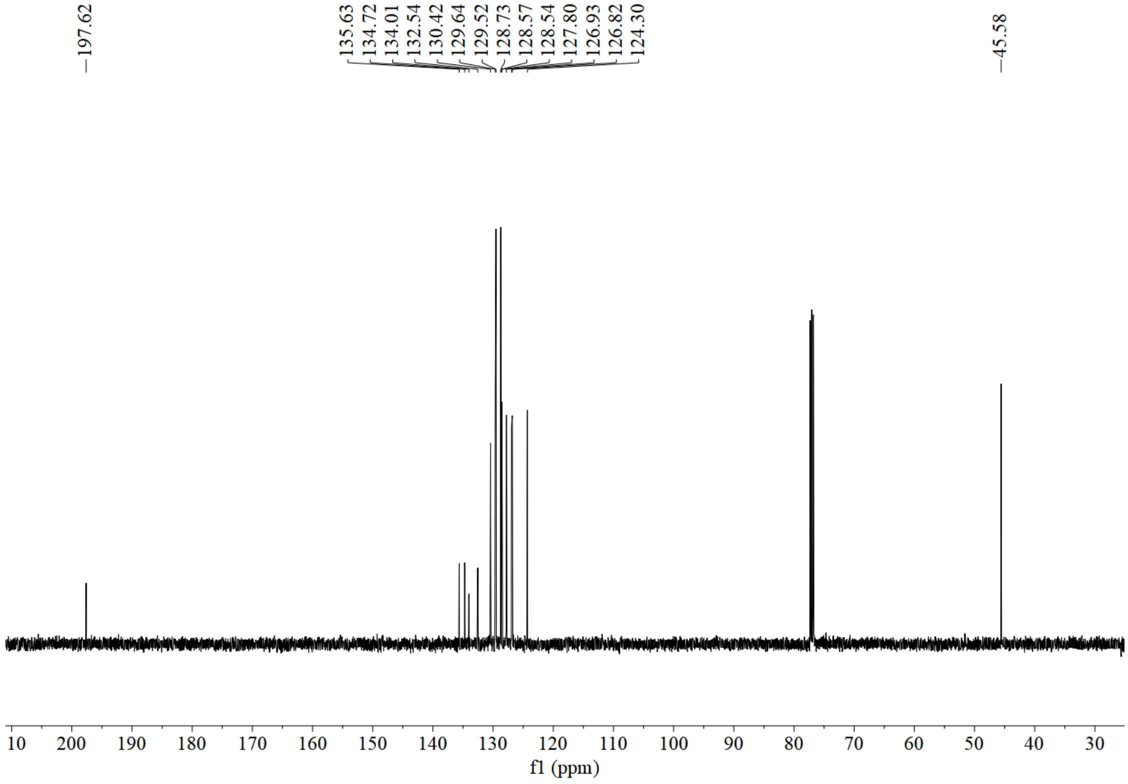

^{1}H NMR (400 MHz), ^{13}C NMR (101 MHz), and ^{19}F NMR (376 MHz) in $CDCl_3$ of Cyclopentyl(4-(trifluoromethyl)phenyl)methanone (**3ah**)

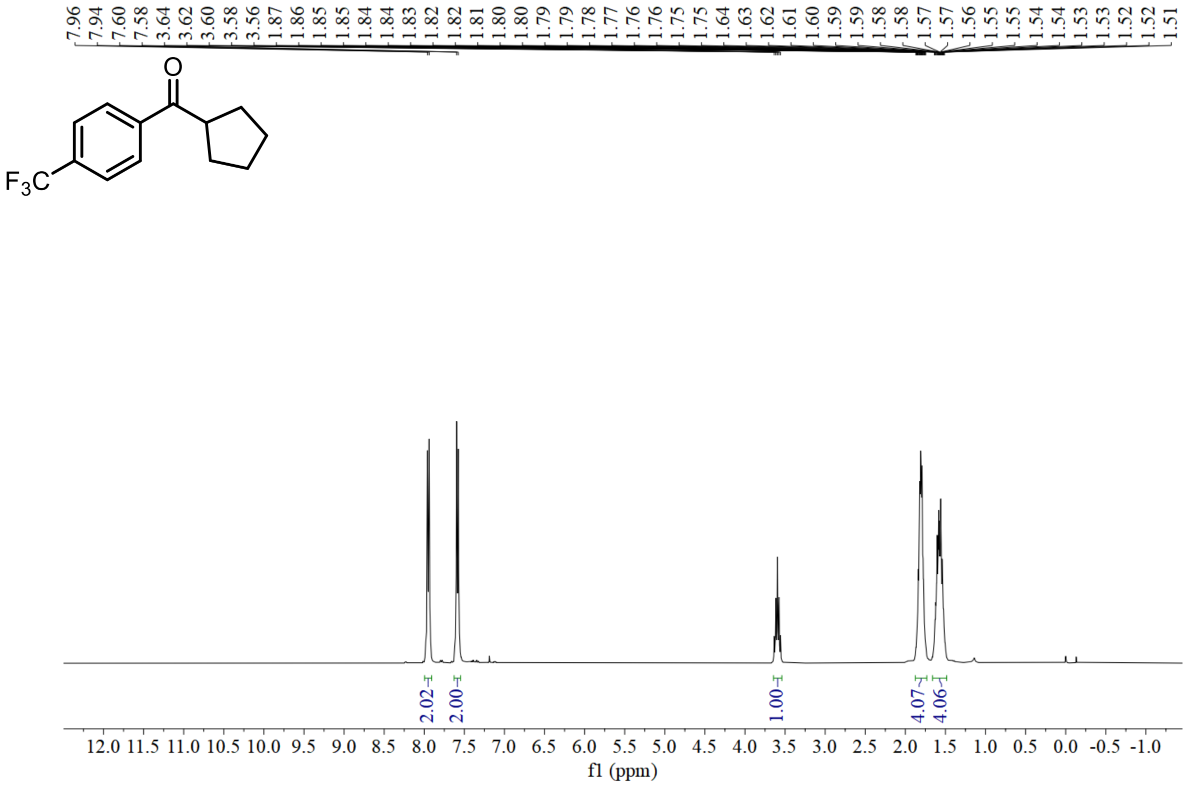

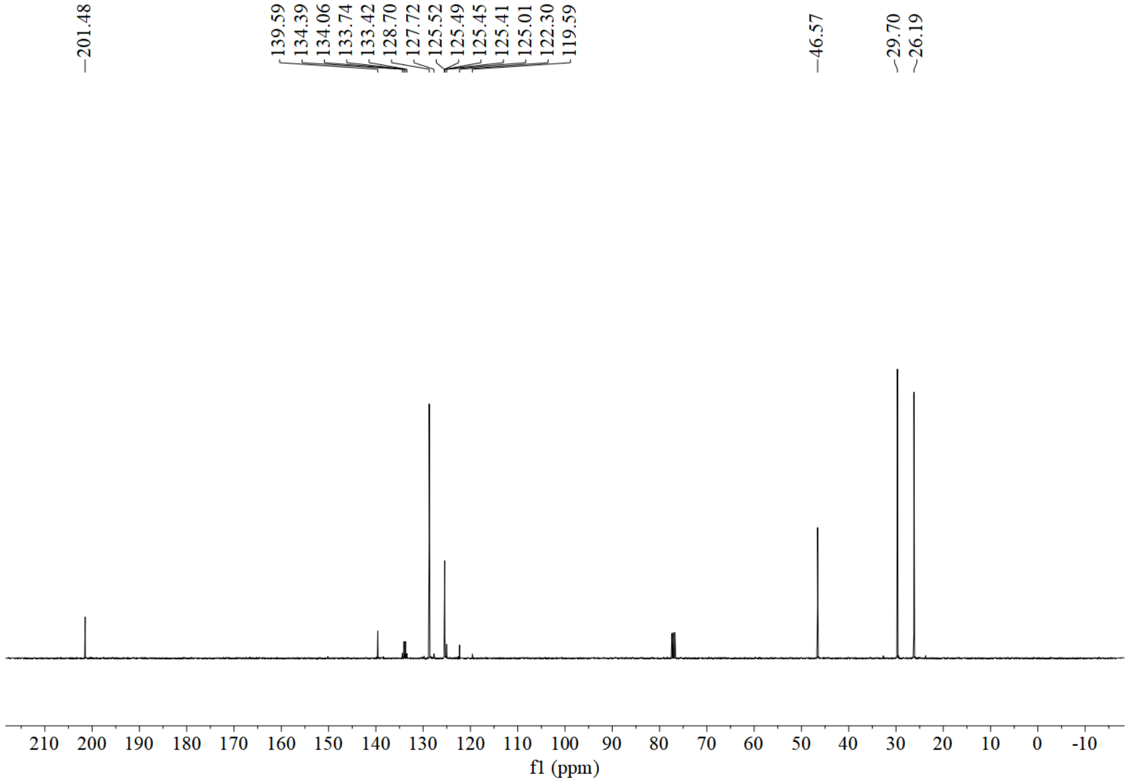

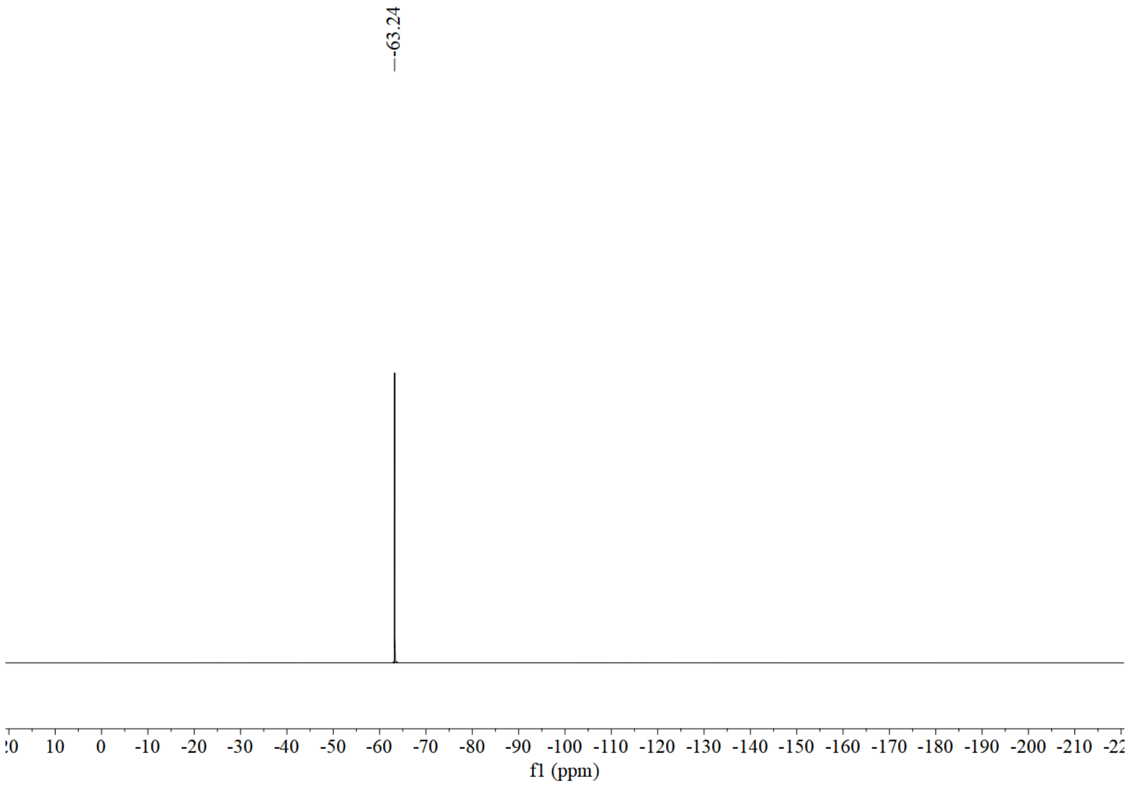
-63.24
20 10 0 -10 -20 -30 -40 -50 -60 -70 -80 -90 -100 -110 -120 -130 -140 -150 -160 -170 -180 -190 -200 -210
f1 (ppm)

^{1}H NMR (400 MHz) and ^{13}C NMR (101 MHz) in $CDCl_3$ of Cyclopentyl(3,5-dimethoxyphenyl)methanone (**3ai**)

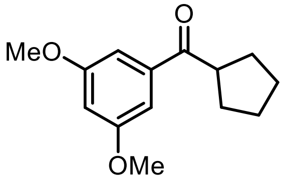

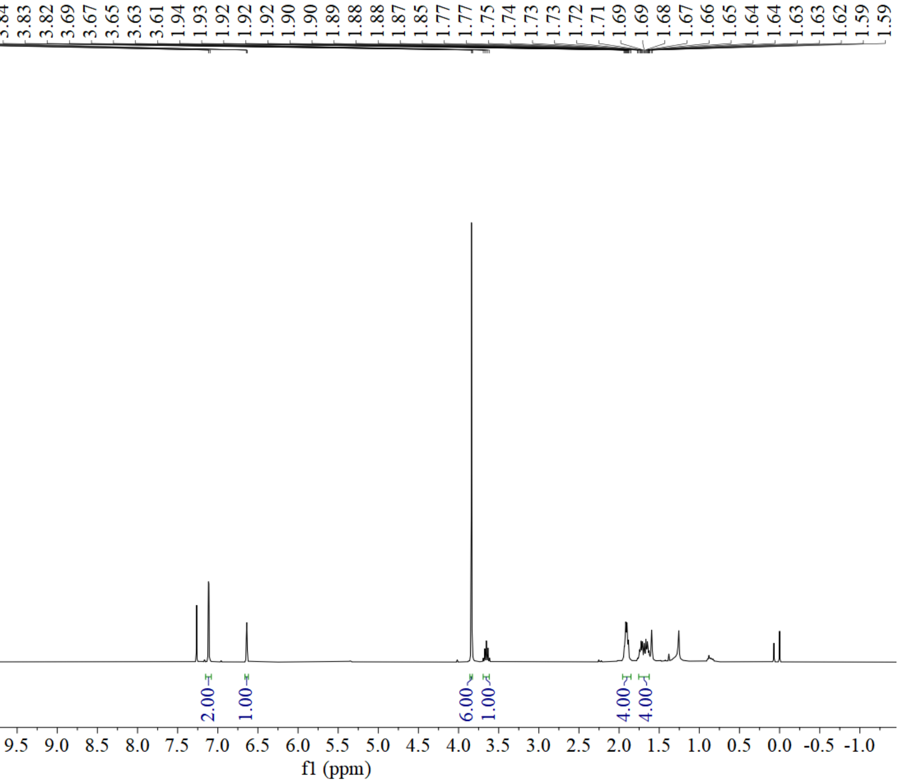

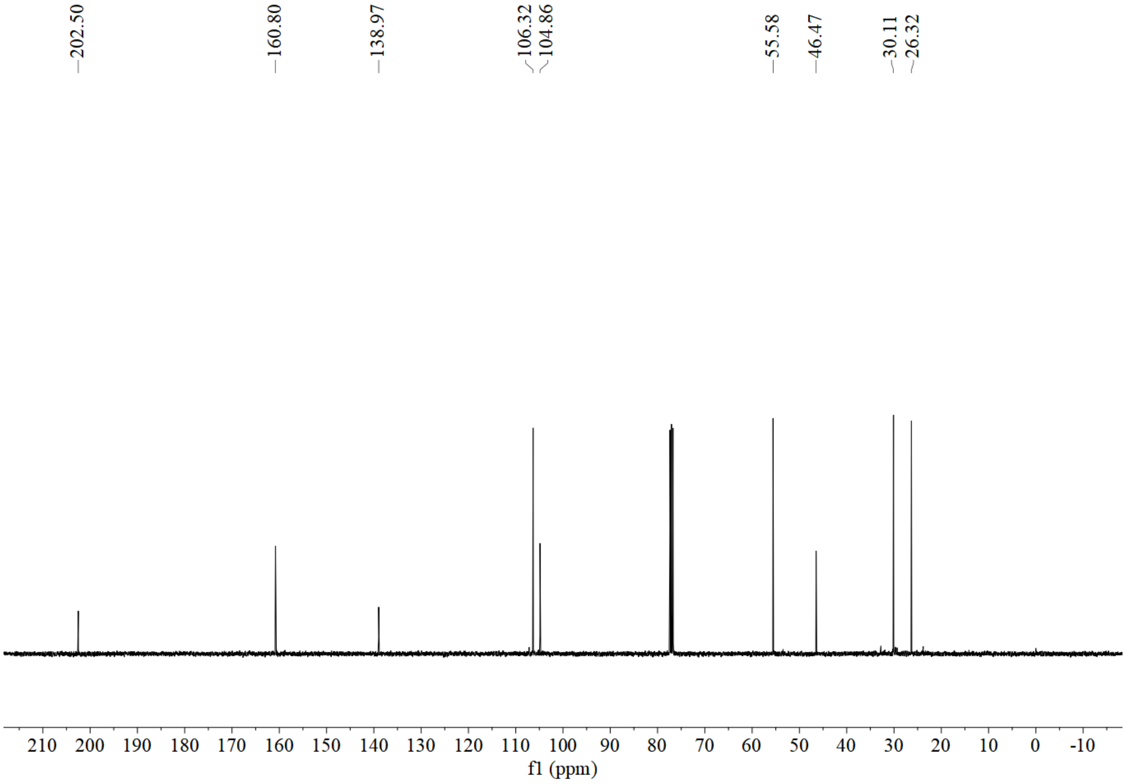

^{1}H NMR (400 MHz) and ^{13}C NMR (101 MHz) in $CDCl_3$ of Cyclopentyl(5,6,7,8-tetrahydronaphthalen-2-yl)methanone (**3aj**)

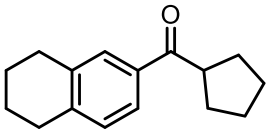

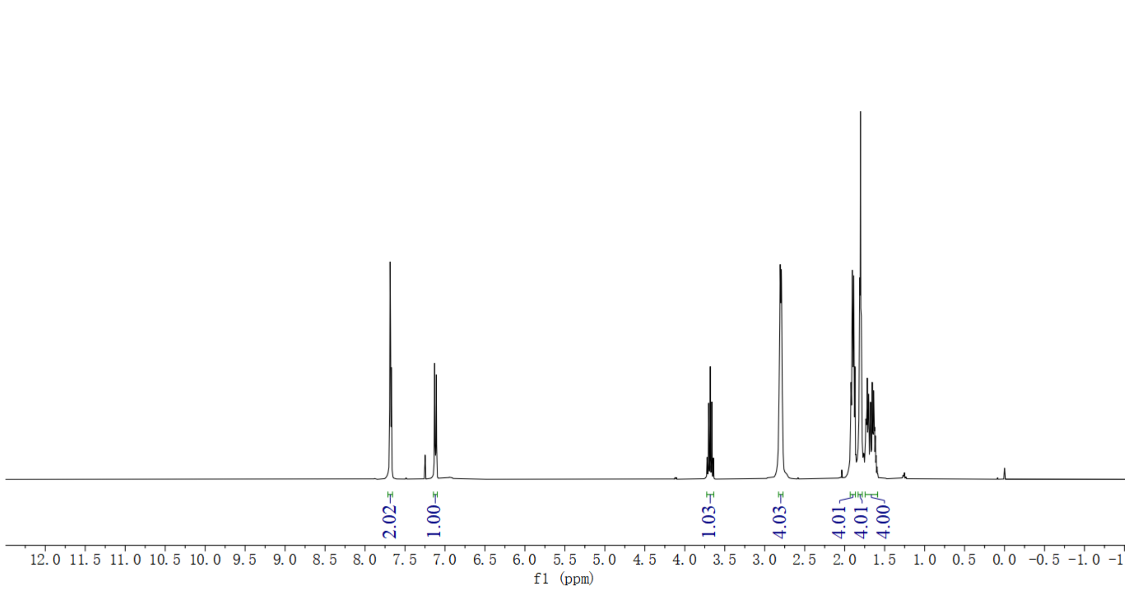

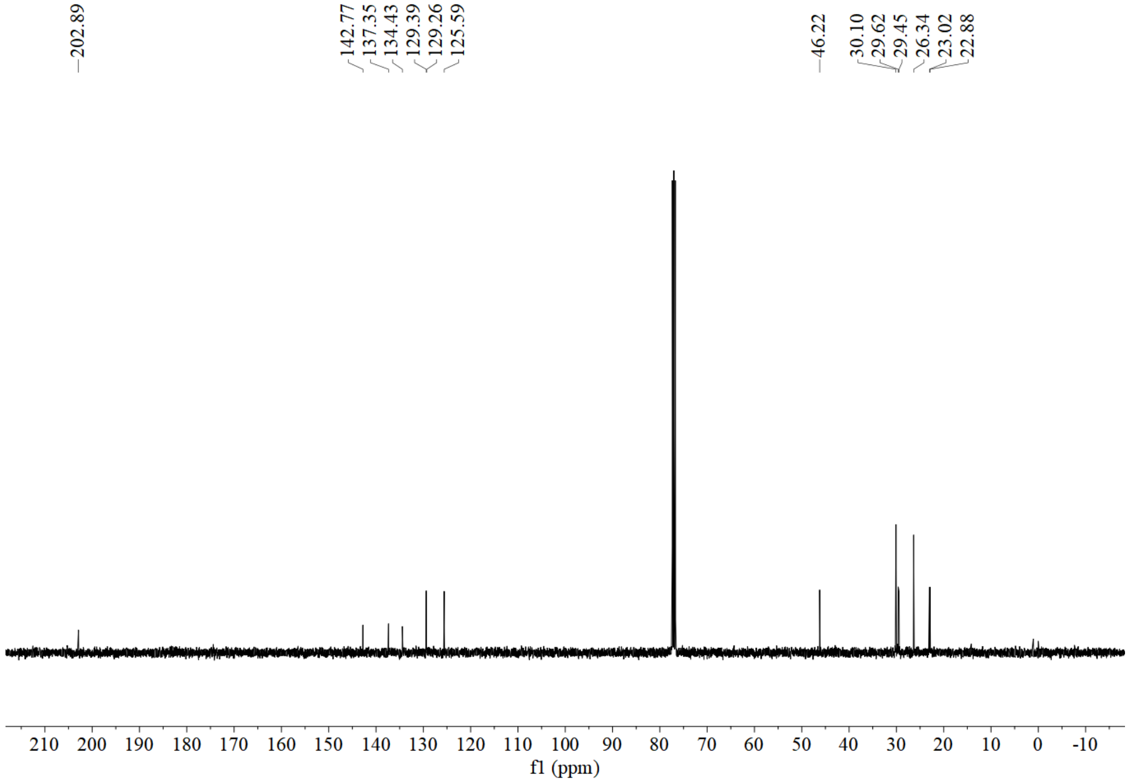

^{1}H NMR (400 MHz) and ^{13}C NMR (101 MHz) in $CDCl_3$ of 1-(5,6,7,8-Tetrahydronaphthalen-2-yl)butan-1-one (**3ak**)

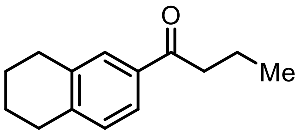

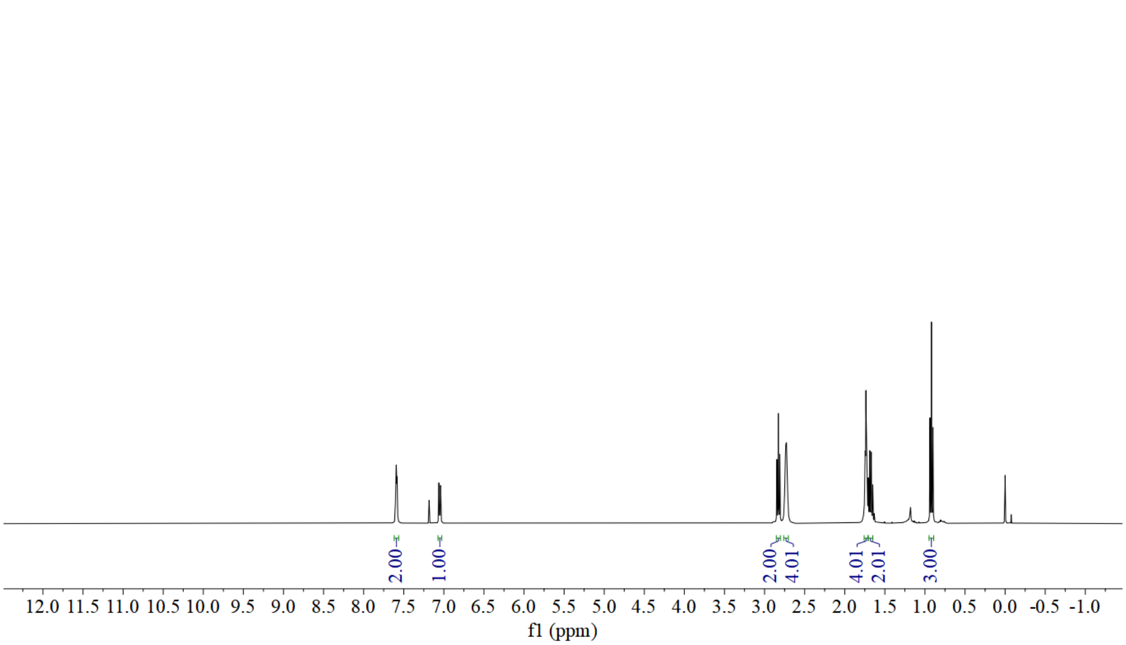

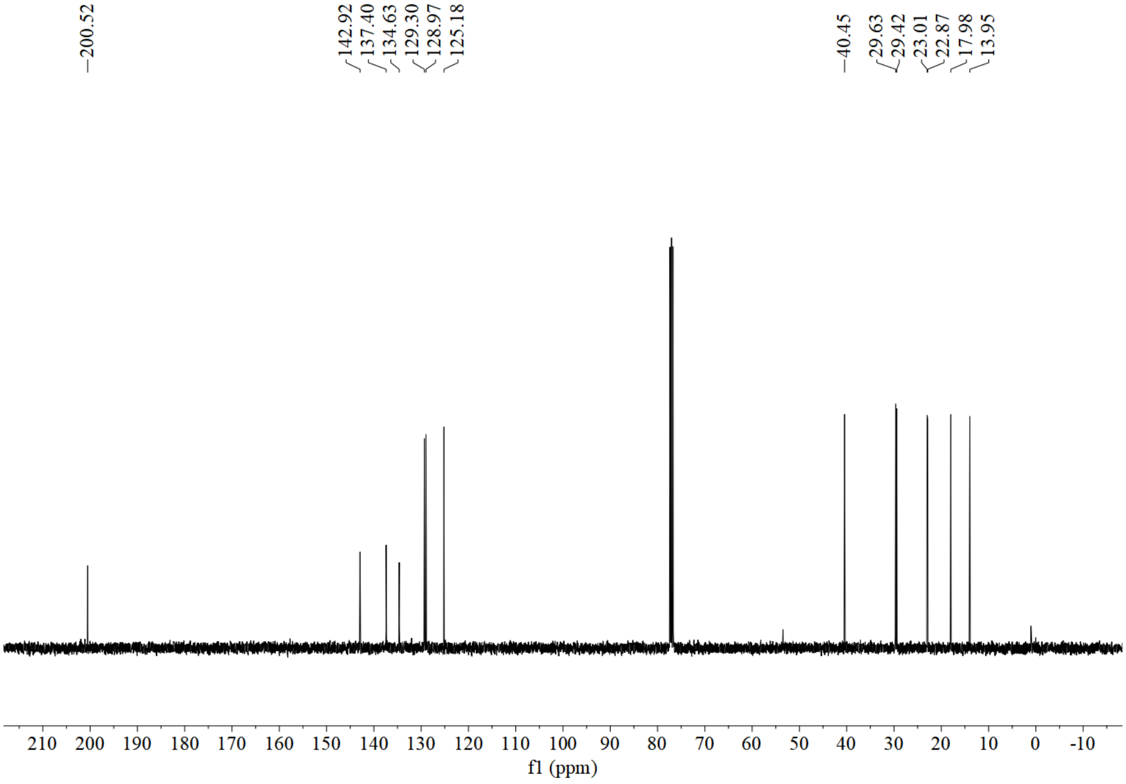

^{1}H NMR (400 MHz) and ^{13}C NMR (101 MHz) in $CDCl_3$ of 1-(1-Methyl-1H-indol-5-yl)butan-1-one (**3al**)

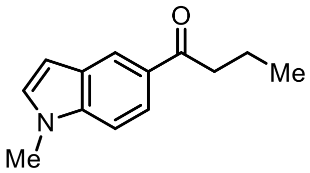

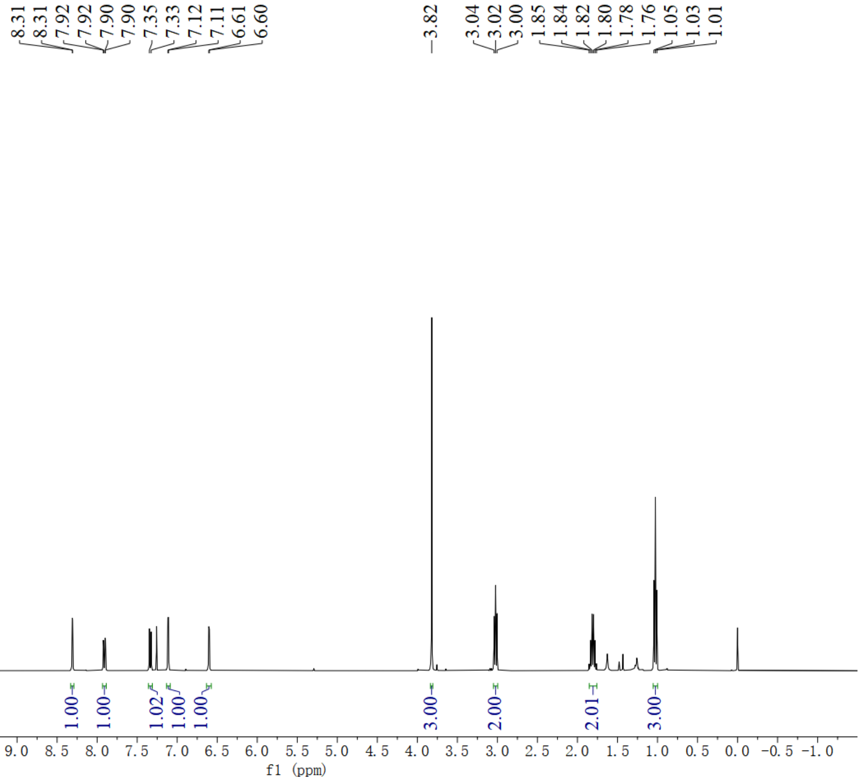

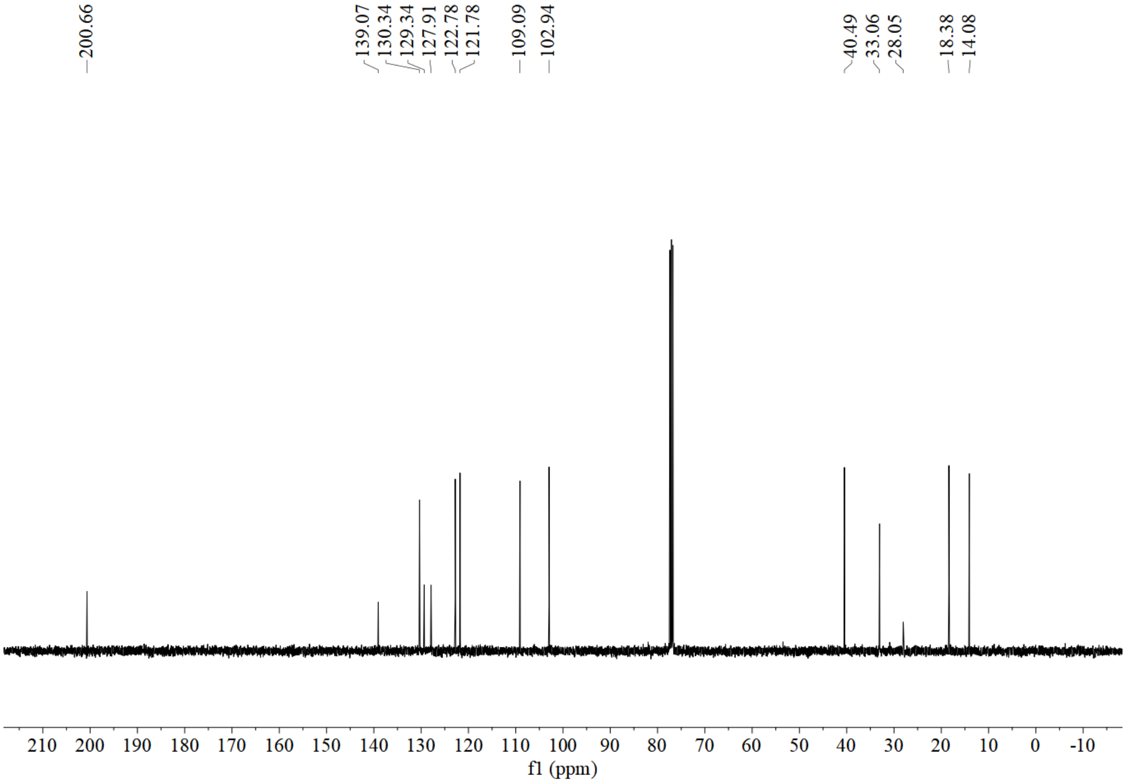

^{1}H NMR (400 MHz) and ^{13}C NMR (101 MHz) in $CDCl_3$ of 2-Phenyl-1-(*m*-tolyl)ethan-1-one (**3u**, gram-scale)

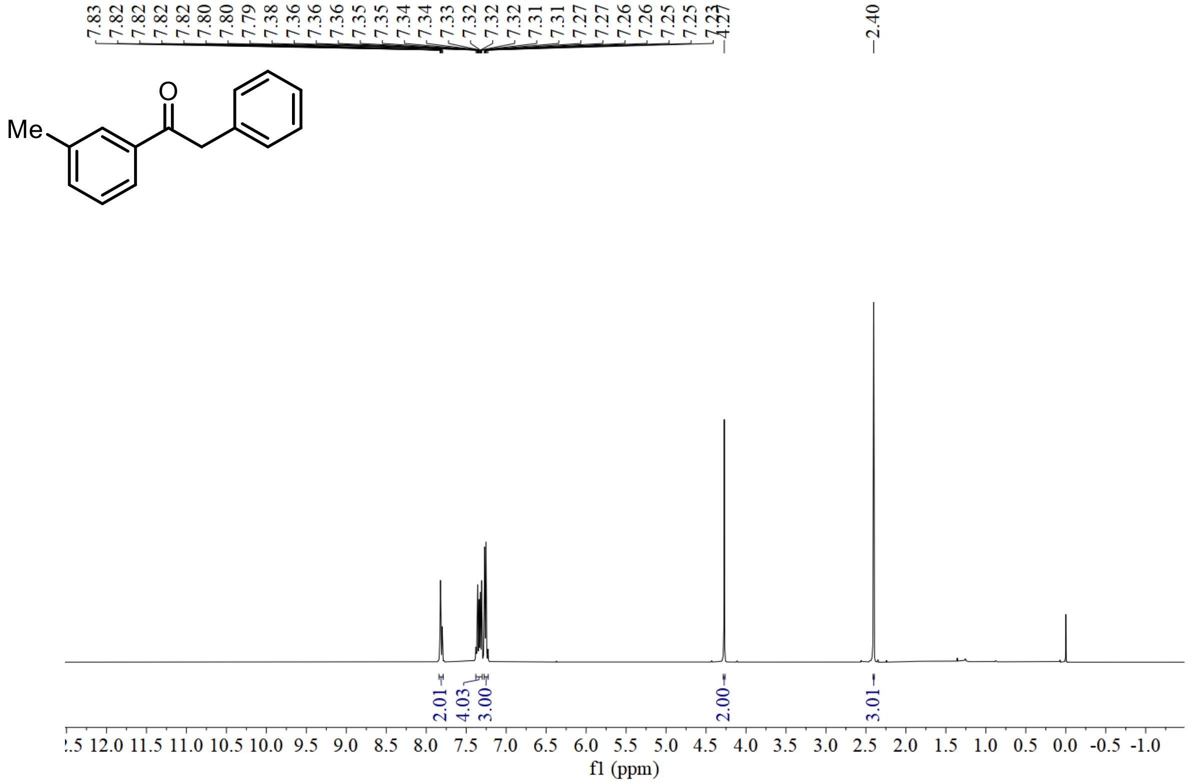

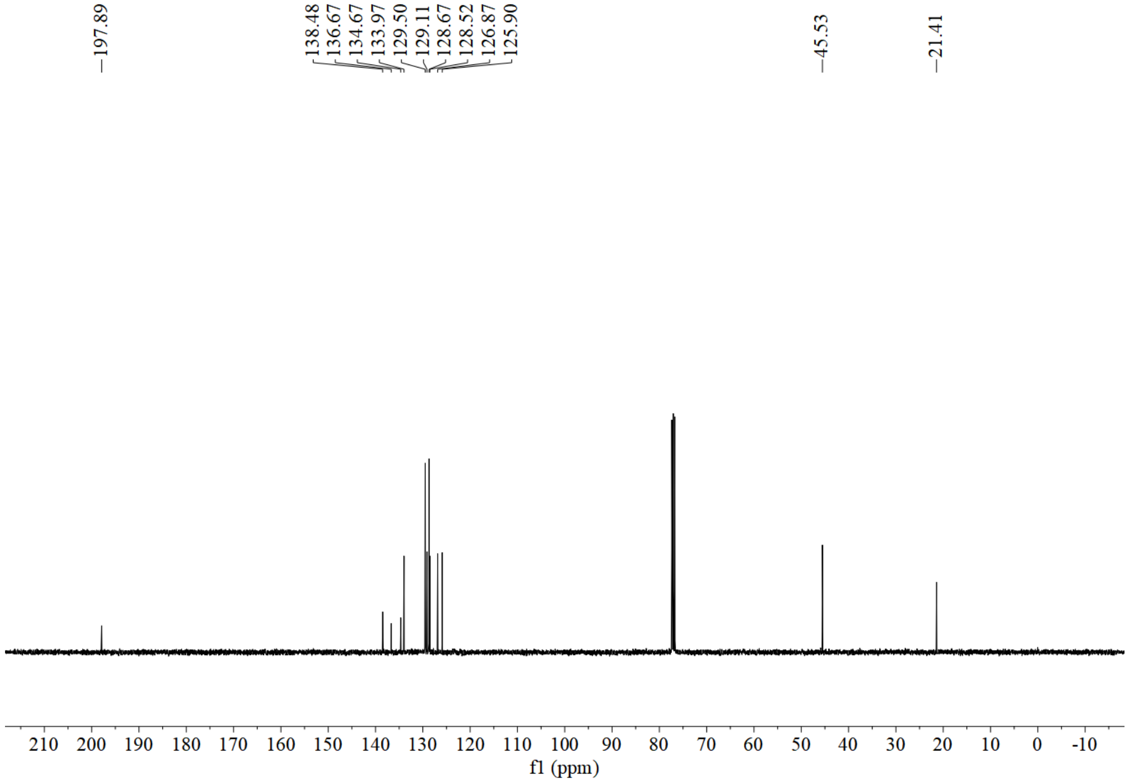

^{1}H NMR (400 MHz) and ^{13}C NMR (101 MHz) in $CDCl_3$ of 3-(4-phenyl-5-(*m*-tolyl)oxazol-2-yl)propanoic acid (**4u**)

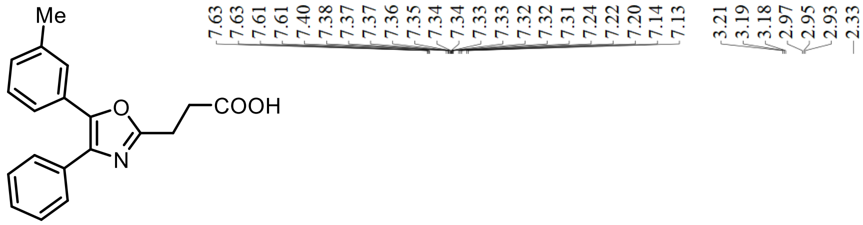

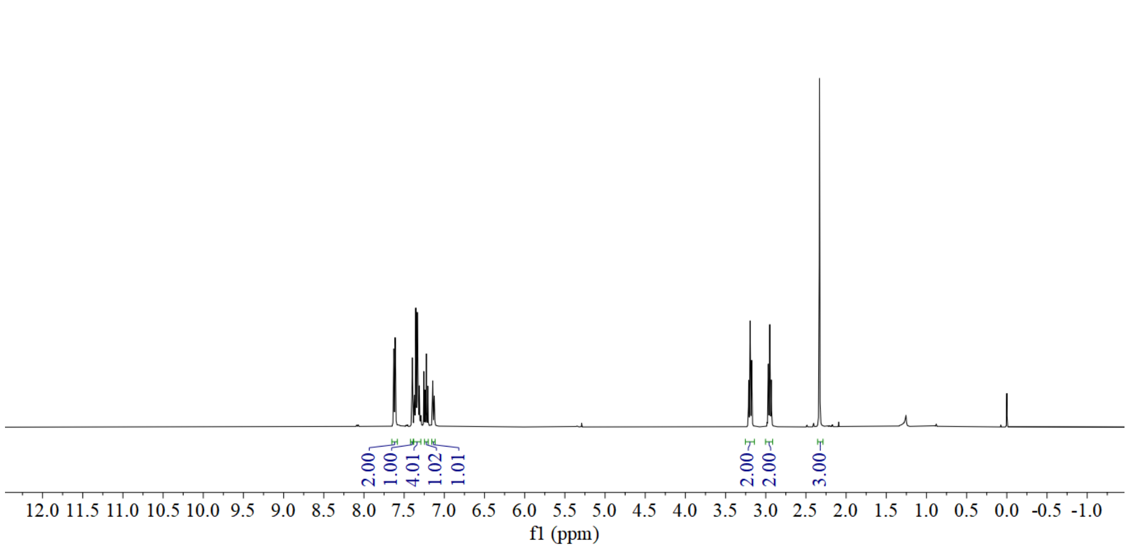

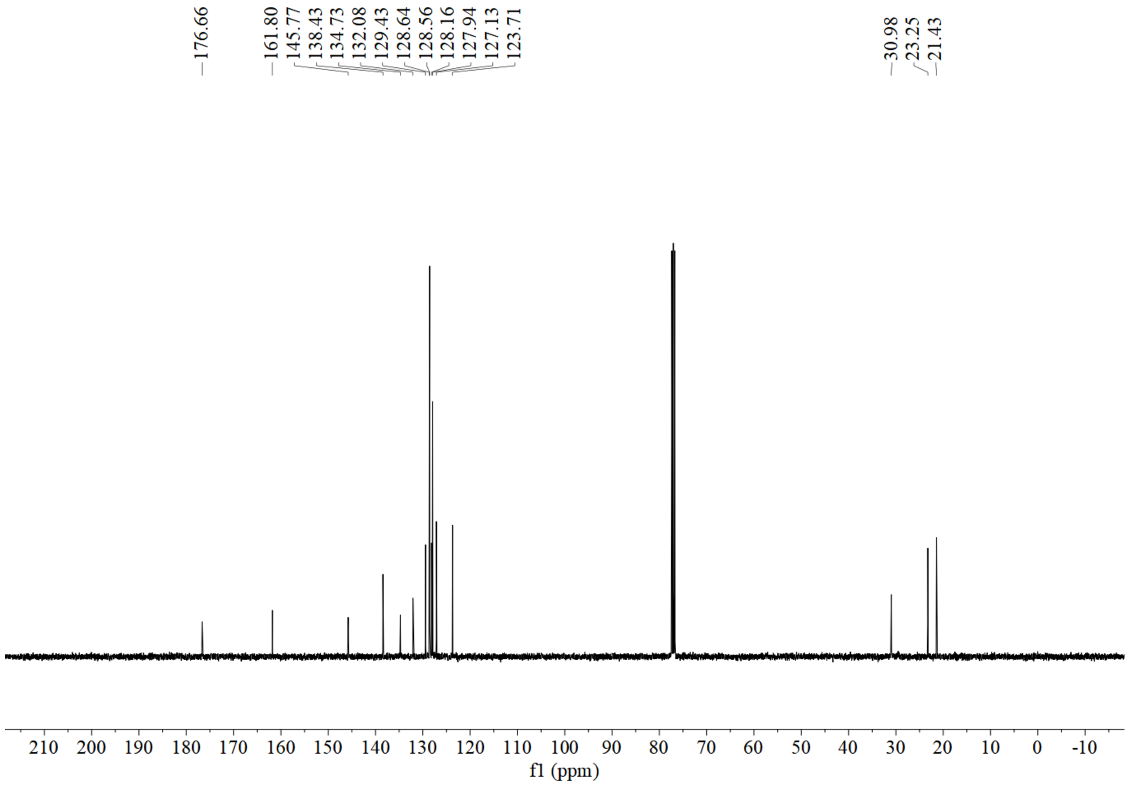

^{1}H NMR (400 MHz) and ^{13}C NMR (101 MHz) in DMSO-d_6 of (*E*)-3-hydroxy-*N'*-(1-(5,6,7,8-tetrahydronaphthalen-2-yl)butylidene)-2-naphthohydrazide (**4ak**)

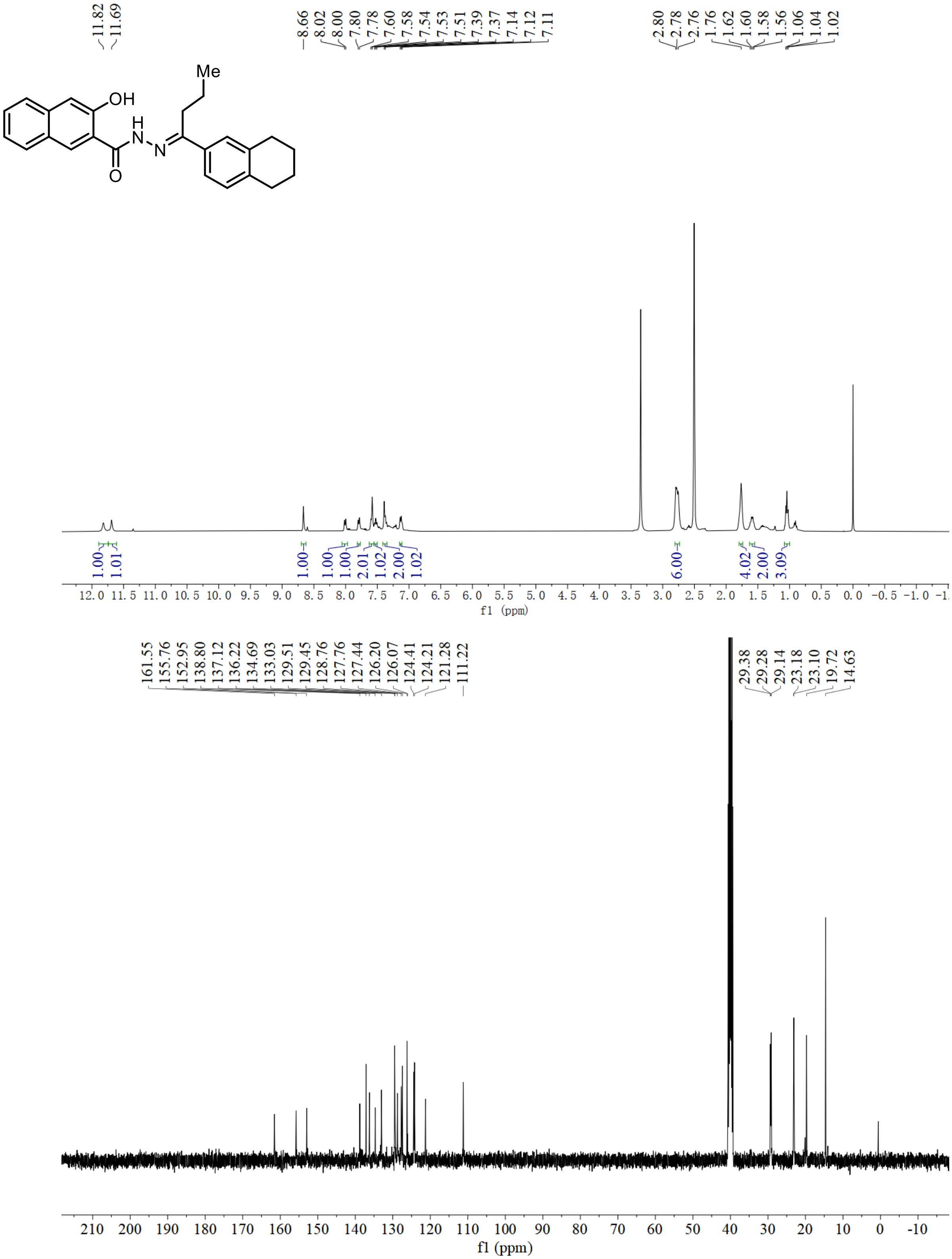

^{1}H NMR (400 MHz) and ^{13}C NMR (101 MHz) in $CDCl_3$ of 1,2-Diphenylethan-1-one (**3a**, one-pot)

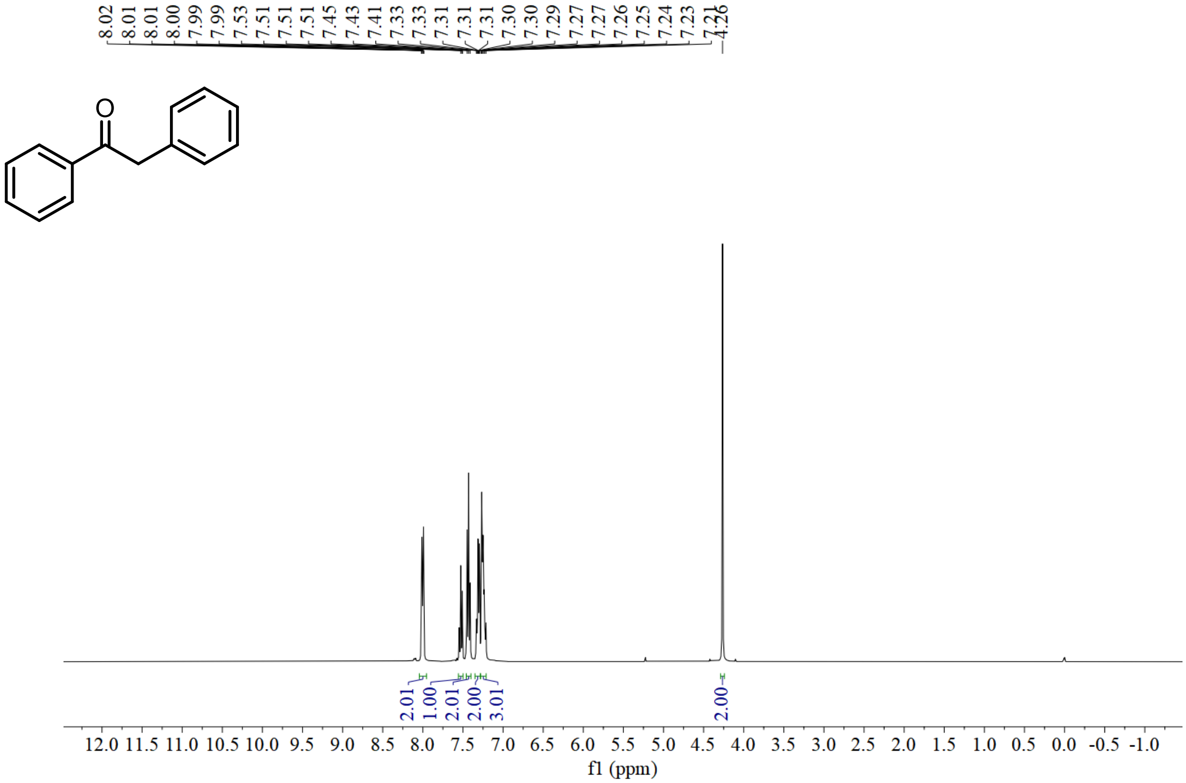

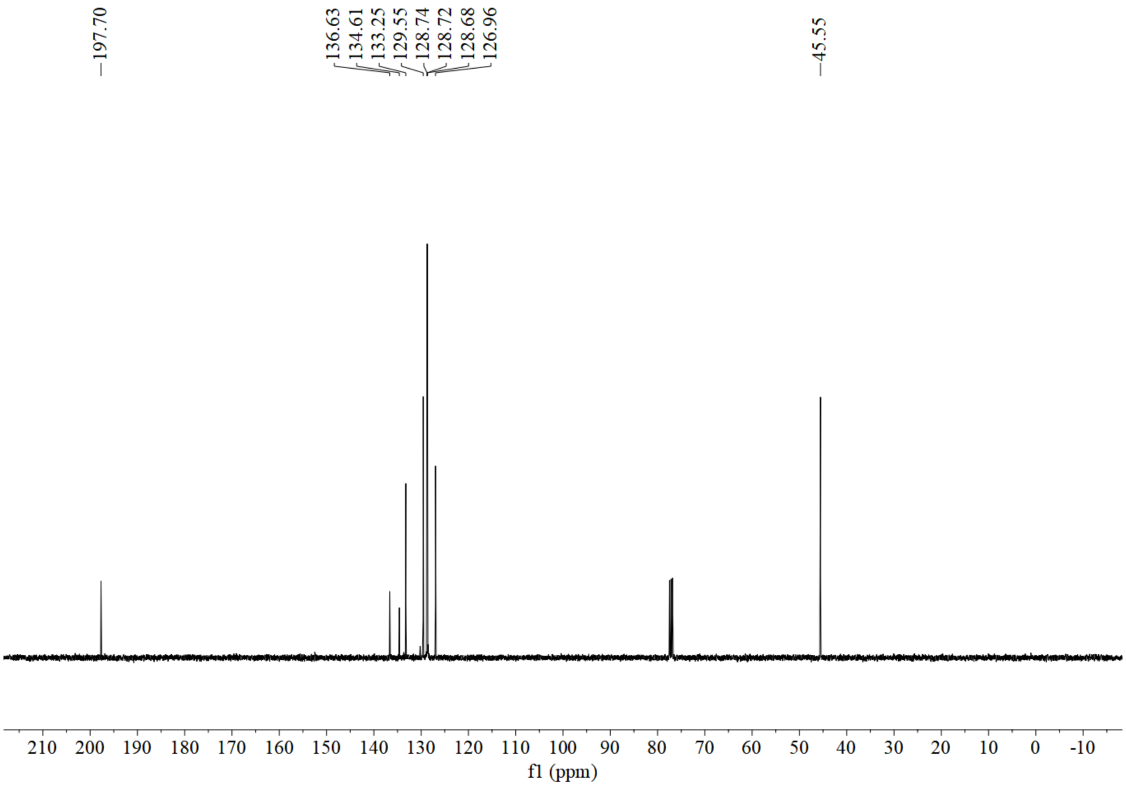

Supplement: Supplementary file 1 — Supporting Information [file ADVS-12-e11827-s001.pdf]
